# Medical Student Portfolios: A Systematic Scoping Review

**DOI:** 10.1177/23821205221076022

**Published:** 2022-03-03

**Authors:** Rei Tan, Jacquelin Jia Qi Ting, Daniel Zhihao Hong, Annabelle Jia Sing Lim, Yun Ting Ong, Anushka Pisupati, Eleanor Jia Xin Chong, Min Chiam, Alexia Sze Inn Lee, Laura Hui Shuen Tan, Annelissa Mien Chew Chin, Limin Wijaya, Warren Fong, Lalit Kumar Radha Krishna

**Affiliations:** 1 Yong Loo Lin School of Medicine, National University of Singapore, Singapore; 2 Division of Supportive and Palliative Care, National Cancer Centre Singapore, Singapore; 3 Division of Cancer Education, National Cancer Centre Singapore, Singapore; 4 Medical library, National University of Singapore libraries, Singapore; 5 Duke-NUS Medical School, National University of Singapore, Singapore; 6 Division of Infectious Disease, Singapore General Hospital, Singapore; 7 Department of Rheumatology and Immunology, Singapore General Hospital, Singapore; 8 Centre of Biomedical Ethics, National University of Singapore, Singapore; 9 PalC, The Palliative Care Centre for Excellence in Research and Education, Singapore; 10 Palliative Care Institute Liverpool, Academic Palliative & End of Life Care Centre, University of Liverpool, United Kingdom; 11 Cancer Research Centre, University of LiverpoolLiverpool, United Kingdom

**Keywords:** medical student portfolio, medical student, portfolio, learning, assessment, reflection, curriculum

## Abstract

**Phenomenon:**

Medical Student Portfolios (MSP)s allow medical students to reflect and better appreciate their clinical, research and academic experiences which promotes their individual personal and professional development. However, differences in adoption rate, content design and practice setting create significant variability in their employ. With MSPs increasingly used to evaluate professional competencies and the student's professional identity formation (PIF), this has become an area of concern.

**Approach:**

We adopt Krishna’s Systematic Evidence-Based Approach to carry out a Systematic Scoping Review (SSR in SEBA) on MSPs. The structured search process of six databases, concurrent use of thematic and content analysis in the Split Approach and comparisons of the themes and categories with the tabulated summaries of included articles in the Jigsaw Perspective and Funnelling Process offers enhanced transparency and reproducibility to this review.

**Findings:**

The research team retrieved 14501 abstracts, reviewed 779 full-text articles and included 96 articles. Similarities between the themes, categories and tabulated summaries allowed the identification of the following funnelled domains: Purpose of MSPs, Content and structure of MSPs, Strengths and limitations of MSPs, Methods to improve MSPs, and Use of E-portfolios.

**Insights:**

Variability in the employ of MSPs arise as a result of a failure to recognise its different roles and uses. Here we propose additional roles of MSPs, in particular, building on a consistent set of content materials and assessments of milestones called micro-competencies. Whislt generalised micro-competencies assess achievement of general milestones expected of all medical students, personalised micro-competencies record attainment of particular skills, knowledge and attitudes balanced against the medical student’s abilities, context and needs. This combination of micro-competencies in a consistent framework promises a holistic, authentic and longitudinal perspective of the medical student’s development and maturing PIF.

## Introduction

At a time when medical education is embracing a more personalised approach to knowledge attainment, skills training and development of professional behaviours, portfolios promise a means for medical students to better understand, reflect upon and actively shape their learning and development^1^. Complementing traditional assessment methods with wider longitudinal appraisals of an individual’s growth, portfolios add a personalised dimension to logbooks^4,5^, by serving as a repository for written examinations, tutor-rating reports and bedside assessments^6^ as well as individual reflections and analyses. 

Indeed, portfolios offer medical students “*a self-regulated, cyclical process in which [they may] mentally revisit their actions, analyse them, cogitate alternatives, [and] try out alternatives in practice”*^7^. It is this platform to showcase individual educational, research, ethical, personal and professional development^1,8^, and guide specific, holistic and timely feedback and remediation throughout the individual’s medical education that underscores growing interest in portfolio use among medical students (henceforth medical student portfolios or MSPs)^4,12^. However, despite their growing traction^13^, MSPs show significant variability in their structure and content. With local, practical, sociocultural, educational and healthcare considerations prioritising different types of data, the role of MSPs remains limited.

### Need for the Review

With MSPs representing a sustainable and effective educational undertaking that provides insight into the medical student’s development, needs, values and beliefs that may guide their professional identity formation (PIF), better understanding of the principles behind their use, the key elements within them and a framework for consistent utilisation is required.

## Methods

To determine what is known about MSPs, a systematic scoping review (SSR) is proposed to study current literature to enhance understanding of their roles and structure. These insights will also help guide the design of a consistent framework for MSPs to be used across different settings, purposes and specialities given their ability to evaluate data^14^ from *“various methodological and epistemological traditions”*^19^.

To overcome SSR’s variable methodological steps, guidance and standards, this review adopts the Systematic Evidence Based Approach (SEBA)^20^. A SEBA guided SSR (henceforth SSR in SEBA) facilitates the synthesis of an evidence-based, accountable, transparent, and reproducible analysis and discussion.

Steering this process and boosting accountability, oversight, and transparency, this SSR in SEBA sees an expert team involved in all stages of this review. The expert team comprised of medical librarians, local educational experts, and clinicians.

SSRs in SEBA are built on a constructivist perspective acknowledging the personalised, reflective, and experiential aspect of medical education and recognising the influence of particular clinical, academic, personal, research, professional, ethical, psychosocial, emotional, legal and educational factors upon the medical student’s learning journey, professional development and personal growth^27^.

**Figure 1.**
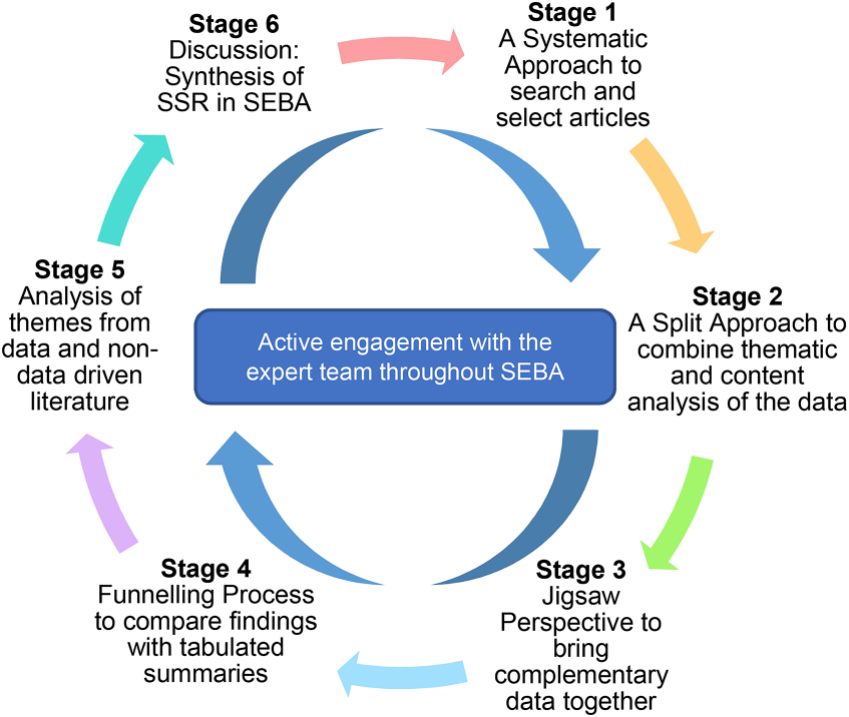
The SEBA process.

To operationalise the SSR in SEBA, the research team adopted the principles of interpretivist analysis to enhance reflexivity and discussions^18,32^ in the six stages outlined in *Figure 1*.

(Insert Figure 1. The SEBA Process)

### Stage 1 of SEBA: Systematic Approach

1. Determining the title and background of the review

The expert and research teams determined the overall goals of the SSR and the population, context and concept to be evaluated.

2. Identifying the research question

Guided by the PCC (population, concept and context), the expert and research teams agreed upon the research questions. The primary research question was “*what is known about medical student portfolios*?”. The secondary questions were “*what are the components of MSPs*?”, “*how are MSPs implemented*?” and “*what are the strengths and weaknesses of MSPs*?”.

3. Inclusion criteria

All peer reviewed articles, reviews and grey literature published from first January 2000 to 31^st^ June 2021 were included in the PCC and a PICOS format was adopted to guide the research processes^35,36^. The PICOS format is found in *Table 1*.

**Table 1.** PICOS, inclusion and exclusion criteria.

**PICOS**	INCLUSION CRITERIA	EXCLUSION CRITERIA
Population	Undergraduate and postgraduate medical students	Allied health specialties such as Pharmacy, Dietetics, Chiropractic, Midwifery, Podiatry, Speech Therapy, Occupational and Physiotherapy Non-medical specialties such as Clinical and Translational Science, Alternative and Traditional Medicine, Veterinary, Dentistry
Intervention	The use of portfolios for medical students	
Comparison	Comparison of the various use of portfolios (approaches, modalities, processes, objectives, motivations, challenges, facilitating characteristics/resources)	
Outcome	Approaches, modalities, processes, objectives, motivations, challenges, facilitating characteristics/resources in the current and potential uses of portfolios Impact of the use of portfolios on medical students	
Study design	Articles in English or translated to English Grey literature, case reports and series, ideas, editorials and commentaries Electronic and print information not controlled by commercial publishing All study designs including: Mixed methods research, meta-analyses, systematic reviews, randomised controlled trials, cohort studies, case-control studies, cross-sectional studies, descriptive papers Date of Publication: Jan 2000 – June 2021	

4. Searching

A search on six bibliographic databases (PubMed, Embase, PsycINFO, ERIC, Google Scholar and Scopus) was carried out between first to 10^th^ September 2021. Limiting the inclusion criteria was in keeping with Pham et al’s (2014) approach to ensuring a sustainable research process^37^. The search process adopted was structured along the processes set out by systematic reviews.

5. Extracting and charting

Using an abstract screening tool, members of the research team independently reviewed the titles and abstracts identified by each database to identify the final list of articles to be reviewed. Sambunjak et al’s (2010) approach to ‘negotiated consensual validation’ was used to achieve consensus on the final list of articles to be included^38^. The six members of the research team independently reviewed all the articles on the final list, used the Medical Education Research Study Quality Instrument (MERSQI)^39^ and the Consolidated Criteria for Reporting Qualitative Studies (COREQ)^40^, discussed them online and were in consensus that none should be excluded (Supplementary File 1).

### Stage 2 of SEBA: Split Approach

Three teams of researchers simultaneously and independently reviewed the included full-text articles. Here, the combination of independent reviews by the various members of the research teams using two different methods of analysis provided triangulation^41^, while detailing the analytical process improved audits and enhanced the authenticity of the research^42^.

The first team summarised and tabulated the included full-text articles in keeping with recommendations drawn from Wong et al’s (2013) “*RAMESES publication standards: meta-narrative reviews”*^43^ and Popay et al’s (2006) *“Guidance on the conduct of narrative synthesis in systematic reviews”*^44^. The tabulated summaries served to ensure that key aspects of the included articles were not lost (Supplementary File 1).

Concurrently, the second team of three trained reviewers analysed the included articles using Braun & Clarke’s (2006) approach to thematic analysis^45^. In phase one, the research team carried out independent reviews, actively reading the included articles to find meaning and patterns in the data. In phase two, ‘codes’ were constructed from the ‘surface’ meaning and collated into a code book to code and analyse the rest of the articles using an iterative step-by-step process. As new codes emerged, these were associated with previous codes and concepts. In phase three, the categories were organised into themes that best depict the data. An inductive approach allowed themes to be “*defined from the raw data without any predetermined classification”*. In phase four, the themes were refined to best represent the whole data set. In phase five, the research team discussed the results of their independent analysis online and at reviewer meetings. ‘Negotiated consensual validation’ was used to determine a final list of themes.

A third team of three trained researchers employed Hsieh & Shannon’s approach to directed content analysis and independently analysed the included articles^46^. This analysis using involved “*identifying and operationalising a priori coding categories*”. The first stage saw the research team draw categories *from* Davis et al.’s (2001) *“AMEE Medical Education Guide No. 24: Portfolios as a method of student assessment”*^47^ to guide the coding of the articles. Data not captured by these codes were assigned a new code in keeping with deductive category application. Categories were reviewed and revised as required. In the third stage, they discussed their findings online to achieve consensus on the final codes. These final codes were compared and discussed with the final author.

### Stage 3 of SEBA: Jigsaw Perspective

As part of the reiterative process, the themes and categories identified were discussed with the expert team. Here, the themes and categories were viewed as pieces of a jigsaw puzzle and areas of overlap allowed these pieces to be combined to create a wider/holistic view of the overlying data. The combined themes and categories are referred to as themes/categories.

Creating themes/categories relied on use of Phases 4 to 6 of France et al.’s (2016) adaptation^48^ of Noblit and Hare's (1998) seven phases of meta-ethnography^52^. To begin, the themes and categories were contextualised by reviewing them against the primary codes and subcategories and/or subthemes they were drawn from. Reciprocal translation was used to determine if the themes and categories could be used interchangeably.

### Stage 4 of SEBA: Funnelling Process

To provide structure to the Funnelling Process, we employed Phases 3 to 5 of the adaptation. We described the nature, main findings, and conclusions of the articles. These descriptions were compared with the tabulated summaries. Adapting Phase 5, reciprocal translation was used to juxtapose the themes/categories identified in the Jigsaw Perspective with the key messages identified in the summaries. These verified themes/categories then form the line of argument in the discussion synthesis.

## Results

**Figure 2.**
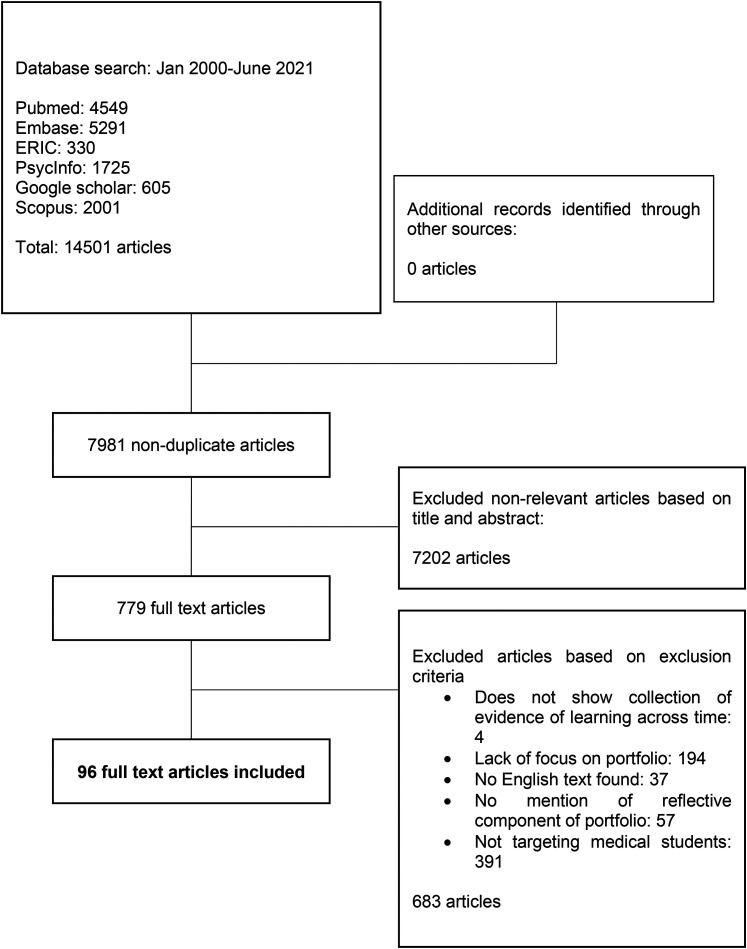
PRISMA flow chart.

A total of 14501 abstracts were reviewed, 779 full text articles were evaluated, and 96 articles were included (see *Figure 2*.). The funnelled domains identified were: Purpose of MSPs, Content and structure of MSPs, Strengths and limitations of MSPs, Methods to improve MSPs, and Use of E-portfolios.

Funnelled Domain 1: Purpose of MSPs

The purpose behind the employ of MSPs are often poorly explained and have been summarised in *Table 2* for ease of review.

**Table 2.** Purpose of MSPs.

CONTENT	ELABORATION AND/OR EXAMPLES
Learning	Reflective learning:^7,11,47,53,54,56,57,61,62,64,66,68,69,72,74,75,79,80,86,87,91,101,108^ Links practical experience with pre-existing medical knowledge^108^ Collection and selection of work samples to allow for reflection and analysis of learning^125^ Provides a platform to express inner dialogue^7^ Self-directed learning^7,11,53,54,62,64,65,67,71,73,91,101,102,105,109,115,117,126^ Identify personal learning needs^11,115^ Individualise learning goals and plans^110^ Workplace-based learning^66,115,131^ Encouraged by clinical components of portfolio^66^ Group learning^130,132^
Assessment	Formative Assessment^1,3,5,7,47,60,61,68,75,76,78,84,85,101,106,116,117,124,133^ Platform to receive constructive feedback^60,68,116,134^ Summative Assessment^1,5,13,47,54,59,68,70,71,78,80,82,102,105,106,116,121,123,124,130,135,136,139^ Ensure students have met curriculum learning objectives by quantifying their performance through grades or numerical marks^5,13,47,68^ Results are utilised to inform decisions on promotion, graduation and licensing^47,54,62,68,71,106,139^ A combination of formative and summative assessment^1,47,53,106^ establishes portfolios as a “*very powerful assessment tool”*^47^ Self-Assessment^1,5,7,47,54,62,66,67,71,72,91,110,111,115,126,131,135^ Students assess their own learning^5,7,126^, strengths and weaknesses^54^ and performance^47,72,111^ Encourages positive changes in behaviour^7,62,127^ Modalities include interviews to discuss portfolio content between students and assessors^1,4,11,57,68,108,123,131,136^ or portfolio review by assessors^1,4,8,11,54,59,70,75,91,105,111,112,116,117,123,131,134,140^

Funnelled Domain 2: Content and structure of MSPs

1. Content in MSPs

Similarly, discussions on the contents of MSPs are limited and have been summarised in Table 3. The content can be broadly categorised into content provided by the institution, medical students, and feedback/assessments by other stakeholders.

**Table 3.** Content in MSPs.

CONTENT	ELABORATION AND/OR EXAMPLES
**Contributed by institution**	
**Learning objectives**	Institutions list out clear learning objectives that students can refer to as a guide for their learning^5,7,47,54,57,67,76,79,81,102,108,111,121,124,140^ Some institutions refer to professional accreditation guidelines^5,7,67,76,79,82,102,108,111^ For example, several institutions have made use of the Canadian CanMEDS framework^79,82^. Other institutions utilised descriptions of professional roles to substitute learning objectives^54,57^ as these are easier to comprehend^54^. For example, the university of Maastricht requested for its students to include evidence within their portfolio regarding their role as a ‘researcher’, ‘healthcare worker’, ‘medical expert’ and ‘person’.^54^
**Educational Resources**	Web links^72^ Graphics and streamed videos^72^ Checklists to highlight OSCE steps^72^ Training package on specific topics^72^ Reflection writing framework^68,129^ E-Learning cases^82^
**Reflective prompts**	Questions to stimulate student reflection^3,7,54,64,65,68,69,86,103,108,115,127,144^.
**Contributed by medical student**	
**Evidence of Activities**	Curriculum Vitae^54,138^ Research projects^47,55,67,80,145^ Elective reports^1,91^ Presentations^67,80,112^ Personal achievements^63,68,129^ Membership in professional societies^145^ Extracurricular activities^80^ Evidence of learning activities Learning diaries^53,65,72,144^ Case summaries, reports, discussions^11,47,67,128,131,136,146^ Logbooks^55,66,70,80,83,91,115^ Essays to document progress in meeting competency standards^47,70,105^ Group Learning Assignments^130,132^ Graded assignments Workplace Based Assessments Mini CEX^59,67,75,131^ Direct observations^59,75,85^ Multi-source feedback (MSF) assessments^59,75,99,131^ Case based discussions^59,75,85^ Patient write-ups^67^ Summative assignment and assessment grades^59,67,80,85,91^ Critical appraisals of a topic^131^ Standardised patient assessments^67^ Evidence based medicine project^67^ Posting learning outcome grades^1^ Progress test results^59^ Anatomy lab^134^ Small group assessments showcasing student’s teamwork skills^134^ Longitudinal clinical preceptorships^134^
**Evidence of reflection**	Written reflections from students^1,3,6,7,11,53,54,57,59,62,63,65,67,69,72,75,86,87,91,103,106,108,111,115,116,131,135,147^ Topics: ▪ Professional development/skills acquisition^6,54,57^ ▪ Plans for future self-development/improvement^54,57,62,131^ ▪ Personal learning goals^1,53,57,62,72,116^ Content: ▪ Patient encounters^1,86,131^ ▪ Short summaries of patients seen by the student and reflections on what they had learned in the process^1^ ▪ Learning activities^53,108^ ▪ Activities may be those conducted internally or extra-curricular activities^108^
**Evidence of self-assessment**	Performance in competencies^59,70,72,80,111,115,123^ and roles^7^ Personal strengths and weaknesses^111^ Personal learning^61,115^ and growth^47^ Professionalism^47^
**Contributed by other stakeholders (eg assessors, peers)**	
**Assessments**	Assessors Tutors^53,55,59,60,85,111,116,135,139,146^ Faculty^70,122,138^ Peer assessors^72,111,131,134^ Patients^72^ Examiners from courses taken in other faculties^63^ Domains Clinical skills/competencies^1,3,6,47,59,63,108,112,115,128,129,131,133,139^ Communication skills^111,115,117,139^ Behavioural competencies^142^ Authentic learning, referring to the learning of practical knowledge^54,128,148^ Personal and professional development^1,47,55,67,70,91,131^

2. Structure of MSPs

Standardisation within and across portfolios may be achieved through the use of a clear template^4^ or set of guidelines^53^. MSPs with clear delineation of contents required^54^ were found to boost student receptivity^55,56^ and enhanced reliability and validity during portfolio assessment^47,55,57^.

However, a flexible approach allowing medical students to personalise their MSPs^58^ and express themselves more freely^59^ facilitates portfolio student-centricity^60,61^ and ownership^53^. By encouraging students to incorporate their own content, such as reflective diary entries^55^, reflective essays^57^, video recordings^58^, audio recordings^59^, poetry or art^62^, improvements may be seen in the quantity and quality of their reflections^56^.

### Funnelled Domain 3: Strengths and Limitations of MSPs

Given the lack of elaboration, much of the data for this domain is summarised in tables to aid easy review.

1. Strengths

Strengths of MSPs are highlighted in *Table 4*.

**Table 4.** Strengths of MSPs.

STRENGTHS	ELABORATION AND/OR EXAMPLES
**Learning**	Highlights important skills and competencies *Allows medical educators to reshape and redefine core concepts of medical practice through the development of portfolio criteria* Streamlines learning and teaching focused on important competencies^4,11,53,72,80,115,123,124,133^ Stimulates learning^5,11,74,77,102,109,118,130,132,135^ Feedback provided highlights potential areas for improvement^5,6,9,66^ *“Act of logging ‘learning moments’ helped facilitate memorisation”* ^109^ May improve performance in other knowledge-based assessments^132^ Promotes development of important skills Problem solving^132^ Communication^56,63,105,111,115,121,128,131,142^ Ethical and legal responsibility^7,53,87,149^ Professional development^5,8,11,47,53,56,63,74,78,86,103,105,111,116,123,131,135,141,142^ Teamwork^63,87,111,130,132,135,142^ Critical thinking^121^
**Assessment**	Examiners and faculty generally accept portfolios^6,60,65,74,114,116,150^ and their assessment methods^1,6,116,135,140^ as they are: Individualised^47,55,63,117^ Portfolio assessment can cater to a range of learning styles^117^ because it can be easily personalised based on the student^55^. Unique evidence may be selected to express their capabilities to examiners^63^. Comprehensive^1,54,61,70,83,117,123,126,135,137,140^ *“Combines information from both subjective and objective assessment procedures ‘to see the whole picture’”* ^140^ Able to evaluate competencies that are otherwise not easily assessed^1,54,83^ such as professionalism^123,137^ Longitudinal^1,47,67,74,80,99,117,133,141^ Portfolios are assembled over a period of time and hence can be used to monitor student’s progress over the period of compilation Educational Use in assessment has helped stimulate learning^1,66,73,74^ Guides tailored teaching by faculty members^54,91,126,133,134^ Guides remediation plans for underperforming students^1,62,91,105,111,116,135,140,142^ Specific to summative portfolio assessment: Ensures that students take the portfolio exercise seriously^57,114^ Students will be spurred on to improve themselves should they receive negative feedback^75^ Better demonstrates achievement in competencies such as professionalism, teamwork, and communication skills^111^ Specific to formative portfolio assessment: Enables constant improvement through feedback and reflection^6,7,60,71,75,105,116,127,133,140^ Fosters self-motivation^5,69^ and intrinsic motivation to reflect^91,115^.
**Others**	Encourages students to discuss their private thoughts^103^ Prepares students for postgraduate work Easily transferable when needed in the future^80^ to facilitate job applications^103,104^ or acquisition of letters of recommendation for future training^80^ Helps to ease transition to postgraduate educational practice^74^ as portfolios and portfolio assessment are often utilised at postgraduate level^55^ Improves teaching within undergraduate programs Improves faculty’s understanding of students ▪ Better understand students’ thinking and attitudes^65^ ▪ Directs discussion during meetings with advisees^65,74^ Identifies gaps in the curriculum^56,101^ such as through providing opportunities for students to evaluate teaching activities^56^ Helping students to develop better rapport with others including patients^62,118,122^, clinical teams^62^ and other students^132^

2. Limitations

The limitations of MSPs are highlighted in *Table 5*.

**Table 5.** Limitations of MSPs.

LIMITATIONS	ELABORATION AND/OR EXAMPLES
**Learning**	Limited use for theoretical knowledge^121^ Limited use for reflective learning Does not guarantee that reflection will take place^7,54,56,64,78,87,103^ Students are sceptical about the reflective process^53,67,68,87,110^ Challenging for individuals who are not intuitively reflective^64,72^ Overly prescriptive structure of reflective prompts may hinder reflective process^64^
**Assessment**	Limited reliability and validity^4,54,55,59,62,63,71,72,91,108,111,112,117,135,137^ Inauthentic ▪ Provide only vignettes of a student’s journey^59^, and students may hide evidence of their weaknesses^54,59,63,70,104,126^, fail to express their authentic views^63^ or even fabricate reflections^78^ ▪ They may also perform poorly under stress during assessments included in their portfolios such as directly observed work-based assessments^59,137^ ▪ Students tend to have a poor self-assessment capacity^72,111,151^ ▪ Perceived quality of portfolio relies heavily on the individual’s reflective ability^55,105,121^ which is unfavourable for students with poor reflective skills Subjective ▪ Students may create their portfolios differently based on their own interpretation of the purpose of the portfolio^59^ ▪ Student’s portfolios may unknowingly be judged on irrelevant aspects such as layout and format^4^ ▪ This may be amplified if student identity is not anonymised to examiners evaluating the portfolios^119^ Overly structured^47,53,57,59,62,64,119^ ▪ Highly structured portfolios with a rigid format can lead to students including less of their personal observations and reflections, which diminishes the portfolio’s capacity for authentic assessment of the student and their development Problematic assessment process Poor student understanding^11,53,62,63,73,104,116^ Time consuming ▪ There may be insufficient time for comprehensive assessments in the clinical setting as taking time to assess students must be balanced with providing quality patient care^59^ ▪ Time consuming for assessors^1,5,11,13,53,55,60,63,65,68,74,104,112,116,140^ ▪ Human resource intensive^6,112,137,140^ ▪ Excessive paperwork^1,55,74,106^ Lack of standardisation among examiners ▪ Poorly standardised assessment procedure leads to poor consensus among assessors^117^ Lack of training for assessors limits the use of work-based assessments within portfolios for assessing student competence^137^
**Portfolio Implementation**	Negative student sentiments Resistance^5,11,53,59,61,63,66,67,74,102,104,106,126^ ▪ Perceived to be redundant^61,102^ and incompatible with studying format^61,77,78^ Non-priority ▪ Students prioritise coursework that contributes towards their final examination marks^146^ ▪ Interference with other studies^123^, including clinical learning^91^ and time that should be spent with patients^1^ or studying for exams^78^ Poor understanding and engagement^1,4,54,61,66,74,78,108,150^ ▪ Unaware of how portfolios can be integrated into their education^110^ ▪ Stressful^78^ and difficult to fill out^61,78^ Burdensome ▪ Time consuming^11,66,79,108,115,116^ ▪ Excessive paperwork^1,55,77,102,106,108^ Worried about the negative comments they could receive from their mentors^61^ Felt the time given to complete their portfolios was too short, leading to reduced value^123^ Lack of support from mentors^64,66,110^ Not all mentors provided feedback and engaged the students^64,78,103,118^ Factors leading to faculty’s lack of support ▪ Poor time management^64^ ▪ Failure to understand role as portfolio mentors^64,110^ ▪ Did not engage in reflection personally^64^ ▪ Difficulty finding methods to help students^78^ ▪ Poor impression of portfolios and their role in education^66,78^ ▪ Poor relationship with student^103^

### Funnelled Domain 4: Methods to Improve MSPs

The potential methods to improve MSPs are highlighted in *Table 6*.

**Table 6.** Methods to improve MSPs.

METHODS	ELABORATION AND/OR EXAMPLES
**Increase Mentorship** Mentorship refers to a system where students are assigned to faculty throughout their training and portfolio creation to coach them^54,57,101^, engage them in supportive dialogue^63,64,108,118,148^, provide feedback^1,61,63,64,133^ and encourage them to fully engage with their portfolios^74,78,103,131,146^.	
Benefits of Mentorship	Crucial to portfolio success^4,7,63,64,78,79,87,104,131^ because it helps guide the students’ reflective process^57,65,131,146^, enhances learning^1,57,74,135^ and increases student receptivity towards their use ^7,64,103^
Improving quality of mentorship	Train mentors^66,78,87,123^ and utilise verified teaching methods that foster reflection^152^ and ensure mentors are able to stretch their students in their reflective practice^78^ Recruit good mentors Willing to engage students^108^ Understands reflection^129^ and their responsibility to teach students how to utilise reflections purposefully^79^ Able to build trust and rapport with students^64^
Having a structured mentoring programme to guide portfolio use	Some institutions encourage frequent weekly meetings with mentees^108^, while others believe that mentorship can occur as infrequently as two to three times a year^4,57,64^ Keep the student to mentor ratio small such as having one-to-one interactions^6,70,79^
**Encourage portfolio uptake**	
Improve understanding	Students with a better understanding of portfolio usage had more positive attitudes towards portfolios^108^ Introduce and orientate students to the portfolio^6,54,57,61,63,73,104,108^ Educate students on purpose and objectives of portfolio^62,64,67,70,101,104,123^ Provide clear instructions and portfolio guidelines^7,61,63,70,73,102,104,108,114,116,118,123,143^ Structure portfolios clearly^4,7,53,54,56,57,60,64,65,70,91,102,114,121,123^
Increase Exposure	Students who had been exposed to them for some time^6,91^ had more positive attitudes towards portfolios. Embed portfolio into the curriculum^54,64,72,104^ and encourage faculty and department staff to reference it in daily practice^77^ Early portfolio introduction^54,129^
**Structure portfolio appropriately**	
Organise portfolio based on its purpose	Organise the portfolio based on its purpose^125^. For a portfolio focused on enhancing learning, the portfolio should include more self-reflection^54,56^ and reasoned tasks that demonstrate student learning^56^. For a portfolio meant for assessment, content should mainly compose of evidence that competencies have been achieved^5^ and prompts should be minimal as the student's choice of reflection is also important in assessment^143^ If the portfolio is meant to promote reflection, design the portfolio to ensure it is conducive for reflection Provide reflective prompts^3,7,54,64,65,68,86,108,119,127,143,144^ Increase emphasis on writing reflections rather than describing activities^108^ Refrain from limiting word count^62^ Utilise innovative tools such as the visual analogue scale^151^ or audio recordings^59^ Portfolios should also be organised to facilitate effective teaching by faculty^56^
**Improving portfolio assessment process**	
Enhance learning through assessment process	Focus assessment on promoting student development^88^ through providing useful feedback^121,124^ Enhance reflective learning Ensure assessment does not compromise reflection^54^ Assess students based on the authenticity of their reflections^53^ Institute a central committee to review assessments and ensure ample learning experiences and assessment evidence exist to guide student learning^70^
Standardisation	Standardisation improves the reliability of the assessment process^8,72,116,131^ The following may be standardised Portfolio content^8,72,116,131^ Standardising assessment criteria^1,8,47,55,72,112,116,124,131,135^ including standardising portfolio interview questions^1,13^
Improve assessment procedure	Prepare students adequately for the assessment^91,105,116,131^ by providing guidelines on the purpose and format of the assessment^116^, clarifying expectations^91^, providing guidance from trained portfolio advisors^105,131^. Ensure assessment occurs immediately after a clinical experience^129^ Increase number of assessment points such as by adopting more work-based assessments within the portfolio^137^ Reduce subjectivity of assessment Create and validate clear rubrics to assist assessors in their grading of students^121^ Increase number of assessors to achieve better inter-rater reliability^62,72,112,121^ Provide training to assessors^4,53,62,64,67,68,74,85,87,104,111,121,124,135^ Providing opportunities for discussion or feedback between assessors^4,8,63,72,105,111,116,117,124^ Introduce portfolio interviews where students can discuss and elaborate upon their portfolios personally^4,8,53,72,105,116,140^ or even assess their own portfolios^5,55^
Improve self-assessment process	Encourage students to include evidence to support their self-assessments to reduce inaccurate self-assessments^111^
**Evaluate Feedback**	
Importance	Student empowerment and feedback have all been valuable tools in successful portfolios^47,53^: Allows for evaluation and alignment of portfolio with teaching, learning and assessment data^113^ Help to ensure the portfolio is being used appropriately^11,68,74^ Helps to introduce positive changes^11,47,62,78^

### Funnelled Domain 5: E-Portfolio

The electronic portfolio (e-portfolio) is a form of MSP that is hosted on electronic platforms^5,6,9,47,53,56,58,61,63^, and may be created using unique software^47,63,65,76,86^. Compared to hardcopy portfolios, they are more durable^66^, user friendly^63,75,77^, accessible^6,53,58,61,80^ collaborative^5,67,73,76,81^ and superior for assessment in certain areas^61^. Furthermore, they are able to include a wider variety of evidence including videos or website links^5,63,75,78,79^, provide increased privacy and confidentiality for users including students and coaches^67,73,86^ and allow for instant comparison between students^76^. These factors enhance their receptivity among medical students^53,61,63^.

However, accessibility may be limited by poor interface design^64,67,73,74,77,87,88^, limited administrative support^67,73,88^, poor technology^66,67,73,79^, and a lack of time or finances to upgrade and support e-portfolio technology^67^. Similarly, the lack of immediate access to computers in a clinical setting^58,66,73^, poor data security^58,65,66^, issues with communicating with mentors online^64^ or mentors not being tech-savvy^67^ also limit their applicability.

### Stage 5 of SEBA: Analysis of Evidence-Based and Non-Data Driven Literature

Evidence-based data from bibliographic databases were separated from grey literature such as opinion pieces, perspectives, editorial, letters and non-data based articles drawn from bibliographic databases and both groups were thematically analysed separately. The themes from both groups were compared to determine if there were additional themes in the non-data driven sources that could influence the narrative. In this review, the themes from the two data sources overlap, suggesting no undue influence upon the findings of this review.

### Stage 6 of SEBA: Synthesis of SSR in SEBA

The narrative produced from consolidation of the funnelled domains was guided by the Best Evidence Medical Education (BEME) Collaboration guide^89^ and the STORIES (Structured approach to the Reporting In healthcare education of Evidence Synthesis) statement^90^.

## Discussion

In answering its primary and secondary research questions, this SSR in SEBA reveals that MSPs have expanded beyond merely repositories of assessments and are now seen as a means of triangulating and contextualising assessments and their impact upon individual medical students. MSPs also allow students, faculty, and institutions to better understand the medical student’s needs, abilities, expectations, and aspirations, aiding the provision of personalised mentoring and remediation. However, to meet these wider roles, manageable^87^ and “authentic” portfolios that improve levels of engagement^91^ are key. Here, authenticity refers to the “*extent to which the outcomes measured represent appropriate, meaningful, significant and worthwhile forms of human accomplishments”*^47^ and serves to enhance the trustworthiness of what is largely qualitative data, and the validity of longitudinal assessments that help to map the development of their clinical competency^4^ and professional identity formation^4,12,92^.

However, current MSPs lack a consistent structure. While broad commonalities including learning objectives and professional expectations and roles to be met, and reflections, learning activities, self-assessments, achievements, and other evidence of competencies, MSPs vary significantly in their focus and content. Yet, these variations and particularities are unsurprising given the different practice settings, structure and program goals established by the host institution. These differences underpin the presence of different types, “depth” and nature of content prioritised. Inherent variability brought about by personalisation of longitudinal data, “*choice of materials by the student”*^54^ and “*individualised selection of evidence”*^47^, ultimately limits the use of portfolios beyond the confines of a specific institution. This lack of consistency raises concerns about the efficacy of MSPs in providing a holistic perspective of the medical student’s personal, academic, clinical, and professional development.

We believe that these concerns may be bridged in part by harnessing the ability of current MSPs to capture education and assessment in specific areas of practice. Our findings suggest that current MSPs encapsulate several entrustable professional activities (EPA)s^94^. Each EPA however shares common aspects of other EPAs that may not be directly contained within a particular MSP. We believe that it is possible to harness these overlapping aspects to make MSPs more widely applicable. Here, we build upon the notion that micro-credentialling that incorporates “circumscribed assessments” of a specific EPA, such as “interpreting and communicating results of common diagnostic and screening tests”, may be extrapolated to other EPAs such as “[communicating] in difficult situations” in a different practice setting^97^.

Hong et al’s (2021) and Zhou et al’s (2021) adaptations^98,99^ of Norcini’s (2020) concept of micro-credentialling and micro-certification in medical education^100^ which forward the concepts of generalised and personalised micro-competencies provide a viable bridge between prevailing MSP content without compromising the rich mix of structure and customisation within MSPs. Based on the certification of micro-competencies within an EPA, Zhou et al. (2021) suggest that generalised micro-competencies are the standards and expectations applicable to *all* medical students. They are small, professional learning milestones that all students need to attain before proceeding to the next competency-based stage. These are requisite knowledge, skills and attitudes all soon-to-be clinicians must have. Personalised micro-competencies, in turn, are determined by the individual’s particular goals, training, abilities, skills and experiences. They are determined by the medical student and tutors and must be consistent with institutional codes of conduct and expectations. They underscore the importance of assessing the student's individual needs and circumstances which influence which in turn shape the kind of training and support proffered. With expectations differing across practice settings and levels of training, both generalised andpersonalised micro-competencies must be clearly conveyed to the medical student and tutors in a timelyand structured manner. To encapture their learning and attainment, MSPs must forward clear learning plans to align expectations with evidence of diverse learning activities, reflective prompts and diaries, multisource formative and summative evaluations via standardised assessment tools and constructive feedback. These standardised baseline guidelines will lend clarity to portfolio developers and users. This may boost the latter’s trust and receptivity towards regular portfolio use^55,56^.

We believe that structured and consistent micro-certification of micro-competencies could be extrapolated beyond the initial goals of the MSPs and could provide a longitudinal perspective of the medical student’s development. This is especially useful when considering competencies such as interpersonal, communication skills and systems-based practices. Perhaps here, too, the silver lining to changes in medical education practices due to the COVID-19 pandemic can be harnessed.

With many institutions incorporating online learning, e-portfolios should be institutionally sanctioned^85^ with a dedicated team of portfolio developers and invested faculty members onboarding and overseeing their implementation. These considerations foreground the need for orientation sessions^10,62,64,67,104^ to educate students and faculty on the identified EPAs as well as the use of generalised and personalised micro-competencies to ensure learning and assessment congruity and objectivity^91,105,106^. Embedding the portfolios into the formal curricula, assigning students mentors trained in reflective engagement, and establishing protected time for regular portfolio reviews would help to facilitate their consistent usage. Concurrently, portfolio use must be part of a continuous quality improvement process, building on feedback^107^ and lessons learnt to promote further improvement to MSPs and portfolio assessment^10,11,47,62,78^. Indeed, both forms of micro-competencies underline the need for effective recording and oversight. This is especially important when micro-competencies provide a holistic appraisal of the medical student’s progress and achievements, needs and abilities and provides insights into their professional identity formation. Capturing this data in a comprehensive, longitudinal manner replete with the medical student’s reflections reveals a new dimension to portfolio use.

## Limitations

Firstly, the review is limited by the omission of articles not published in English. This creates the risk of missing key papers. Furthermore, the focus on papers published in English led to focus on studies in North America and Europe. 

Secondly, while the articles comment on the sentiment of users including medical students on the effectiveness of portfolios for learning and assessment, there are a limited number of articles highlighting the perspectives of doctors who previously undertook the task of undergraduate portfolios. Hence, the review is limited by its inability to assess the long-term effectiveness and acceptability of portfolio usage after medical students enter the workforce as practicing medical professionals.

## Conclusion

This SSR in SEBA reveals that if portfolios are to remain relevant and maintain their user-friendliness and accessibility, the future of MSPs must lie in improving assessments and in enhancing the manner in which they are designed.

While it is clear that assessments tools need to be enhanced to meet new perspectives of education and training, it is perhaps timely that this SSR in SEBA suggests key changes to portfolio use. In adopting e-portfolios for its accessible and expansive potential, it is clear that a robust and well-supported platform is critical. This platform ought to accommodate all manner of data and assessment results and remain a comprehensive repository of data. Categorised into different, sometimes overlapping, domains, data from this repository may be drawn to populate different designs of MSPs. Changing from one goal to another should therefore be simple. Such flexibility will still allow medical students to personalise their e-portfolios in a manner that they feel best represents their development without compromising faculty evaluation. A flexible yet robust e-portfolio such as this will also enable collaborations and facilitate input of corroborative data from third parties where required.

Moving forward, further research may be undertaken to identify the long-term effects of portfolio usage, the manner that portfolios are evaluated, and the impact it has on professional identity formation throughout and beyond medical school.

## Supplementary Material

Supplementary material

## Glossary Terms

Professional Identity Formation: An adaptive developmental process that involves the psychological development of an individual, and the socialisation of the individual into appropriate roles and participation at work.
**Krishna’s Systematic Evidence-Based Approach (SEBA** ): A structured and accountable approach used to guide analyses to ensure reproducible and robust data.
Split Approach: Combines content and thematic analysis of data to enhance the trustworthiness and depth of an analysis.
Jigsaw Perspective: Comparing overlaps between the themes and categories delineated by content and thematic analysis are considered in tandem, like complementary ‘pieces of the jigsaw’. This allows for holistic perspective of data.

## List of abbreviations

EPA: Entrustable Professional Activities
MSP: Medical Student Portfolios
PCC: Population, concept and context
SEBA: Systematic Evidence-Based Approach
SSR: Systematic Scoping Review

## References

[1] Davis MH Friedman Ben-David M Harden RM , et al. Portfolio assessment in medical students’ final examinations. Med Teach. 2001;23(4):357‐366. 12098382 10.1080/01421590120063349

[2] Fida NM Shamim MS . Portfolios in Saudi medical colleges. Why and how? Saudi Med J. 2016;37(3):245‐248. 26905344 10.15537/smj.2016.3.12937 PMC4800886

[3] Santonja-Medina F Garcia-Sanz MP Martinez-Martinez F Bo D Garcia-Estan J . Portfolio as a tool to evaluate clinical competences of traumatology in medical students. Adv Med Educ Pract. 2016;7:57‐61. 26929675 10.2147/AMEP.S91401 PMC4758781

[4] Driessen EW Overeem K van Tartwijk J van der Vleuten CP Muijtjens AM . Validity of portfolio assessment: which qualities determine ratings? Med Educ. 2006;40(9):862‐866. 16925636 10.1111/j.1365-2929.2006.02550.x

[5] Sánchez Gómez S Ostos EM Solano JM Salado TF . An electronic portfolio for quantitative assessment of surgical skills in undergraduate medical education. BMC Med Educ. 2013;13(65). 10.1186/1472-6920-13-65 PMC3651863 23642100

[6] Duque G Finkelstein A Roberts A Tabatabai D Gold SL Winer LR . Learning while evaluating: the use of an electronic evaluation portfolio in a geriatric medicine clerkship. BMC Med Educ. 2006;6(4):4. 16409640 10.1186/1472-6920-6-4 PMC1361794

[7] Fida NM Hassanien M Shamim MS , et al. Students’ perception of portfolio as a learning tool at king abdulaziz university medical school. Med Teach. 2018;40(sup1):S104‐s113. 29909725 10.1080/0142159X.2018.1466054

[8] Burch VC Seggie JL . Use of a structured interview to assess portfolio-based learning. Med Educ. 2008;42(9):894‐900. 18715487 10.1111/j.1365-2923.2008.03128.x

[9] Chiu YT Lee KL Ho MJ . Effects of feedback from near-peers and non-medical professionals on portfolio use. Med Educ. 2014;48(5):539‐540. 10.1111/medu.12445 24712959

[10] Dannefer EF Henson LC . The portfolio approach to competency-based assessment at the Cleveland Clinic Lerner College of Medicine. Acad Med. 2007;82(5):493‐502. 17457074 10.1097/ACM.0b013e31803ead30

[11] Elango S Jutti RC Lee LK . Portfolio as a learning tool: students’ perspective. Ann Acad Med Singapore. 2005;34(8):511‐514. 16205830

[12] Greenberg L Blatt B . Perspective: successfully negotiating the clerkship years of medical school: a guide for medical students, implications for residents and faculty. Acad Med. 2010;85(4):706‐709. 20354392 10.1097/ACM.0b013e3181d2aaf2

[13] Burch VC Seggie J . Portfolio assessment using a structured interview. Med Educ. 2005;39(11):1169. 10.1111/j.1365-2929.2005.02281.x 16262846

[14] Ruiz-Perez I Petrova D . Scoping reviews. Another way of literature review. Med Clin (Barc). 2019;153(4):165‐168. 30954292 10.1016/j.medcli.2019.02.006

[15] Arksey H O'Malley L . Scoping studies: towards a methodological framework. Int J Soc Res Methodol. 2005;8(1):19‐32.

[16] Armstrong R Hall BJ Doyle J Waters E . Cochrane update. 'Scoping the scope' of a cochrane review. J Public Health (Oxf). 2011;33[1]:147‐150. 21345890 10.1093/pubmed/fdr015

[17] Levac D Colquhoun H O'Brien KK . Scoping studies: advancing the methodology. Implement Sci. 2010;5(1):69. 20854677 10.1186/1748-5908-5-69 PMC2954944

[18] Schick-Makaroff K MacDonald M Plummer M Burgess J Neander W . What synthesis methodology should I Use? A review and analysis of approaches to research synthesis. AIMS Public Health. 2016;3(1):172‐215. 29546155 10.3934/publichealth.2016.1.172 PMC5690272

[19] Thomas A Lubarsky S Varpio L Durning SJ Young ME . Scoping reviews in health professions education: challenges, considerations and lessons learned about epistemology and methodology. Adv Health Sci Educ Theory Pract. 2020;25(4):989‐1002. 31768787 10.1007/s10459-019-09932-2

[20] Kow CS Teo YH Teo YN , et al. A systematic scoping review of ethical issues in mentoring in medical schools. BMC Med Educ. 2020;20(1):1‐10. 10.1186/s12909-020-02169-3 PMC7395401 32736552

[21] Bok C Ng CH Koh JWH , et al. Interprofessional communication (IPC) for medical students: a scoping review. BMC Med Educ. 2020;20(1):372. 33081781 10.1186/s12909-020-02296-x PMC7574565

[22] Ngiam LXL Ong YT Ng JX , et al. Impact of caring for terminally Ill children on physicians: a systematic scoping review. Am J Hosp Palliat Care. 2020;38(4):396–418. 32815393 10.1177/1049909120950301

[23] Krishna LKR Tan LHE Ong YT , et al. Enhancing mentoring in palliative care: an evidence based mentoring framework. Journal of Medical Education and Curricular Development. 2020;7. 10.1177/2382120520957649 PMC7517982 33015366

[24] Kamal NHA Tan LHE Wong RSM , et al. Enhancing education in palliative medicine: the role of systematic scoping reviews. Palliative Medicine & Care: Open Access. 2020;7(1):1‐11.

[25] Ong RRS Seow REW Wong RSM , et al. A systematic scoping review of narrative reviews in palliative medicine education. Palliative Medicine & Care: Open Access. 2020;7(1):1‐22.

[26] Mah ZH Wong RSM Seow REW , et al. A systematic scoping review of systematic reviews in palliative medicine education. Palliative Medicine & Care: Open Access. 2020;7(1):1‐12.

[27] Guba EG Lincoln YS . Competing paradigms in qualitative research. In: Denzin NK Lincoln YS eds. Handbook of qualitative research. Sage Publications; 1994:105‐117.

[28] Scotland J . Exploring the philosophical underpinnings of research: relating ontology and epistemology to the methodology and methods of the scientific, interpretive, and critical research paradigms. English Language Teaching. 2012;5(9):9‐16.

[29] Thomas A Menon A Boruff J Rodriguez AM Ahmed S . Applications of social constructivist learning theories in knowledge translation for healthcare professionals: a scoping review. Implement Sci. 2014;9(1):54. 24885925 10.1186/1748-5908-9-54 PMC4040365

[30] Radha Krishna LK Renganathan Y Tay KT , et al. Educational roles as a continuum of mentoring’s role in medicine – a systematic review and thematic analysis of educational studies from 2000 to 2018. BMC Med Educ. 2019;19(1):439. 31775732 10.1186/s12909-019-1872-8 PMC6882248

[31] Mack L . The philosophical underpinnings of educational research. Polyglossia. 2010;19:5–11.

[32] Pring R . The ‘false dualism’of educational research. Journal of Philosophy of Education. 2000;34(2):247‐260.

[33] Crotty M . The Foundations of Social Research: Meaning and Perspective in the Research Process. Sage; 1998.

[34] Ford K . Taking a narrative turn: possibilities, challenges and potential outcomes. OnCUE Journal. 2012;6(1):23–36.

[35] Peters M Godfrey C McInerney P Soares C Khalil H Parker D . The Joanna Briggs Institute reviewers’ manual 2015: methodology for JBI scoping reviews. 2015. http://joannabriggs.org/assets/docs/sumari/Reviewers-Manual_Methodology-for-JBI-Scoping-Reviews_2015_v1.pdf

[36] Peters MD Godfrey CM Khalil H McInerney P Parker D Soares CB . Guidance for conducting systematic scoping reviews. Int J Evid Based Healthc. 2015;13(3):141‐146. 26134548 10.1097/XEB.0000000000000050

[37] Pham MA-O Rajić A Greig JD Sargeant JM Papadopoulos A McEwen SA . A scoping review of scoping reviews: advancing the approach and enhancing the consistency. 2014(1759-2887 [Electronic]). 10.1002/jrsm.1123 PMC4491356 26052958

[38] Sambunjak D Straus SE Marusic A . A systematic review of qualitative research on the meaning and characteristics of mentoring in academic medicine. J Gen Intern Med. 2010;25(1):72‐78. 10.1007/s11606-009-1165-8 PMC2811592 19924490

[39] Reed DA Beckman TJ Wright SM Levine RB Kern DE Cook DA . Predictive validity evidence for medical education research study quality instrument scores: quality of submissions to JGIM’s medical education special issue. J Gen Intern Med. 2008;23(7):903‐907. 18612715 10.1007/s11606-008-0664-3 PMC2517948

[40] Tong A Sainsbury P Craig J . Consolidated criteria for reporting qualitative research (COREQ): a 32-item checklist for interviews and focus groups. Int J Qual Health Care. 2007;19(6):349‐357. 17872937 10.1093/intqhc/mzm042

[41] Tavakol M Sandars J . Quantitative and qualitative methods in medical education research: AMEE guide No 90: part I. Med Teach. 2014;36(9):746‐756. 24846122 10.3109/0142159X.2014.915298

[42] Cleland J Durning SJ . Researching medical education. John Wiley & Sons; 2015.

[43] Wong G Greenhalgh T Westhorp G Buckingham J Pawson R . RAMESES Publication standards: meta-narrative reviews. BMC Med. 2013;11(1):20. 23360661 10.1186/1741-7015-11-20 PMC3558334

[44] Popay J Roberts H Sowden A , et al. Guidance on the conduct of narrative synthesis in systematic reviews. A product from the ESRC methods programme Version. 2006;1:b92.

[45] Braun V Clarke V . Using thematic analysis in psychology. Qual Res Psychol. 2006;3(2):77‐101.

[46] Hsieh H-F Shannon SE . Three approaches to qualitative content analysis. Qual Health Res. 2005;15(9):1277‐1288. 16204405 10.1177/1049732305276687

[47] Friedman Ben David M Davis MH Harden RM Howie PW Ker J Pippard MJ . AMEE Medical education guide No. 24: portfolios as a method of student assessment. Med Teach. 2001;23(6):535‐551. 12098472 10.1080/01421590120090952

[48] France EF Wells M Lang H Williams B . Why, when and how to update a meta-ethnography qualitative synthesis. Syst Rev. 2016;5:44. 26979748 10.1186/s13643-016-0218-4 PMC4791806

[49] France EF Uny I Ring N , et al. A methodological systematic review of meta-ethnography conduct to articulate the complex analytical phases. BMC Med Res Methodol. 2019;19(1):35. 30777031 10.1186/s12874-019-0670-7 PMC6380066

[50] France EF Wells M Lang H Williams B . Why, when and how to update a meta-ethnography qualitative synthesis. Syst Rev. 2016;5(1):1‐12. 26979748 10.1186/s13643-016-0218-4 PMC4791806

[51] France EF Uny I Ring N , et al. A methodological systematic review of meta-ethnography conduct to articulate the complex analytical phases. BMC Med Res Methodol. 2019;19(1):1‐18. 30777031 10.1186/s12874-019-0670-7 PMC6380066

[52] Noblit GW Hare RD Hare RD . Meta-ethnography: Synthesizing qualitative studies. Sage; 1988.

[53] Franco RS dos Santos Franco CAG Pestana O Severo M Ferreira MA . The use of portfolios to foster professionalism: attributes, outcomes, and recommendations. Assessment & Evaluation in Higher Education. 2017;42(5):737‐755.

[54] Driessen E van Tartwijk J Vermunt JD van der Vleuten CP . Use of portfolios in early undergraduate medical training. Med Teach. 2003;25(1):18‐23. 14741854 10.1080/0142159021000061378

[55] Rees CE Shepherd M Chamberlain S . The utility of reflective portfolios as a method of assessing first year medical students’ personal and professional development. Reflective Practice. 2005;6(1):3‐14.

[56] Franco R Ament Giuliani Franco C de Carvalho Filho MA Severo M Amelia Ferreira M . Use of portfolios in teaching communication skills and professionalism for Portuguese-speaking medical students. Int J Med Educ. 2020;11:37‐46. 32061170 10.5116/ijme.5e2a.fa68 PMC7252446

[57] Driessen EW van Tartwijk J Overeem K Vermunt JD van der Vleuten CP . Conditions for successful reflective use of portfolios in undergraduate medical education. Med Educ. 2005;39(12):1230‐1235. 16313582 10.1111/j.1365-2929.2005.02337.x

[58] Avila J Sostmann K Breckwoldt J Peters H . Evaluation of the free, open source software WordPress as electronic portfolio system in undergraduate medical education. BMC Med Educ. 2016;16:157. 27255920 10.1186/s12909-016-0678-1 PMC4891874

[59] Oudkerk Pool A Jaarsma ADC Driessen EW Govaerts MJB . Student perspectives on competency-based portfolios: does a portfolio reflect their competence development? Perspectives on medical education. 2020;9(3):166‐172. 32274650 10.1007/s40037-020-00571-7 PMC7283408

[60] Gordon J . Assessing students’ personal and professional development using portfolios and interviews. Med Educ. 2003;37(4):335‐340. 12654118 10.1046/j.1365-2923.2003.01475.x

[61] Chae SJ Lee YW . Exploring the strategies for successfully building e-portfolios in medical schools. Korean J Med Educ. 2021;33(2):133‐137. 33957730 10.3946/kjme.2021.188 PMC8169370

[62] Yielder J Moir F . Assessing the development of medical Students’ personal and professional skills by portfolio. J Med Educ Curric Dev. 2016;3:JMECD.S30110. 29349315 10.4137/JMECD.S30110 PMC5736276

[63] O'Sullivan AJ Harris P Hughes CS , et al. Linking assessment to undergraduate student capabilities through portfolio examination. Assessment & Evaluation in Higher Education. 2012;37(3):379‐391.

[64] Arntfield S Parlett B Meston CN Apramian T Lingard L . A model of engagement in reflective writing-based portfolios: interactions between points of vulnerability and acts of adaptability. Med Teach. 2016;38(2):196‐205. 25697109 10.3109/0142159X.2015.1009426

[65] Bashook P Gelula M Joshi M Sandlow L . Impact of student reflective e-portfolio on medical student advisors. Teach Learn Med. 2008;20(1):26‐30. 18444182 10.1080/10401330701798113

[66] Belcher R Jones A Smith LJ , et al. Qualitative study of the impact of an authentic electronic portfolio in undergraduate medical education. BMC Med Educ. 2014;14(265). 10.1186/s12909-014-0265-2 PMC4272766 25515320

[67] Chertoff J Wright A Novak M , et al. Status of portfolios in undergraduate medical education in the LCME accredited US medical school Status of portfolios in undergraduate medical education in the LCME accredited US medical school. Med Teach. 2016;38(9):886‐896. 26652913 10.3109/0142159X.2015.1114595

[68] Cotterill S McDonald T Horner P . Using the ePET portfolio to support teaching and learning in Medicine: Lessons from 3 Institutions. 2008.https://s3.eu-west-2.amazonaws.com/assets.creode.advancehe-document-manager/documents/hea/private/using_the_epet_portfolio_1568036930.pdf

[69] Cunningham H Taylor D Desai UA , et al. Looking back to move forward: first-year medical Students’ meta-reflections on their narrative portfolio writings. Acad Med. 2018;93(6):888‐894. 29261540 10.1097/ACM.0000000000002102 PMC5976514

[70] Dannefer EF Bierer SB Gladding SP . Evidence within a portfolio-based assessment program: what do medical students select to document their performance? Med Teach. 2012;34(3):215‐220. 22364453 10.3109/0142159X.2012.652241

[71] Dannefer EF Prayson RA . Supporting students in self-regulation: use of formative feedback and portfolios in a problem-based learning setting. Med Teach. 2013;35(8):655‐660. 23641921 10.3109/0142159X.2013.785630

[72] Dornan T Maredia N Hosie L Lee C Stopford A . A web-based presentation of an undergraduate clinical skills curriculum. Med Educ. 2003;37(6):500‐508. 12787372 10.1046/j.1365-2923.2003.01531.x

[73] Moores A Parks M . Twelve tips for introducing E-portfolios with undergraduate students. Med Teach. 2010;32(1):46‐49. 20095774 10.3109/01421590903434151

[74] Vance GHS Burford B Shapiro E Price R . Longitudinal evaluation of a pilot e-portfolio-based supervision programme for final year medical students: views of students, supervisors and new graduates. BMC Med Educ. 2017;17(1):141. 28830499 10.1186/s12909-017-0981-5 PMC5567902

[75] Babovic M Fu RH Monrouxe LV . Understanding how to enhance efficacy and effectiveness of feedback via e-portfolio: a realist synthesis protocol. BMJ Open. 2019;9(5). 10.1136/bmjopen-2019-029173 PMC6528049 31076477

[76] Carney PA Mejicano GC Bumsted T Quirk M . Assessing learning in the adaptive curriculum. Med Teach. 2018;40(8):813‐819. 30106597 10.1080/0142159X.2018.1484083

[77] Chu A Biancarelli D Drainoni ML , et al. Usability of learning moment: features of an E-learning tool that maximize adoption by students. West J Emerg Med. 2019;21(1):78‐84. 31913823 10.5811/westjem.2019.6.42657 PMC6948698

[78] Désilets V Graillon A Ouellet K Xhignesse M St-Onge C . Reflecting on professional identity in undergraduate medical education: implementation of a novel longitudinal course. Perspectives on medical education. 2021. 10.1007/s40037-021-00649-w PMC9391548 33687729

[79] Heeneman S Driessen E Durning SJ Torre D . Use of an e-portfolio mapping tool: connecting experiences, analysis and action by learners. Perspect Med Educ. 2019;8(3):197‐200. 31098981 10.1007/s40037-019-0514-5 PMC6565639

[80] Kanfi A Faykus MW Tobler J Dallaghan GLB England E Jordan SG . The early bird gets the work: maintaining a longitudinal learner portfolio From medical school to physician practice. Acad Radiol. 2021;S1076-6332(20)30705-4. 10.1016/j.acra.2020.12.012 33413963

[81] Mejicano GC Bumsted TN . Describing the journey and lessons learned implementing a competency-based, time-Variable undergraduate medical education curriculum. Acad Med. 2018;93(3S Competency-Based, Time-Variable Education in the Health Professions):S42‐S48. 29485487 10.1097/ACM.0000000000002068

[82] Roskvist R Eggleton K Goodyear-Smith F . Provision of e-learning programmes to replace undergraduate medical students’ clinical general practice attachments during COVID-19 stand-down. Education for primary care : an official publication of the Association of Course Organisers, National Association of GP Tutors, World Organisation of Family Doctors. 2020;31(4):247‐254. 10.1080/14739879.2020.1772123 32469632

[83] Santonja-Medina F García-Sanz MP Santonja-Renedo S García-Estañ J . Mismatch between student and tutor evaluation of training needs: a study of traumatology rotations. BMC Res Notes. 2018;11(1):826. 30463605 10.1186/s13104-018-3925-1 PMC6249731

[84] Sohrmann M Berendonk C Nendaz M Bonvin R . Swiss Working group For profiles I. Nationwide introduction of a new competency framework for undergraduate medical curricula: a collaborative approach. Swiss Med Wkly. 2020;150:w20201. 32294223 10.57187/smw.2020.20201

[85] Ten Cate O Graafmans L Posthumus I Welink L van Dijk M . The EPA-based Utrecht undergraduate clinical curriculum: development and implementation. Med Teach. 2018;40(5):506‐513. 29468913 10.1080/0142159X.2018.1435856

[86] Byszewski A Fraser A Lochnan H . East meets west: shadow coaching to support online reflective practice. Perspect Med Educ. 2018;7(6):412‐416. 30361984 10.1007/s40037-018-0476-z PMC6283780

[87] O'Sullivan AJ Howe AC Miles S , et al. Does a summative portfolio foster the development of capabilities such as reflective practice and understanding ethics? An evaluation from two medical schools. Med Teach. 2012;34(1):e21‐e28. 22250692 10.3109/0142159X.2012.638009

[88] Mason G Langendyk V Wang S . “The game is in the tutorial”: an evaluation of the use of an e-portfolio for personal and professional development in a medical school. 2014.https://ascilite2014.otago.ac.nz/files/fullpapers/43-Mason.pdf

[89] Haig A Dozier M . BEME Guide no 3: systematic searching for evidence in medical education--part 1: sources of information. Med Teach. 2003;25(4):352‐363. 12893544 10.1080/0142159031000136815

[90] Gordon M Gibbs T . STORIES Statement: publication standards for healthcare education evidence synthesis. BMC Med. 2014;12(1):143. 25190085 10.1186/s12916-014-0143-0 PMC4243720

[91] Davis MH Ponnamperuma GG Ker JS . Student perceptions of a portfolio assessment process. Med Educ. 2009;43(1):89‐98. 19141002 10.1111/j.1365-2923.2008.03250.x

[92] Ho CY Kow CS Chia CHJ , et al. The impact of death and dying on the personhood of medical students: a systematic scoping review. BMC Med Educ. 2020;20(1):516. 33371878 10.1186/s12909-020-02411-y PMC7768997

[93] Kuek JTY Ngiam LXL Kamal NHA , et al. The impact of caring for dying patients in intensive care units on a physician's personhood: a systematic scoping review. Philos Ethics Humanit Med. 2020;15(1):12. 33234133 10.1186/s13010-020-00096-1 PMC7685911

[94] Ten Cate O Taylor DR . The recommended description of an entrustable professional activity: AMEE Guide No. 140. Med Teach. 2021;43(10):1106–1114. 33167763 10.1080/0142159X.2020.1838465

[95] Ten Cate O . AM Last page: what entrustable professional activities add to a competency- based curriculum. Acad Med. 2014;89(4):691. 24667478 10.1097/ACM.0000000000000161 PMC4885550

[96] Carraccio C Englander R Gilhooly J , et al. Building a framework of entrustable professional activities, supported by competencies and milestones, to bridge the educational Continuum. Acad Med. 2017;92(3):324‐330. 26959225 10.1097/ACM.0000000000001141

[97] Pinilla S Lenouvel E Cantisani A . Working with entrustable professional activities in clinical education in undergraduate medical education: a scoping review. BMC Med Educ. 2021;21(172). 10.1186/s12909-021-02608-9 PMC7980680 33740970

[98] Hong DZ Lim AJS Tan R , et al. A systematic scoping review on portfolios of medical educators. Journal of Medical Education and Curricular Development. 2021. 10.1177/23821205211000356 PMC8855455 35187262

[99] Zhou YC Tan SR Tan CGH , et al. A systematic scoping review of approaches to teaching and assessing empathy in medicine. BMC Med Educ. 2021;21(292). 10.1186/s12909-021-02697-6 PMC8140468 34020647

[100] Norcini J . Is it time for a new model of education in the health professions? Med Educ. 2020;54(8):687‐690. 31860934 10.1111/medu.14036

[101] Beck Dallaghan GL Coplit L Cutrer WB Crow S . Medical student portfolios: their value and what You need for successful implementation. Acad Med. 2020;95(9):1457. 32349017 10.1097/ACM.0000000000003456

[102] Datta R Datta K Routh D , et al. Development of a portfolio framework for implementation of an outcomes-based healthcare professional education curriculum using a modified e-delphi method. Medical Journal Armed Forces India. 2021;77:S49‐S56. 10.1016/j.mjafi.2020.11.012 PMC7873694 33612932

[103] Cunningham H Taylor DS Desai UA , et al. Reading the self: medical Students’ experience of reflecting on their writing over time. Acad Med. 2020. 10.1097/ACM.0000000000003814 33149084

[104] Ross S Maclachlan A Cleland J . Students’ attitudes towards the introduction of a personal and professional development portfolio: potential barriers and facilitators. BMC Med Educ. 2009;9(69). 10.1186/1472-6920-9-69 PMC2790436 19951406

[105] Bierer SB Dannefer E . Does Students’ gender, citizenship, or verbal ability affect fairness of portfolio-based promotion decisions? Results From One medical school. Acad Med. 2011;86(6):773‐777. 21512368 10.1097/ACM.0b013e318217e14b

[106] Rees C Sheard C . Undergraduate medical students’ views about a reflective portfolio assessment of their communication skills learning. Med Educ. 2004;38(2):125‐128. 14871382 10.1111/j.1365-2923.2004.01750.x

[107] Hall P Byszewski A Sutherland S Stodel EJ . Developing a sustainable electronic portfolio (ePortfolio) program that fosters reflective practice and incorporates CanMEDS competencies into the undergraduate medical curriculum. Acad Med. 2012;87(6):744‐751. 22534601 10.1097/ACM.0b013e318253dacd

[108] Ekayanti F Risahmawati R Fadhilah M . Portfolio Assessment Implementation in Clinical Year of Community Medicine Module: Students’ Perspective. Adv Health Sci Educ Theory Pract. 2017;10:44–48.

[109] Sheng AY Chu A Biancarelli D Drainoni ML Sullivan R Schneider JI . A novel Web-based experiential learning platform for medical students (learning moment): qualitative study. JMIR Med Educ. 2018;4(2):e10657. 30333094 10.2196/10657 PMC6231881

[110] van Schaik S Plant J O'Sullivan P . Promoting self-directed learning through portfolios in undergraduate medical education: the mentors’ perspective. Med Teach. 2013;35(2):139‐144. 23102105 10.3109/0142159X.2012.733832

[111] O'Brien CL Sanguino SM Thomas JX Green MM . Feasibility and outcomes of implementing a portfolio assessment system alongside a traditional grading system. Acad Med. 2016;91(11):1554‐1560. 27028027 10.1097/ACM.0000000000001168

[112] Michels NR Driessen EW Muijtjens AM Van Gaal LF Bossaert LL De Winter BY . Portfolio assessment during medical internships: how to obtain a reliable and feasible assessment procedure. Educ Health (Abingdon). 2009;22[3]:313. 20029764

[113] Borgstrom E Cohn S Barclay S . Medical professionalism: conflicting values for tomorrow's doctors. J Gen Intern Med. 2010;25(12):1330‐1336. 20740324 10.1007/s11606-010-1485-8 PMC2988149

[114] Chaffey LJ de Leeuw EJ Finnigan GA . Facilitating students’ reflective practice in a medical course: literature review. Educ Health (Abingdon). 2012;25[3]:198‐203. 23823640 10.4103/1357-6283.109787

[115] Deketelaere A Kelchtermans G Druine N Vandermeersch E Struyf E De Leyn P . Making more of it! medical students’ motives for voluntarily keeping an extended portfolio. Med Teach. 2007;29(8):798‐805. 18236275 10.1080/01421590701477340

[116] Haffling AC Beckman A Pahlmblad A Edgren G . Students’ reflections in a portfolio pilot: highlighting professional issues. Med Teach. 2010;32(12):e532‐e540. 21090940 10.3109/0142159X.2010.509420

[117] Rees CE Sheard CE . The reliability of assessment criteria for undergraduate medical students’ communication skills portfolios: the nottingham experience. Med Educ. 2004;38(2):138‐144. 14871384 10.1111/j.1365-2923.2004.01744.x

[118] Cherfi Y Szántó K . Student portfolios: not just a tick-box exercise. Clin Teach. 2019;16(6):641‐642. 30506872 10.1111/tct.12974

[119] Forenc KM Eriksson FM Malhotra B . Medical Students’ perspectives on an assessment of reflective portfolios. Adv Med Educ Pract. 2020;11:463‐464. 32669885 10.2147/AMEP.S266849 PMC7335843

[120] Imafuku R Saiki T Hayakawa K Sakashita K Suzuki Y . Rewarding journeys: exploring medical students’ learning experiences in international electives. Med Educ Online. 2021;26(1):1913784. 33829969 10.1080/10872981.2021.1913784 PMC8043609

[121] Kassab SE Bidmos M Nomikos M , et al. Construct validity of an instrument for assessment of reflective writing-based portfolios of medical students. Adv Med Educ Pract. 2020;11:397‐404. 32581621 10.2147/AMEP.S256338 PMC7276316

[122] Kim JW Ryu H Park JB , et al. Establishing a patient-centered longitudinal integrated clerkship: early results from a single institution. J Korean Med Sci. 2020;35(50):e419. 33372421 10.3346/jkms.2020.35.e419 PMC7769701

[123] Yoo DM Cho AR Kim S . Evaluation of a portfolio-based course on self-development for pre-medical students in korea. J Educ Eval Health Prof. 2019;16:38. 32299188 10.3352/jeehp.2019.16.38 PMC7040426

[124] Yoo DM Cho AR Kim S . Development and validation of a portfolio assessment system for medical schools in korea. J Educ Eval Health Prof. 2020;17:39. 33291206 10.3352/jeehp.2020.17.39 PMC7859386

[125] Van Tartwijk J Driessen EW . Portfolios for assessment and learning: AMEE. Guide no. 45. Med Teach. 2009;31(9):790‐801. 19811183 10.1080/01421590903139201

[126] Sahu SK Soudarssanane M Roy G Premrajan K Sarkar S . Use of portfolio-based learning and assessment in community-based field curriculum. Indian J Community Med. 2008;33(2):81‐84. 19967029 10.4103/0970-0218.40873 PMC2784631

[127] Prayson RA Bierer SB Dannefer EF . Medical student resilience strategies: a content analysis of medical students’ portfolios. Perspect Med Educ. 2017;6(1):29‐35. 27957671 10.1007/s40037-016-0313-1 PMC5285273

[128] Özçakar N Mevsim V Güldal D . Use of portfolios in undergraduate medical training: first meeting With a patient. Balkan Med J. 2009;26(2):145‐150.

[129] Austin C Braidman I . Support for portfolio in the initial years of the undergraduate medical school curriculum: what do the tutors think? Med Teach. 2008;30(3):265‐271. 18484453 10.1080/01421590701758673

[130] King TS Sharma R Jackson J Fiebelkorn KR . Clinical case-based image portfolios in medical histopathology. Anat Sci Educ. 2019;12(2):200‐209. 30118571 10.1002/ase.1794

[131] Michels NR Avonts M Peeraer G , et al. Content validity of workplace-based portfolios: a multi-centre study. Med Teach. 2016;38(9):936‐945. 26829024 10.3109/0142159X.2015.1132407

[132] Montrezor LH . Lectures and collaborative working improves the performance of medical students. Adv Physiol Educ. 2021;45(1):18‐23. 33439785 10.1152/advan.00121.2020

[133] Zundel S Blumenstock G Zipfel S Herrmann-Werner A Holderried F . Portfolios enhance clinical activity in surgical clerks. J Surg Educ. 2015;72(5):927‐935. 26002535 10.1016/j.jsurg.2015.03.014

[134] Dolan BM O'Brien CL Cameron KA Green MM . A qualitative analysis of narrative preclerkship assessment data to evaluate teamwork skills. Teach Learn Med. 2018;30(4):395‐403. 29658802 10.1080/10401334.2018.1450146

[135] Roberts C Shadbolt N Clark T Simpson P . The reliability and validity of a portfolio designed as a programmatic assessment of performance in an integrated clinical placement. BMC Med Educ. 2014;14:197. 25240385 10.1186/1472-6920-14-197 PMC4182797

[136] Adeleke OA Cawe B Yogeswaran P . Opportunity for change: undergraduate training in family medicine. S Afr Fam Pract (2004). 2020;62(1):1‐3. 10.4102/safp.v62i1.5225 PMC8378155 33314949

[137] Brits H Bezuidenhout J Van der Merwe LJ . Quality assessment in undergraduate medical training: how to bridge the gap between what we do and what we should do. Pan African Medical Journal. 2020;36(79). 10.11604/pamj.2020.36.79.23658 PMC7386270 32774638

[138] Pinto-Powell R Lahey T . Just a game: the dangers of quantifying medical student professionalism. J Gen Intern Med. 2019;34(8):1641‐1644. 31147979 10.1007/s11606-019-05063-x PMC6667566

[139] Bierer SB Dannefer EF Tetzlaff JE . Time to loosen the apron strings: cohort-based evaluation of a learner-driven remediation model at One medical school. J Gen Intern Med. 2015;30(9):1339‐1343. 26173525 10.1007/s11606-015-3343-1 PMC4539324

[140] Davis MH Ponnamperuma GG . Examiner perceptions of a portfolio assessment process. Med Teach. 2010;32(5):e211‐e215. 20423247 10.3109/01421591003690312

[141] Shiozawa T Glauben M Banzhaf M , et al. An insight into professional identity formation: qualitative analyses of Two reflection interventions during the dissection course. Anat Sci Educ. 2020;13(3):320‐332. 31509334 10.1002/ase.1917

[142] O'Brien C Thomas J Green M . What is the relationship between a preclerkship portfolio review and later performance in clerkships? Acad Med. 2017;93(1):113–118. 10.1097/ACM.0000000000001771 28640026

[143] Kassab SE Bidmos M Nomikos M , et al. Medical Students’ perspectives on an assessment of reflective portfolios [response to letter]. Adv Med Educ Pract. 2020;11:495‐496. 32765153 10.2147/AMEP.S270581 PMC7381774

[144] Pitkälä KH Mäntyranta T . Feelings related to first patient experiences in medical school A qualitative study on students’ personal portfolios. Pt Educ Couns. 2004;54(2):171‐177. 10.1016/S0738-3991(03)00209-X 15288911

[145] Royce CS Everett EN Craig LB , et al. To the point: advising students applying to obstetrics and gynecology residency in 2020 and beyond. Am J Obstet Gynecol. 2021;224(2):148‐157. 33038302 10.1016/j.ajog.2020.10.006 PMC7539929

[146] Goldie J Dowie A Cotton P Morrison J . Teaching professionalism in the early years of a medical curriculum: a qualitative study. Med Educ. 2007;41(6):610‐617. 17518842 10.1111/j.1365-2923.2007.02772.x

[147] Duque G Finkelstein A Roberts A Tabatabai D Gold S Winer L . Members of the division of geriatric medicine MU learning while evaluating: the use of an electronic evaluation portfolio in a geriatric medicine clerkship. BMC Med Educ. 2006;6:1‐7. 16409640 10.1186/1472-6920-6-4 PMC1361794

[148] Sturmberg JP Farmer L . Educating capable doctors-A portfolio approach. Linking learning and assessment. Med Teach. 2009;31(3):e85‐e89. 19089726 10.1080/01421590802512912

[149] Souza AD Vaswani V . Diversity in approach to teaching and assessing ethics education for medical undergraduates: a scoping review. Annals of Medicine and Surgery. 2020;56:178‐185. 32642060 10.1016/j.amsu.2020.06.028 PMC7334795

[150] Amin TT Kaliyadan F Al-Muhaidib NS . Medical students’ assessment preferences at king faisal university, Saudi Arabia. Adv Med Educ Pract. 2011;2:95‐103. 23745080 10.2147/AMEP.S12950 PMC3661244

[151] Kennedy G Rea JNM Rea IM . Prompting medical students to self-assess their learning needs during the ageing and health module: a mixed methods study. Med Educ Online. 2019;24(1):1579558. 31046637 10.1080/10872981.2019.1579558 PMC6508056

[152] Driessen E van Tartwijk J Dornan T . The self critical doctor: helping students become more reflective. Br Med J. 2008;336(7648):827‐830. 18403547 10.1136/bmj.39503.608032.AD PMC2292362

