# Supplementary material for: Medical Student Portfolios: A Systematic Scoping Review
Source: J Med Educ Curric Dev. 2022 Mar 3;9:23821205221076022. doi: 10.1177/23821205221076022 (PMC8902199; doi:10.1177/23821205221076022)
Supplement: Supplementary material [file sj-pdf-1-mde-10.1177_23821205221076022.pdf]

Supplementary File 1 – PubMed Search Strategy

Search Strategy A: search on six bibliographic databases

		Mesh Terms	tiab
Population	Medical Students OR Doctors	[1] “Physicians”[MeSH] OR “Students, Medical”[MeSH] OR “Clinical Clerkship”[MeSH] OR "Medicine"[Mesh] OR "Education, Medical"[Mesh] OR "Clinical Competence”[Mesh]	[2] Physician[tiab] OR Physicians[tiab] OR resident[tiab] OR residents[tiab] OR residency[tiab] OR residencies[tiab] OR practice[tiab] OR practitioner[tiab] OR practitioners[tiab] OR doctor[tiab] OR doctors[tiab] OR houseman[tiab] OR housemanship[tiab] OR housemen[tiab] OR medical[tiab] OR clinical[tiab] OR pre-clinical[tiab] OR preclinical[tiab] OR clinician*[tiab] OR surgery[tiab] OR surgical[tiab] OR surgeon*[tiab] OR clerkship*[tiab] OR specialist*[tiab]
Context			[3] ("Educational Measurement/methods"[Mesh] OR “Educational Measurement/standards"[Mesh] OR "Documentation/methods"[Mesh] OR “Benchmarking*”[MeSH] OR “Competency-based education/standards*”[MeSH] OR “Records*”[MeSH]) AND (medical[tiab] OR clinical[tiab] OR pre-clinical[tiab] OR preclinical[tiab] OR clinician*[tiab] OR surgery[tiab] OR surgical[tiab] OR surgeon*[tiab] OR clerkship*[tiab] OR specialist*[tiab])
Intervention	Portfolios		[4] Portfolio[tiab] OR portfolios[tiab] OR e-portfolio[tiab] OR e-portfolios[tiab] OR “curriculum vitae”[tiab] OR “personal statement”[tiab] OR “personal statements”[tiab]

Supplementary File 1 – PubMed Search Strategy

(1 OR 2 OR 3) AND 4

Search Strategy B: search on for 50 relevant articles on pubmed

"Education, Medical, Undergraduate"[Mesh] AND "Portfolio"[tiab]

Filter: full-text, 2000-2020

Supplementary File 1 – Tabulated Summaries of Included Articles

No	Author	Year	Title	MERSQI	COREQ	Background	Theoretical Approach and Methods	Main Empirical Findings	Insights Drawn
1	Dornan, T.<br>Maredia, N.<br>Hosie, L.<br>Lee, C.<br>Stopford, A.	2003	A web-based presentation of an undergraduate clinical skills curriculum	7	17	The aim of this study was to use information and communications technology to present a curriculum of clinical skills in a user-friendly format. It took place in a UK undergraduate medical school with a problem-based curriculum and a strong emphasis on proficiency in clinical skills.	Case study describing the qualitative analysis of users' requirements and development of a web-based learning portfolio. The study involved direct observation of users during a 'think-aloud' protocol, a validated software users' measurement inventory and a 17-item questionnaire designed to test whether 'SkillsBase' met its users' requirements.	Students wanted a clear and flexible presentation of their skills curriculum that was easy to navigate, offered instructional material and standards for self and peer assessment, offered useful Internet links, allowed them to compare their progress with school standards and peer norms, and could be used as a learning portfolio. During the think-aloud protocol, students made very few errors in data interpretation or navigation, and found SkillsBase easy to learn and aesthetically pleasing to use. They rated it higher on all measures of usability than standard commercial software. The questionnaire showed that it met most aspects of its design specification, although many students were doubtful that they would use its reflective function. It is available for inspection at http:// www.skillsbase.man.ac.uk/.	SkillsBase meets the design specification for a training and reflective aid to learning clinical skills and is very usable.
2	Driessen, Erik<br>Van Tartwijk, Jan<br>Vermunt, Jan<br>van der Vleuten, Cees	2003	Use of portfolios in early undergraduate medical training	-	10	The ability to reflect on one's own action is seen as an important skill for a doctor. A thorough introduction of the portfolio planned in the early stages of their studies seems to be the way	During the academic year 2001–02 242 first-year medical students compiled a portfolio. Student experience was collected by semi-structured interviews.	The majority of students were of the opinion that analysing one's competences in a portfolio was instructive and meaningful. With regard to learning how to reflect and recognize learning needs	The results thus far show that the portfolio is a worthwhile addition to existing assessment and learning tools.

						to train medical students in reflection. This article describes the use of portfolios in early undergraduate medical training. The literature on portfolios suggests three aspects that are crucial for the effectiveness of portfolios: structure, coaching and assessment. The portfolio system was designed by transposing the experience with portfolio systems outside and inside medical training to a situation of first year medical students.		however, mentor coaching proved to be necessary.	
3	Driessen, E. W.<br>Overeem, K.<br>van Tartwijk, J.<br>van der Vleuten, C. P.<br>Muijtjens, A. M.	2006	Validity of portfolio assessment: which qualities determine ratings?	7.5	-	The portfolio is becoming increasingly accepted as a valuable tool for learning and assessment. The validity of portfolio assessment, however, may suffer from bias due to irrelevant qualities, such as lay-out and writing style. We examined the possible effects of such qualities in a portfolio programme aimed at stimulating Year 1 medical students to reflect on	We developed an instrument, the Portfolio Analysis Scoring Inventory, to examine the impact of form and content aspects on portfolio assessment. The Inventory consists of 15 items derived from interviews with experienced mentors, the literature, and the criteria for reflective competence used in the regular portfolio assessment procedure. Forty portfolios, selected from 231 portfolios for which ratings from the regular assessment procedure were available, were rated by 2 researchers, independently, using the Inventory. Regression analysis was used to estimate	Inter-rater agreement ranged from 0.46 to 0.87. The strongest predictor of the variance in the regular ratings was quality of reflection (R 0.80;<br>R2 66%). No further items accounted for a significant proportion of variance. Irrelevant items, such as writing style and lay-out, had negligible effects.	The absence of an impact of irrelevant criteria appears to support the validity of the portfolio assessment procedure. Further studies should examine the portfolio's validity for the assessment of clinical competence.

						their professional and personal development. In later curricular years, this portfolio is also used to judge clinical competence.	the correlation between the ratings from the regular assessment and those resulting from the Inventory items.		
4	Driessen, E. W. van Tartwijk, J. Overeem, K. Vermunt, J. D. van der Vleuten, C. P.	2005	Conditions for successful reflective use of portfolios in undergraduate medical education	-	18	Portfolios are often used as an instrument with which to stimulate students to reflect on their experiences. Research has shown that working with portfolios does not automatically stimulate reflection. In this study we addressed the question: What are the conditions for successful reflective use of portfolios in undergraduate medical education?	We designed a portfolio that was aimed at stimulating reflection in early undergraduate medical education, using experiences described in the medical education literature and elsewhere. Conditions for reflective portfolio use were identified through interviews with 13 teachers (mentors), who were experienced in mentoring students in the process of developing their portfolios. The interviews were analysed according to the principles of grounded theory.	The conditions for successful reflective use of portfolios that emerged from the interviews fell into 4 categories: coaching; portfolio structure and guidelines; relevant experiences and materials, and summative assessment. According to the mentors, working with a portfolio designed to meet these conditions will stimulate students' reflective abilities.	This study shows that portfolios are a potentially valuable method of assessing and developing students' reflective skills in undergraduate medical training, provided certain conditions for effective portfolios are recognised and met. Portfolios have a strong potential for enhancing learning and assessment but they are very vulnerable and may easily lead to disappointment. Before implementing portfolios in education, one should first consider whether the necessary conditions can be fulfilled, including an appropriate portfolio structure,

									an appropriate assessment procedure, the provision of enough new experiences and materials, and sufficient teacher capacity for adequate coaching and assessment.
5	Duque, G<br>Finkelstein, A<br>Roberts, A<br>Tabatabai, D<br>Gold, SL<br>Winer, LR	2006	Learning while evaluating: The use of an electronic evaluation portfolio in a geriatric medicine clerkship	9.5	-	Electronic evaluation portfolios may play a role in learning and evaluation in clinical settings and may complement other traditional evaluation methods (bedside evaluations, written exams and tutor-led evaluations).	133 third-year medical students used the McGill Electronic Evaluation Portfolio (MEEP) during their one-month clerkship rotation in Geriatric Medicine between September 2002 and September 2003. Students were divided into two groups, one who received an introductory hands-on session about the electronic evaluation portfolio and one who did not. Students' marks in their portfolios were compared between both groups. Additionally, students self-evaluated their performance and received feedback using the electronic portfolio during their mandatory clerkship rotation. Students were surveyed immediately after the rotation and at the end of the clerkship year. Tutors' opinions about this method were surveyed once. Finally, the number of evaluations/ month was quantified. In all surveys, Likert scales were used and were analyzed using Chi-square tests and t-tests to assess significant	The introductory session had a significant effect on students' portfolio marks as well as on their comfort using the system. Both tutors and students reported positive notions about the method. Remarkably, an average (± SD) of 520 (± 70) evaluations/month was recorded with 30 (± 5) evaluations per student/month.	The MEEP showed a significant and positive effect on both students' self-evaluations and tutors' evaluations involving an important amount of self-reflection and feedback which may complement the more traditional evaluation methods.

							differences in the responses from surveyed subjects.		
6	Ekayanti, Fika Risahmawati, Risahmawati Fadhilah, Marita	2017	Portfolio Assessment Implementation in Clinical Year of Community Medicine Module: Students Perspective	7	-	Portfolio has been used as summative assessment in many fields of study. Since 2010, clinical community medicine module has used portfolio to assess students. Nine portfolios were assigned to students within 5 weeks length module. This study aimed to identify the correlation of students' perspective for using portfolio and their portfolio scores.	The data was collected by questionnaires from 46 students after completing module in November 2016. Students were asked about their satisfaction of using portfolio, then correlated to their portfolio scores. The reasons and suggestion for ideal portfolios were identified. Analysis was done by SPSS 20 using Rank Spearman correlation test.	Students felt that creating portfolios were not comfortable. Most students (36/78.3%) felt that the portfolio was not appropriate as their summative assessment. They preferred less portfolios, 30 (63%) students requested to decrease portfolios to 3-5, 28.3% to 6-8 and only 8.7% to <3. There was no significant correlation between students' satisfaction of using portfolio to their portfolios score (p=0.262), while there was significant correlation between students' score to the number of portfolios to be submitted (p=0.017; r=0.349). Reasons for decreasing number of portfolios were inadequate time and many other tasks to be finished. Students in UIN Jakarta haven't used to retelling their experiences and reflecting them in writing as perceived in many students who were not used to portfolios. Doing portfolios need lots of work and time-consuming. Having lesser number of portfolios to be submitted would encourage students to create portfolios. Building familiarization, time management and good motivation for creating	-

								portfolios were important to successful portfolios. There was no significant correlation between students' satisfaction to their scores. Students should be encouraged to get comfortable in creating portfolios for the benefit as lifelong learning tool. Motivation to write and reflect should be nurtured to improve students' portfolio satisfaction and commitment.	
7	Fida, N. M.<br>Hassanien, M.<br>Shamim, M. S.<br>Alafari, R.<br>Zaini, R.<br>Mufti, S.<br>Al-Hayani, A.<br>Farouq, M.<br>Al-Zahrani, H.	2018	Students' perception of portfolio as a learning tool at King Abdulaziz University Medical School	8	14	Medical education has a longstanding tradition of using logbooks to record activities. The portfolio is an alternative tool to document competence and promote reflective practice. This study assessed the acceptance of portfolio use among Saudi undergraduate medical students.	Portfolios were introduced in the 2nd through 5th years at King Abdulaziz University over a two-year period (2013–2015). At the end of each academic year, students completed a mixed questionnaire that included a self-assessment of skills learned through the use of portfolio.	The results showed a difference in focus between basic and clinical years: in basic years students' focus was on acquiring practical skills, but in clinical years they focused more on acquiring complex skills, including identifying and managing problems. The questionnaire responses nonetheless revealed a positive trend in acceptance (belief in the educational value) of portfolios among students and their mentors, across the years of the program.	Using portfolios as a developmental learning and formative assessment tool in the early undergraduate years was found to contribute to students' ability to create their own clinical skills guidelines in later years, as well as to engage in and appreciate reflective learning.
8	Renato Soleiman Franco, Camila Ament Giuliani dos Santos Franco,Olívia Pestana,Milton Severo &Maria Amélia Ferreira	2017	The use of portfolios to foster professionalism: Attributes, outcomes, and recommendations	-	9	The main objective of this research was to review the characteristics of portfolios and their outcomes for teaching professionalism to undergraduate medical students.	A systematic review on the use of portfolios in teaching professionalism to medical students identified 1257 papers. Of these, 11 articles met all inclusion criteria.	According to the papers, the use of portfolios for teaching professionalism shows versatility, supports learning strategies and has the potential to be used in different contexts, including for formative and summative purposes. The weaknesses	A framework was designed to support faculty members in developing and applying portfolios with a clear and broad view of this teaching strategy.

								were based on the artificiality of the reflections, deficient instructions, time-consuming processes and preference among students for other teaching methods. Students complained about feeling that the reflection was 'forced', and they tended to write based on social conventions rather than reveal their true thoughts. Reflection is a powerful component of the portfolio, but the method by which it is taught could easily ruin its potential to boost professionalism. Requiring re ection did not ensure its achievement; increased understanding by students regarding how and why they were doing it, the clarity of assessment methods and constructive feedback might strengthen the potential for success.	
9	Friedman Ben David, M.<br>Davis, M. H.<br>Harden, R. M.<br>Howie, P. W.<br>Ker, J.<br>Pippard, M. J.	2001	AMEE Medical Education Guide No. 24: Portfolios as a method of student assessment	-	-	This guide is intended to inform medical teachers about the use of portfolios for student assessment. It provides a background to the topic, reviews the range of assessment purposes for which portfolios have been used, identifies possible portfolio contents and outlines the advantages of	-	-	The final part of the paper provides a practical guide for those wishing to design and implement portfolio assessment in their own institutions. Five steps in the portfolio assessment process are identified:

						portfolio assessment with particular focus on assessing professionalism. The experience of one medical school, the University of Dundee, is presented as a case study. The current state of understanding of the technical, psychometric issues relating to portfolio assessment is clarified.			documentation, reflection, evaluation, defence and decision. It is concluded that portfolio assessment is an important addition to the assessor's toolkit. Reasons for using portfolios for assessment purposes include the impact that they have in driving student learning and their ability to measure outcomes such as professionalism that are difficult to assess using traditional methods.
10	Goldie, J.<br>Dowie, A.<br>Cotton, P.<br>Morrison, J.	2007	Teaching professionalism in the early years of a medical curriculum: a qualitative study	-	18	Despite the growing literature on professionalism in undergraduate medical curricula, few studies have examined its delivery. This study investigated tutors' and students' perspectives of the delivery of professionalism in the early years of Glasgow's learner-centred, problem-based learning (PBL),	A qualitative approach was adopted involving semistructured interviews, on a 1 in 6 sample of tutors involved in teaching in the early curricular years, and 3 student focus groups. The findings were subjected to between-method triangulation.	Involvement in teaching raised students' and tutors' awareness of their professionalism. Learning activities promoting critical reflection were most effective. The integration of professionalism across the domains of Vocational Studies (VS) was important for learning; however, it was not well integrated with the PBL core. Integration was promoted by having the same tutor present throughout all VS sessions. Early patient contact	Reflection is integral to professional development. Early clinical contact is an important part of the process of socialisation, as it allows students to enter the community of practice that is the medical profession. Role models can contribute

						integrated medical curriculum.		experiences were found to be particularly important. The hidden curriculum provided both opportunities for, and threats to, learning. The small-group format provided a suitable environment for the examination of pre-existing perspectives. The portfolio was an effective learning tool, although its assessment should be formalised.	powerfully to students' learning and identity formation. As students move towards fuller participation, the clinical milieu should be controlled to maximise the influence of role models, and opportunities for guided reflection should be sustained.
11	Gordon, J.	2003	Assessing students' personal and professional development using portfolios and interviews	6.5	-	Medical schools are placing more emphasis on students' personal and professional development (PPD) and are seeking ways of assessing student progress towards meeting outcome goals in relation to professionalism. The Faculty of Medicine at the University of Sydney sought an assessment method that would demonstrate the value of reflection in attaining PPD, provide feedback and encourage students to take responsibility for setting and achieving	The instruments used to assess Year 1 students in PPD are a portfolio and interview. This assessment format encourages students to explore ideas and values that are important to them and relevant to the PPD theme. A confidential interview, based on the PPD goals, is held with a faculty member who has read the student's portfolio.	In 1997/98, 96% of students agreed that they had engaged in useful reflection on their approach to the course and 91% agreed that the experience was worthwhile. A further 76% of students agreed that they could see opportunities to modify their approach in some ways as result of this exercise.	Sustained PPD is essential in equipping doctors for the varied stresses of careers in medicine. Despite, or perhaps because of, the latitude in the Year 1 assessment, both students and faculty members found the process of value. This form of assessment acknowledges that the most valid assessment formats cannot always be made reliable and that in some parts of the curriculum it is

						high standards of performance.			more important to demonstrate trust in students' own motivation to become competent and mindful practitioners. The fact that the portfolio and interview are the only summative assessments in the first year emphasises the importance that the Faculty places on PPD.
12	Rees, Charlotte E.<br>Shepherd, Maggie<br>Chamberlain, Suzanne	2005	The utility of reflective portfolios as a method of assessing first year medical students' personal and professional development	8	17	This study explores the reliability, validity and acceptability of assessment criteria for reflective portfolios at Peninsula Medical School, UK.	The construct validity of the criteria was established by exploring its relationship with two other assessment methods and its reliability was determined using the generalizability (G) coefficient. Focus groups with assessors and students were convened to explore their views of the portfolios. Two portfolio analyses had a G coefficient of .42. Performance in the portfolio analyses was correlated with performance in personal and professional development judgements ($r = -.512$, $p < .01$) and scientific reports ($r = .273$, $p = .002$).	Themes emerging from the focus groups include students preferring the structured nature of the portfolios but assessors feeling that this reduced the uniqueness of the portfolios. Although students understood the importance of reflective practice, some disliked the process of reflection, particularly reflective writing.	Educators should design assessment criteria that maximize reliability, validity and acceptability rather than simply focusing on reliability alone.
13	Sahu, S. K.<br>Soudarssanane, M.<br>Roy, G.<br>Premrajan, K.<br>Sarkar, S.	2008	Use of Portfolio-based Learning and Assessment in Community-based Field Curriculum	7	-	Portfolio-based learning is recognized in medical education. It helps students to assess themselves as per the key learning	-	The results of this study shows that involving students in framing objectives, developing a mechanism for self-introspection and self-	-

						objectives and outcomes expected out of them. The faculty could also get feedback regarding individual student's progress toward learning outcomes and facilitate the students achieve the same. This article addresses the process of portfolio development and assesses from students feedbacks, if portfolio-based learning is an improvement over record-based study in community-based field studies.		assessment by the students and a mechanism by which faculty can monitor each student's progress toward the defined objectives can significantly enhance the learnability of the students.	
14	Sheng, A. Y.<br>Chu, A.<br>Biancarelli, D.<br>Drainoni, M. L.<br>Sullivan, R.<br>Schneider, J. I.	2018	A Novel Web-Based Experiential Learning Platform for Medical Students (Learning Moment): Qualitative Study	-	22	Experiential learning plays a critical role in learner development. Kolb's 4-part experiential learning model consists of concrete experience, reflective observation, abstract conceptualization, and active experimentation in a recurring cycle. Most clinical environments provide opportunities for experiences and active experimentation but rarely offer structured means for reflection and abstract	We employed purposive sampling to recruit medical students who used Learning Moment during their rotation. We conducted 13 semistructured interviews (10 individual interviews and one 3-person group interview) between January and March 2017 using an ethnographic approach and utilized a general inductive method to analyze and code for potential themes.	A total of 13 students (five in their third year of medical school and eight in their fourth year) voluntarily participated in our qualitative interviews. Five of the 13 (38%) students intended to pursue emergency medicine as their chosen field of specialty. The median number of "learning moments" logged by these students is 6. From our analysis, three key themes emerged relating to the perceived impact of Learning Moment on student learning: (1) logging "learning moments" enhanced memorization, (2)	Learning Moment was successfully implemented into the educational infrastructure in our department. Students identified three mechanisms by which the application optimizes experiential learning, including enabling the logging of "learning moments" to promote memorization, encouraging

						conceptualization that are crucial for learners to learn through experience. We created Learning Moment, a novel Web-based educational tool that integrates principles of asynchronous learning and learning portfolios to fulfill the reflection and abstract conceptualization aspects of Kolb's learning cycle in the modern clinical learning environment. Medical students log concise clinical "pearls" in the form of "learning moments" for reflection, review, and sharing with peers in a community of practice. We sought to evaluate learners' experiences with Learning Moment via a qualitative study.		improved learning through reflection, and (3) sharing of knowledge and experiences in a community of practice.	reflection to facilitate learning, and fostering the sharing of knowledge and experiences within a community of practice. The Learning Moment concept is potentially scalable to other departments, disciplines, and institutions as we seek to optimize experiential learning ecosystems for all trainees.
15	van Schaik, S. Plant, J. O'Sullivan, P.	2013	Promoting self-directed learning through portfolios in undergraduate medical education: the mentors' perspective	-	17	Medical students need to acquire self-directed learning (SDL) skills for effective lifelong learning. Portfolios allow learners to reflect on their progress, diagnose learning needs and create learning plans, all elements of SDL. While mentorship is	Interviews with faculty members who mentored medical students in portfolio were audio-recorded, transcribed and analysed for themes.	Eight mentors participated. Qualitative analysis revealed six major themes around mentors' definitions of SDL, their perception of innate SDL abilities of medical students, their own approach to SDL, their understanding of the value of learning plans, their perceptions of students' engagement with the portfolio and the impact of	This study revealed tensions between mentors' beliefs regarding the importance of SDL, their own approach to SDL and their perceptions of students' SDL skills. Based on our analysis of these tensions, we

						deemed to be essential for successful portfolio use, it is not known what constitutes effective mentorship in this process. In-depth understanding of the SDL construct seems a prerequisite. The aim of this study was to examine how portfolio mentors perceive and approach SDL.		the portfolio process on the mentoring relationship.	recommend both explicit faculty development and institutional culture change for successful integration of SDL in medical education.
16	Wong, A. Trollope-Kumar, K.	2014	Reflections: an inquiry into medical students' professional identity formation	-	14	Professional identity formation plays a crucial role in the transition from medical student to doctor. At McMaster University, medical students maintain a portfolio of narrative reflections of their experiences, which provides for a rich source of data into their professional development. The purpose of this study was to understand the major influences on medical students' professional identity formation.	Sixty-five medical students (46 women; 19 men) from a class of 194 consented to the study of their portfolios. In total, 604 reflections were analysed and coded using thematic narrative analysis. The codes were merged under subthemes and themes. Common or recurrent themes were identified in order to develop a descriptive framework of professional identity formation. Reflections were then analysed longitudinally within and across individual portfolios to examine the professional identity formation over time with respect to these themes.	Five major themes were associated with professional identity formation in medical students: prior experiences, role models, patient encounters, curriculum (formal and hidden) and societal expectations. Our longitudinal analysis shows how these themes interact and shape pivotal moments, as well as the iterative nature of professional identity from the multiple ways in which individuals construct meaning from interactions with their environments.	Our study provides a window on the dynamic, discursive and constructed nature of professional identity formation. The five key themes associated with professional identity formation provide strategic opportunities to enable positive development. This study also illustrates the power of reflective writing for students and tutors in the professional identity formation process.
17	Zundel, S. Blumenstock, G.	2015	Portfolios Enhance Clinical	9.5	-	A change in German licensing legislation	The study was conducted with a modified pre-post design at the	Overall, 230 questionnaires were returned and analyzed;	The implementation of

	Zipfel, S. Herrmann-Werner, A. Holderried, F.		Activity in Surgical Clerks			imposed a portfolio for surgical clerks. We aimed to analyze whether the implementation of the portfolio changed the amount of clinical exposure and activities during surgical clerkships.	University Hospital of Tuebingen, Germany. Before and after the implementation of the portfolio on April 1, 2013, final-year students (n 1⁄4 557) who had just finished their surgical clerkship were interviewed with an online questionnaire. A total of 21 basic surgical skills were evaluated.	51% were preintervention. Overall clinical activity for the whole study cohort varied for different activities between 98% and 32%. For 16 of 21 parameters, there was more clinical activity in the postintervention (portfolio) group. This difference was statistically significant for the following 7 activities: discharge, analgesia, local infiltration, patient positioning, drain in, blood transfusion, and emergency diagnostics.	the portfolio did enhance clinical activity for surgical clerks in the study cohort. Nevertheless, overall exposure is still unsatisfactory low for some activities. Additional changes and studies are necessary to further improve surgical education.
18	Glenn Mason, Vicki Langendyk, Shaoyu Wang	2014	“The game is in the tutorial”: an evaluation of the use of an e-portfolio for personal and professional development in a medical school	7.5	10	An e-portfolio system was introduced into the personal and professional development curriculum at the University of Western Sydney School of Medicine (UWSSoM) during 2011 and 2013.	Adopting the methodology of Design-Based Research and an interpretive framework informed by Activity Theory we developed a set of educational design principles based on the analysis of the use of the e-portfolio system by students and tutors.	-	These principles contain a range of insights applicable to the local context and may also be of interest to curriculum designers working in other domains.
19	Nele R.M. Michels, Marijke Avonts, Griet Peeraer, Kris Ulenaers, Luc F. Van Gaal, Leo L. Bossaert, Erik W. Driessen, Arno M.M. Muijtjens & Benedicte Y. De Winter	2016	Content validity of workplace-based portfolios: A multi-centre study	9.5		Portfolios are used as tools to coach and assess students in the workplace. This study sought to evaluate the content validity of portfolios as reflected in their capacity to adequately assess achieved competences of medical students during clerkships.	We reviewed 120 workplace portfolios at three medical universities (Belgium and the Netherlands). To validate their content, we developed a Validity Inventory for Portfolio Assessment (VIPA) based on the CanMEDS roles. Two raters evaluated each portfolio and indicated for each VIPA item whether the portfolio provided sufficient information to enable satisfactory assessment of the item. We ran a descriptive analysis on the validation data	The portfolios adequately covered the items pertaining to the communicator (90%) and professional (87%) roles. Coverage of the medical expert, collaborator, scholar and manager roles ranged between 75% and 85%. The health advocate role, covering 59%, was clearly less well represented. This role also exhibited little interrater agreement (Kappa50.4).	This study lends further credence to the evidence that portfolios can indeed adequately assess the different CanMEDS roles during clerkships, the health advocate role, which was less well represented in the portfolio content, excepted.

							and computed Cohen's Kappa to investigate interrater agreement.		
20	Anthony J O'sullivan, Amanda C Howe, Susan Miles, Peter Harris, Chris S Hughes, Philip Jones, Helen Scicluna & Sam J Leinster	2012	Does a summative portfolio foster the development of capabilities such as reflective practice and understanding ethics? An evaluation from two medical schools	7.5	-	Portfolios need to be evaluated to determine whether they encourage students to develop in capabilities such as reflective practice and ethical judgment. The aims of this study were (i) to determine whether preparing a portfolio helps promote students' development in a range of capabilities including understanding ethical and legal principles, reflective practice and effective communication, and (ii) to determine to what extent the format of the portfolio affected the outcome by comparing the experiences of students at two different medical schools.	A questionnaire was designed to evaluate undergraduate medical students' experiences of completing a portfolio at two medical schools.	A total of 526 (45% response rate) students answered the on-line questionnaire. Students from both medical schools gave the highest ranking for the portfolio as a trigger for reflective practice. 63% of students agreed their portfolio helped them develop reflective practice skills ($p < 0.001$), whereas only 22% disagreed. 48% of students agreed portfolios helped them understand ethical and legal principles whereas 29% disagreed ($p < 0.001$). In contrast, only 34% of students thought the portfolio helped them to develop effective communication.	Students perceive portfolio preparation as an effective learning tool for the development of capabilities such as understanding ethical and legal principles and reflective practice, whereas other capabilities such as effective communication require complementary techniques and other modes of assessment.
21	Celia Laird O'Brien, PhD, Sandra M. Sanguino, MD, MPH, John X. Thomas, PhD, and Marianne M. Green, MD	2016	Feasibility and Outcomes of Implementing a Portfolio Assessment System Alongside a Traditional Grading System	7	-	Portfolios are a powerful tool to collect and evaluate evidence of medical students' competence across time. However, comprehensive portfolio assessment systems that are implemented	In 2009, the authors developed a portfolio system that served as a repository for all student assessments organized by competency domain. Five competencies were selected for a preclerkship summative portfolio review. Students submitted reflections on their performance. In 2014, four	Faculty evaluated 156 portfolios and showed high levels of agreement in their ratings. The majority of students achieved the "progressing toward competence" benchmark in all competency areas. However, 31 students received at least one	The portfolio review process allowed faculty to identify students with a concerning rating in a behavioral competency who would not have been identified in

						alongside traditional graded curricula at medical schools in the United States have not been described in the literature. This study describes the development and implementation of a longitudinal competency-based electronic portfolio system alongside a graded curriculum at a relatively large U.S. medical school.	clinical faculty members participated in standard-setting activities and used expert judgment and holistic review to rate students' competency achievement as "progressing toward competence," "progressing toward competence with some concern," or "progressing toward competence pending remediation." Follow-up surveys measured students' and faculty members' perceptions of the process.	concerning rating, which was not reflected in their course grades. Students' perceptions of the system's ability to foster self-assessment were mixed.	a traditional grading system. Identification of these students allows for intervention and early remediation.
22	Pitkälä, K. H. Mäntyranta, T.	2003	Feelings related to first patient experiences in medical school A qualitative study on students' personal portfolios	-	14	Feelings and thoughts of medical students related to first patient experiences during the first clinical year were examined. Twenty-two volunteer third and fourth year medical students (15 women and 7 men) of the University of Helsinki participated in a portfolio course for 1 year.	Their reflective learning diaries and writings on specific themes were analyzed by qualitative content analysis.	First patient encounters were strong emotional experiences for medical students. The first patient examination was often described as an anxiety-provoking and confusing incident. Other emotionally significant encounters included helplessness when faced with serious illness and death, and role confusion when examining patients of one's own age but opposite sex. Students felt guilty for using patients for their own learning purposes.	Portfolios as learning tools may help in recognizing key experiences and support professional development of medical students.
23	Anthony J. O'Sullivan , Peter Harris , Chris S. Hughes , Susan M. Toohey , Chinthaka	2012	Linking assessment to undergraduate student capabilities through portfolio examination	8.5	-	Portfolios are an established method of assessment, although concerns do exist around their validity for capabilities such as reflection and self-	-	-	Further research is required to evaluate acceptability to students, as well as the efficacy of portfolios in

	Balasooriya , Gary Velan , Rakesh K. Kumar & H. Patrick McNeil					direction. This article describes an e-portfolio which closely aligns learning and reflection to graduate capabilities, incorporating features that address concerns about portfolios. Students are required to complete assessments linked to graduate capabilities. In Year 3, a portfolio review occurs (205–248 students per year), focusing on students' grades and feedback from assessments and a reflective essay is submitted. In the essay, students reflect on their progress, identify areas of weakness and detail plans for improvement. Progress in each capability is summatively graded against specific criteria and feedback is provided. Students progressively accumulate evidence of learning linked to the graduate capabilities. The provision of sufficient structure prevents evasion of areas of weakness.			developing reflective practice and self-directed learning.

						Importantly, the equal weighting given to all graduate capabilities emphasises that competence in all areas is required. The requirement for a degree of self-direction and reflection in all assessments promotes regular review of progress. This e-portfolio explicitly links graduate outcomes with assessment in order to drive learning.			
24	Richard A. Prayson · S. Beth Bierer · Elaine F. Dannefer	2016	Medical student resilience strategies: A content analysis of medical students' portfolios	-	12	Stress and burnout among medical students is a well-recognized concern. A student's ability to employ resilience strategies to self-regulate behaviour is critical to the student's future career as a physician.	We retrospectively reviewed a sampling of year 1, 2 and 5 portfolio essays focused on the Personal Development competency and performance milestones, written by 49 students from three different classes in a 5-year programme devoted to training physician investigators. Two medical educators used a framework established by Jensen and colleagues (2008) to identify the nature and prevalence of various resilience strategies (valuing the physician role, self-awareness, personal arena, professional arena, professional support and personal support) medical students reported in portfolio essays.	All students documented at least one strategy in their essays each year. In all years, the most commonly documented strategies were in the personal arena (95.7% of year 1, 98% of year 2 and 87.8% of year 5 portfolios). The least frequently documented strategy in all years was professional support (42.8% of year 1, 38.8% of year 2, and 28.6% of year 5 portfolios). Year 5 portfolios discussed personal support strategies (79.6%) more frequently than year 1 (53.1%) and year 2 (59.2%) portfolios.	The results suggest that medical students can identify stressors and articulate resilience strategies that can be employed to potentially address them.
25	NRM Michels, EW Driessen, AMM Muijtjens, LF Van Gaal,	2009	Portfolio Assessment during Medical Internships: How	9.5	-	A portfolio is used to mentor and assess students' clinical performance at the	The domain-oriented reliability of 61 double-rated portfolios was measured, using a generalisability analysis with	We obtained reliability (Φ coefficient) of 0.87 with this internship portfolio containing 15 double-rated	Our study shows that a portfolio can be a reliable method for the

	LL Bossaert, BY De Winter		to Obtain a Reliable and Feasible Assessment Procedure?			workplace. However, students and raters often perceive the portfolio as a time-consuming instrument. In this study, we investigated whether assessment during medical internship by a portfolio can combine reliability and feasibility.	portfolio tasks and raters as sources of variation in measuring the performance of a student.	tasks. The generalisability analysis showed that an acceptable level of reliability (Φ = 0.80) was maintained when the amount of portfolio tasks was decreased to 13 or 9 using one and two raters, respectively.	assessment of workplace learning. The possibility of reducing the amount of tasks or raters while maintaining a sufficient level of reliability suggests an increase in feasibility of portfolio use for both students and raters.
26	Nilgün ÖZÇAKAR, Vildan MEVSİM, Dilek GÜLDAL	2009	Use of Portfolios in Undergraduate Medical Training: First Meeting With a Patient	-	10	Portfolios, as learning tools, are becoming increasingly important in medical education. Our aim was to evaluate the contents of portfolios used in medical education.	We designed a portfolio that was aimed at making reflection in early undergraduate medical education. Conditions for portfolio use were identified through interviews with six trainers who were experienced in mentoring students in the process of developing their portfolios. Analysis of writings was performed using qualitative content analysis.	The conditions for successful reflective use of portfolios that emerged from the interviews fell into categories: practice evaluation, patient perspective and primary prevention/health promotion. According to the students, working with a portfolio designed to meet these conditions is very useful and will stimulate students' abilities. The students shared the same opinion that analyzing one's competences in a portfolio was instructive and meaningful.	According to our students, portfolios are a potentially valuable method of developing students' skills in undergraduate medical education, provided that certain conditions for effective portfolios are recognized and met. The portfolio is a worthwhile addition to existing assessment and learning tools.
27	Celia Laird O'Brien, PhD, John X. Thomas Jr, PhD, and Marianne M. Green, MD	2018	What Is the Relationship Between a Preclerkship Portfolio Review and Later	9.5	-	Medical educators struggle to nd effective ways to assess essential competencies such as communication, professionalism, and	The authors divided students into two groups based on a summative preclerkship portfolio review in 2014: students who had concerning behavior in one or more competencies and students progressing satisfactorily. They	Students with concerning behavior preclerkship received signi cantly lower clerkship grades than students progressing satisfactorily (P = .002). They also scored signicantly	The results show a preclerkship portfolio review can identify behaviors that impact clerkship performance. A

			Performance in Clerkships?			teamwork. Portfolio-based assessment provides one method of addressing this problem by allowing faculty reviewers to judge performance, as based on a longitudinal record of student behavior. At the Feinberg School of Medicine, the portfolio system measures behavioral competence using multiple assessments collected over time. This study examines whether a preclerkship portfolio review is a valid method of identifying problematic student behavior affecting later performance in clerkships.	compared how students in these groups later performed on two clerkship outcomes as of October 2015: nal grades in required clerkships, and performance on a clerkship clinical composite score. They used Mann–Whitney tests and multiple linear regression to examine the relationship between portfolio review results and clerkship outcomes. They used USMLE Step 1 to control for knowledge acquisition.	lower on the clinical composite score (P < .001). Regression analysis indicated concerning behavior was associated with lower clinical composite scores, even after controlling for knowledge acquisition.	comprehensive portfolio system is a valid way to measure behavioral competencies.
28	Amin, T. T., Kaliyadan, F. and Al-Muhaidib, N. S.	2011	Medical students' assessment preferences at King Faisal University, Saudi Arabia	8.5	-	To assess the preferred methods for assessment among medical students at both preclinical and clinical stages of medical education and the possible correlates that promote these preferences.	All medical students from the third year onwards were surveyed. A self-administered anonymous questionnaire was designed to gather information on the preferred assessment method for course achievement. The preferred methods were also evaluated in relation to cognitive functions. Preference for specific exam format, in the form of multiple choices, short essay questions, or both, and the stated reasons for that preference, was also included in the questionnaire.	Out of 310 questionnaires distributed, 238 were returned. Written tests, projects, portfolios, and take home exams were the preferred modes for assessing students' achievements in a course; oral tests including a viva voce were the least preferred type of assessment. Questions that tested the domains of 'understanding' and 'application' were the most preferred type while those	Students' assessments at the College of Medicine, King Faisal University, Saudi Arabia, do not use the full range of cognitive domains. The emphasis on higher domains for medical students' assessment incorporating critical thinking should increase

								entailing ‘analysis’ were the least preferred. Multiple choice question format was the most preferred type of question (68.7%) at both pre and clinical stages.	as the students’ progress through their medical courses.
29	Arntfield, S., Parlett, B., Meston, C. N., Apramian, T. and Lingard, L.	2016	A model of engagement in reflective writing-based portfolios: Interactions between points of vulnerability and acts of adaptability	7	21	Portfolios are widely used for meeting new accreditation standards in the age of competency-based medicine. However, the method of learning through portfolio has been suggested to be vulnerable. The aim of this study was to explore conditions affecting the experience of teaching and learning from the perspective of both students and mentors in a reflective writing-based portfolio initiative.	Using mixed-methods rooted in grounded theory, 139 students and 13 mentors completed questionnaires, 23 students participated in four focus groups and 9 mentors in individual interviews.	The overarching theme in our data was student–mentor engagement. Our results confirm previous literature describing portfolio as a vulnerable method of learning, extend this concept by identifying and categorizing specific points of vulnerability, and contribute new knowledge regarding acts of adaptability, which serve to strengthen the student–mentor relationship.	Engagement is central to the success of portfolio and is shaped by a dynamic interaction between points of vulnerability and acts of adaptability. We propose a model of engagement in portfolio that can be used for faculty development to optimize student–mentor engagement.
30	Austin, C. and Braidman, I.	2008	Support for portfolio in the initial years of the undergraduate medical school curriculum: what do the tutors think?	7	18	Personal and Professional Development portfolios have recently been introduced into phase 1 (years 1 and 2) of the undergraduate medical curriculum in the School of Medicine in Manchester. As the large student numbers at Manchester precluded the use of an individual	A mixed method approach was used with data collected from both questionnaires and a focus group meeting.	Tutor facilitators were generally positive about their roles in the support of portfolio development in group sessions and identified several advantages to this type of tutoring – namely the value of group discussion and discussion between peers to encourage reflection, and the practical ability to integrate portfolio sessions more closely with clinical experiences – which would not be possible in one to one	Thus, tutors perceived that the support of portfolio in groups is an effective way of developing portfolios for large numbers of students.

						mentoring system, students are supported in their portfolio development by tutor facilitators, in groups of 10–12. The aims of this study were to investigate the views of these tutor facilitators on the delivery and support of portfolios in this way.		tutor/student meetings. With the training and guidance notes provided tutors were confident that they understood the support they should give students and felt that they could encourage reflection.	
31	Avila, J., Sostmann, K., Breckwoldt, J. and Peters, H.	2016	Evaluation of the free, open source software WordPress as electronic portfolio system in undergraduate medical education	-	10	Electronic portfolios (ePortfolios) are used to document and support learning activities. E-portfolios with mobile capabilities allow even more flexibility. However, the development or acquisition of ePortfolio software is often costly, and at the same time, commercially available systems may not sufficiently fit the institution's needs. The aim of this study was to design and evaluate an ePortfolio system with mobile capabilities using a commercially free and open source software solution.	We created an online ePortfolio environment using the blogging software WordPress based on reported capability features of such software by a qualitative weight and sum method. Technical implementation and usability were evaluated by 25 medical students during their clinical training by quantitative and qualitative means using online questionnaires and focus groups.	The WordPress ePortfolio environment allowed students a broad spectrum of activities – often documented via mobile devices – like collection of multimedia evidences, posting reflections, messaging, web publishing, ePortfolio searches, collaborative learning, knowledge management in a content management system including a wiki and RSS feeds, and the use of aid tools for studying. The students' experience with WordPress revealed a few technical problems, and this report provides workarounds. The WordPress ePortfolio was rated positively by the students as a content management system (67 % of the students), for exchange with other students (74 %), as a note pad for reflections (53 %)	It is possible to build an advanced ePortfolio system with mobile capabilities with the free and open source software WordPress. This allows institutions without proprietary software to build a sophisticated ePortfolio system adapted to their needs with relatively few resources. The implementation of WordPress should be accompanied by introductory courses in the use of the software and its apps in order to facilitate its usability.

								and for its potential as an information source for assessment (48 %) and exchange with a mentor (68 %). On the negative side, 74 % of the students in this pilot study did not find it easy to get started with the system, and 63 % rated the ePortfolio as not being user-friendly. Qualitative analysis indicated a need for more introductory information and training.	
32	Bashook, Philip, Gelula, Mark, Joshi, Medha and Sandlow, Leslie	2008	Impact of student reflective e-portfolio on medical student advisors	-	13	Little is published about the role of faculty advisors and use of students' e-portfolios. Purpose: This article reports advisors' observations and understanding about 1st-year students based on information from students' journaling as part of an e-portfolio.	Data were collected on BlackboardTMsurvey module for 8 volunteer advisors at two medical school campuses. Responses were hand coded, verified by two authors, tallied, with example comments recorded.	The four male and four female advisors are all mid-career, mixed between clinical and nonclinical faculty. The students' responses in the portfolio gave advisors greater insight into students' thinking, maturity, and reflective ability, and they helped advisors identify early warnings about problems. The e-portfolio enhanced meaningful interactions and more focused discussions with students. Advisors reported no improvements in efficiency of communications and had technical difficulties with BlackboardTM(version 6.0).	Advisors reported students' reflective responses to focused questions in an e-portfolio contribute valuable understanding about students' thinking and attitudes. Advisors are enthusiastic about the value of the e-portfolio for this purpose. We anticipate benefits will generalize when fully implemented.
33	Belcher, R., Jones, A., Smith, L. J., Vincent, T., Naidu, S. B., Montgomery, J.,	2014	Qualitative study of the impact of an authentic electronic portfolio in undergraduate	-	16	Portfolios are increasingly used in undergraduate and postgraduate medical education. Four medical schools have collaborated with an	To gather data, a questionnaire survey with extensive free text comments was used at School 1, and three focus groups were held at School 2. This paper reports thematic analysis of students'	Five main themes, common across both schools were identified. These concerned the purpose, use and acceptability of the portfolio, advantages of and barriers to the use of the portfolio,	An authentic portfolio mitigated some of the negative aspects of using a portfolio, and had a positive effect

	Haq, I. and Gill, D.		medical education			established NHS electronic portfolio provider to develop and implement an authentic professional electronic portfolio for undergraduate students. We hypothesized that using an authentic portfolio would have significant advantages for students, particularly in familiarizing them with the tool many will continue to use for years after graduation. This paper describes the early evaluation of this undergraduate portfolio at two participating medical schools.	opinions expressed in the free text comments and focus groups.	and the impacts on both learning and professional identity.	on students' perception of themselves as becoming past of the profession. However, significant barriers to portfolio use remained, including a lack of understanding of the purpose of a portfolio and a perceived damaging effect on feedback.
34	Beth Bierer, S and Dannefer, Elaine F	2011	Portfolio-Based Assessment of Students-Does Students' Gender, Citizenship, or Verbal Ability Affect Fairness of Portfolio-Based Promotion Decisions? Results From One Medical School	10	-	Measurement experts use four criteria to examine the fairness of tests: (1) equitable treatment for examinees,<br>(2) equal outcomes for subgroups,<br>(3) absence of bias, and (4) equal opportunity to learn. These criteria apply to portfolios just as they do to other assessments. This report examines the fairness of portfolio-based promotion	Participants were 182 first-year medical students (97 men, 85 women) from six class cohorts (2004 –2009). Chi-square statistics with Yates continuity correction were used to compare overall promotion decisions to students' gender, self-reports of language fluency, and MCAT Writing Sample score. The Cramér's V statistic served as an effect size index. Post hoc power analyses identified the minimum sample size to obtain acceptable power.	Approximately 85% of students were promoted to Year 2 of the program. Gender, U.S. citizenship, language fluency, and MCAT Writing Sample score were not significantly related to overall promotion decisions. Effect sizes were small (0.15) for all contingency tables, suggesting weak associations between overall promotion decisions and students' group characteristics.	Examining fairness, although challenging, is essential to maintain professional standards and avoid potential liability. Preliminary evidence in this study suggests that students' background characteristics and verbal abilities were not strongly related to

						decisions for medical students at the Cleveland Clinic Lerner College of Medicine.			portfolio-based promotion decisions. Schools should monitor processes that may affect fairness. This study reports on just one aspect of fairness. More research is needed to evaluate other dimensions of fairness.
35	Bierer, S. B., Dannefer, E. F. and Tetzlaff, J. E.	2015	Time to Loosen the Apron Strings: Cohort-based Evaluation of a Learner-driven Remediation Model at One Medical School	10	-	Remediation in the era of competency-based assessment demands a model that empowers students to improve performance. This study aims to examine a remediation model where students, rather than faculty, develop remedial plans to improve performance. It takes place in a private medical school with 177 medical students. A promotion committee uses student-generated portfolios and faculty referrals to identify struggling students, and has them develop formal remediation plans with personal reflections, improvement	-	Participants included 177 students from six classes (2009–2014). Twenty-six were placed in remediation, with more referrals occurring during Years 1 or 2 (n=20, 76 %). Unprofessional behavior represented the most common reason for referral in Years 3–5. Remedial students did not differ from classmates (n=151) on baseline characteristics (Age, Gender, US citizenship, MCAT) or willingness to recommend their medical school to future students (p<0.05). Two remedial students did not graduate and three did not pass USLME licensure exams on first attempt. Most remedial students (92 %) generated appropriate plans to address performance deficits.	Students can successfully design remedial interventions. This learner-driven remediation model promotes greater autonomy and reinforces self-regulated learning.

						strategies, and performance evidence. Students submit reports to document progress until formally released from remediation by the promotion committee.			
36	Borgstrom, E., Cohn, S. and Barclay, S.	2010	Medical professionalism: conflicting values for tomorrow's doctors	-	14	New values and practices associated with medical professionalism have created an increased interest in the concept. In the United Kingdom, it is a current concern in medical education and in the development of doctor appraisal and revalidation. This study aims to investigate how final year medical students experience and interpret new values of professionalism as they emerge in relation to confronting dying patients and as they potentially conflict with older values that emerge through hidden dimensions of the curriculum.	Qualitative study using interpretative discourse analysis of anonymized student reflective portfolios. One hundred twenty-three final year undergraduate medical students (64 male and 59 female) from the University of Cambridge School of Clinical Medicine supplied 116 portfolios from general practice and 118 from hospital settings about patients receiving palliative or end of life care.	Professional values were prevalent in all the portfolios. Students emphasised patient-centered, holistic care, synonymous with a more contemporary idea of professionalism, in conjunction with values associated with the 'old' model of professionalism that had not be directly taught to them. Integrating 'new' professional values was at times problematic. Three main areas of potential conflict were identified: ethical considerations, doctor-patient interaction and subjective boundaries. Students explicitly and implicitly discussed several tensions and described strategies to resolve them.	The conflicts outlined arise from the mix of values associated with different models of professionalism. Analysis indicates that 'new' models are not simply replacing existing elements. Whilst this analysis is of accounts from students within one UK medical school, the experience of conflict between different notions of professionalism and the three broad domains in which this conflict arises are relevant in other areas of medicine and in different national contexts.
37	Burch, V. C. and Seggie, J.	2005	Portfolio assessment using a structured interview	8.5	-	The use of portfolios, as a learning and assessment tool, has gained considerable popularity in the past	During the fourth-year medicine clerkship, students wrote up 25 patient encounters during a 14-week clerkship and reflected on these by: (a) editing (in another	The results of the fourth-year class of 2004 (n ¼ 181) were evaluated. The mean (± SD, 95% CI) portfolio interview score	-

						decade. We adopted portfolios as part of our assessment package in the clinical clerkships (years 4–6) of our extensively revised MBChB programme in 2002. The study was necessary because a major limitation of portfolios is the resource-intensive nature of the assessment process. Published data report examination times exceeding 90 minutes per candidate. In resource-constrained environments, typical of developing countries, this time requirement is prohibitive.	colour ink) their clerking notes after discussion with senior clinicians and review of standard reference texts, and (b) formulating written question and answer tasks regarding some aspect of individual patient encounters. The latter were dictated largely by individual student learning needs with guidance by clinician tutors where necessary. At the end of the year portfolios were examined by interview. Four patient encounters were discussed during a 30-minute single-examiner interview; candidates selected 1 case and examiners the remaining 3 cases. Using 5 structured questions, candidates' ability to synthesise clinical assessments, using information gathered during patient encounters, was explored. The questions determined whether candidates had (a) defined clearly the patient's presenting problem; (b) offered a pathophysiologically plausible diagnosis; (c) substantiated the diagnosis using available clinical and investigatory findings; (d) considered a reasonable differential diagnosis; and (e) formulated a reasonable management plan. Responses to questions were rated using a 9-point global rating scale: poor (1–3), adequate (4– 6) and good (7–9). Clinician examiners assigned a final percentage score to each case using a criterion-referenced	achieved was 67.5% (± 10.5, 66–69.1). The correlation coefficients for the portfolio interview, when compared to the multiple choice written examination and clinical case-based examination, were, respectively, r 1⁄4 0.42, and r 1⁄4 0.37. Cronbach's alpha coefficient for the portfolio interview was 0.88 with an inter-item correlation of 0.66. The SEM was 3.6. The single-examiner portfolio interview, using standardised questions and a global rating scale, demonstrates high internal consistency and broadens the spectrum of assessment currently achieved. This method, requiring less examination time per candidate than published data, has been added to the array of assessment tools used in the clinical clerkship rotations in our new programme.	

							scale: 3 or more questions rated ‘poor’ scored 45% or less, 2 or fewer questions rated ‘poor’ scored 52–58%, all questions rated ‘adequate’ scored 60–62%, 2 or fewer questions rated ‘good’ scored 65– 68%, at least 3 questions rated ‘good’ scored 70–74% and all questions rated ‘good’ score 75% or more. Clinician examiners were trained before implementing this assessment tool. Pearson’s correlation coefficient, Cronbach’s alpha coefficient and the standard error of measurement (SEM) of the assessment tool were determined using STATISTICA (Statsoft).		
38	Burch, V. C. and Seggie, J. L.	2008	Use of a structured interview to assess portfolio-based learning	10.5	-	Portfolio-based learning is a popular educational tool usually examined by document review which is sometimes accompanied by an oral examination. This labour-intensive assessment method prohibits its use in the resource-constrained settings typical of developing countries. The study aims to determine the feasibility and internal consistency of a portfolio-based structured interview and its impact on	Year 4 medical students (n = 181) recorded 25 patient encounters during a 14-week medical clerkship. Portfolios were examined in a 30-minute, single-examiner interview in which four randomly selected cases were discussed. Six standard questions were used to guide examiners in determining the ability of candidates to interpret and synthesise clinical data gathered during patient encounters. Examiners were trained to score responses using a global rating scale. Pearson’s correlation co-efficient, Cronbach’s a coefficient and the standard error of measurement (SEM) of the assessment tool were determined. The number of students completing more than	The mean (± standard deviation [SD], 95% confidence interval [CI]) interview score was 67.5% (SD ± 10.5, 95% CI 66.0–69.1). The correlation coefficients for the interview compared with other component examinations of the assessment process were: multiple-choice question (MCQ) examination 0.42; clinical case-based examination 0.37; in-course global rating 0.08, and overall final score 0.54. Cronbach’s a coefficient was 0.88 and the SEM was 3.6. Of 181 students, 45.3% completed more than 25 portfolio entries.	Portfolio assessment using a 30-minute structured interview is a feasible, internally consistent assessment method that requires less examination time per candidate relative to methods described in published work and which may encourage desirable student learning behaviour.

						student learning behaviour.	the required number of portfolio entries was also recorded.		
39	Byszewski, A., Fraser, A. and Lochnan, H.	2018	East meets West: Shadow coaching to support online reflective practice	-	4	A structured, reflection-based electronic portfolio program (ePortfolio), with novel faculty development initiative, involving ‘shadow coaches’, was shared with the newly formed Ottawa-Shanghai Joint School of Medicine (OSJSM). OSJSM is a partnership between Shanghai Jiao Tong University and the University of Ottawa. As the world’s first Sino-Canadian Joint Medical School, OSJSM introduced North American undergraduate medical curriculum to China. ‘Shadow coaching’ involved trans-Pacific pairing of coaches, supplemented by local faculty development.	(a) Pre-implementation: The well-established online ePortfolio platform at the University of Ottawa was mirrored at OSJSM. University of Ottawa ePortfolio coaches were recruited to serve as shadow coaches to their OSJSM counterparts. Shadow coaches provided mentoring and resources while maintaining awareness of cross-cultural issues. Faculty development consisted of face-to-face faculty development in Shanghai, several online synchronous sessions, and familiarization of University of Ottawa coaches with the Chinese medical education system. (b) Description/Components: This intervention, introduced in 2016–2017, involved five University of Ottawa shadow coaches paired with five OSJSM ePortfolio coaches. Student reflection encourages open frank discussion which is a new paradigm for Chinese students and faculty. Shadow coaches were encouraged to challenge new OSJSM coaches to widely explore physician roles and competencies.	Initial results indicate that the experience served to effectively develop OSJSM coaches’ skills as evidenced by shadow coaches’ review of anonymized OSJSM student reflective writing.	Our project describes a novel tool using shadow coaching for faculty development for a cross-cultural partnership. Similar approaches can be utilized for culturally-sensitive long-distance faculty development.
40	Chaffey, L. J., de Leeuw, E. J. and Finnigan, G. A.	2012	Facilitating students' reflective practice in a medical course: literature review	-	12	Reflection and reflective practice is of increasing importance in medical education curricula. The aim of this review is to summarise the	A review of the literature was undertaken using de ned databases and the search terms ‘medical students’, ‘medical education’, ‘reflection’, ‘reflect*’ and ‘medicine’. e search was limited to peer-reviewed	Thirty-six relevant articles were found, identifying enhancing factors and barriers to effectively teaching reflective practice within medical curricula, relating to: The breadth of	A variety of reflective purposes was found in this literature review. Evidence indicates that, if

						literature published around facilitating reflection in a medical course, and to answer the question: What is the current evidence regarding learning and development moments across the medical curriculum in developing students' reflective practice?	published material in English and between the years 2001 and 2011, and included research, reviews and opinion pieces.	the meaning of reflection; facilitating reflection by medical educators; using written or web-based portfolios to facilitate reflection; and assessing the reflective work of students.	students are unclear as to the purpose of reflection and do not see educators modelling reflective behaviours, they are likely to undervalue this important skill regardless of the associated learning and development opportunities embedded in the curriculum.
41	Chertoff, Jason, Wright, Ashleigh, Novak, Maureen, Fantone, Joseph, Ahmed, Toufeeq, Green, Marianne M, Kalet, Adina, Jacobs, Joshua, Dokter, Christina and Zaidi, Zareen	2015	Status of portfolios in undergraduate medical education in the LCME accredited US medical school	7.5	16	Aim: We sought to investigate the number of US medical schools utilizing portfolios, the format of portfolios, information technology (IT) innovations, purpose of portfolios and their ability to engage faculty and students.	A 21-question survey regarding portfolios was sent to the 141 LCME-accredited, US medical schools. The response rate was 50% (71/141); 47% of respondents (33/71) reported that their medical school used portfolios in some form. Of those, 7% reported the use of paper-based portfolios and 76% use electronic portfolios. Forty-five percent reported portfolio use for formative evaluation only; 48% for both formative and summative evaluation, and 3% for summative evaluation alone.	Seventy-two percent developed a longitudinal, competency-based portfolio. The most common feature of portfolios was reflective writing (79%). Seventy-three percent allow access to the portfolio off-campus, 58% allow usage of tablets and mobile devices, and 9% involve social media within the portfolio. Eighty percent and 69% agreed that the portfolio engaged students and faculty, respectively. Ninety-seven percent reported that the portfolios used at their institution have room for improvement.	While there is significant variation in the purpose and structure of portfolios in the medical schools surveyed, most schools using portfolios reported a high level of engagement with students and faculty.
42	Chiu, Y. T., Lee, K. L. and Ho, M. J.	2014	Effects of feedback from near-peers and non-medical	7	-	Portfolios have grown increasingly popular in medical education as a result of the emphasis on	From September 2012 to January 2013, all Year 2 medical students (n = 131) taking a required course on medicine and society at National Taiwan	The results show that students found the feedback from near-peers and non-medical professionals beneficial. Students agreed	This study found that feedback from near-peers and non-medical professionals can

			professionals on portfolio use			competency and reflection in contemporary health professional education. The portfolio, a collection of evidence demonstrating learning and reflection, is a prevalent assessment tool in both undergraduate and post-graduate medical education. The medical education literature suggests mixed success in the use of portfolios, and previous research has reported that feedback from mentors is a crucial contributor to success. In addition to teachers and peers, who commonly act as mentors, might near-peers, who may be more experienced than peers and more approachable than teachers, be able to support medical students engaged in the portfolio process? Furthermore, can non-medical professionals provide alternative perspectives that stimulate medical students to develop and reflect on the competencies they	University submitted three e-portfolio entries reflecting on their development in key professional competencies, such as humanism and communication. In addition to feedback from teachers and peers, each student was matched to one senior medical student (i.e. near-peer) and one non-medical professional (a category including standardised patients and education researchers) trained to provide feedback. The e-portfolios were scored according to a rubric for reflection.1 At the end of the semester, 81 students completed a questionnaire consisting of 5-point Likert scale-based and openended questions. Research data were analysed using SPSS for Windows V16.0.1 (SPSS Inc., Chicago, IL, USA).	that feedback not only helped them in writing their e-portfolios (mean scores: 3.43–3.49; standard deviations [SDs]: 0.77–0.82), but also enhanced their self-reflection (mean scores: 3.43–3.53; SDs: 0.79–0.92), especially in the case of near-peer feedback. In addition, the perceived effectiveness of near-peer feedback was correlated to portfolio scores ($r = 0.23$ and $r = 0.28$; $p < 0.05$). Answers to the open-ended questions supported the effectiveness of feedback from multiple sources. For instance, students stated: ‘. . .feedback from senior schoolmates helped a tremendous amount’; ‘...feedback from near peers and non-medical professionals enhanced my understanding of their expectations of medical students’, and ‘. . .receiving feedback from people previously unknown [i.e. a non-medical professional] makes me feel fulfilled.’	have a positive impact on medical students’ use of and reflection on their e-portfolios. Near-peer feedback was perceived to be more effective than that given by non-medical professionals. This may reflect the fact that students and their near-peers are of similar ages and backgrounds. Given these impacts, multiple sources of feedback, such as near-peers and non-medical professionals, should be considered when implementing a portfolio system.

						require to meet societal expectations of medical professionalism? The aim of this study is to bridge the gap in research and investigate the effects of feedback from near-peers and non-medical professionals on medical students' reflective portfolios.			
43	Cotterill, Simon, McDonald, Tony and Horner, Paul	2008	Using the ePET portfolio to support teaching and learning in Medicine: Lessons from 3 Institutions	-	1	There is a high level of interest in ePortfolios because of drivers for personal development planning (PDP) within the HE sector as well subject-specific drivers in areas such as Medicine.	This case study documents the educational aspects of implementing and embedding the ePET portfolio (http://www.eportfolios.ac.uk) at 3 Medical Schools in the UK.	-	-
44	Cunningham, H., Taylor, D., Desai, U. A., Quiah, S. C., Kaplan, B., Fei, L., Catallozzi, M., Richards, B., Balmer, D. F. and Charon, R.	2018	Looking Back to Move Forward: First-Year Medical Students' Meta-Reflections on Their Narrative Portfolio Writings	-	12	The day-to-day rigors of medical education often preclude learners from gaining a longitudinal perspective on who they are becoming. Furthermore, the current focus on competencies, coupled with concerning rates of trainee burnout and a decline in empathy, have fueled the search for pedagogic tools to foster students' reflective capacity. In response, many scholars have	In January 2015, the authors investigated learning outcomes derived from students' "Signature Reflections," end-of-semester meta-reflections of their previous portfolio work. The authors analyzed the Signature Reflections of 97 (of 132) first-year medical students using a constant comparative process. This iterative approach allowed researchers to identify themes within students' writings and interpret the data.	The authors identified two overarching interpretive themes—recognition and grappling—and six subthemes. Recognition included comments about self-awareness and empathy. Grappling encompassed the subthemes of internal change, dichotomies, wonder and questioning, and anxiety.	Based on the authors' analyses, the Signature Reflection seemed to provide a structured framework that encouraged students' reflective capacity and the construction of holistic professional identity. Other medical educators may adopt meta-reflection, within the reflective space of a writing portfolio, to

						looked to the tradition of narrative medicine to foster “reflective spaces” wherein holistic professional identity construction can be supported. This article focuses on the rationale, content, and early analysis of the reflective space created by the narrative medicine-centered portfolio at the Columbia University College of Physicians and Surgeons.			encourage students’ acquisition of a longitudinal perspective on who they are becoming and how they are constructing their professional identity.
45	Dannefer, E. F., Bierer, S. B. and Gladding, S. P.	2012	Evidence within a portfolio-based assessment program: what do medical students select to document their performance?	7	-	Decisions about performance in programs of assessment that provide an array of assessment evidence require judgments about the quality of different pieces of assessment data to determine which combination of data points best represent a trainee’s overall performance. In this article, we examine the nature of evidence selected by first-year medical students to include in a portfolio used to make promotion decisions.	We reviewed portfolios to examine the number, type, and source of assessments selected by students (n¼432) to document their performance in seven competencies. The quality of assessment data selected for each competency was rated by promotion committee members (n ¼ 14).	Findings indicate that students cited multiple types and sources of available assessments. The promotion committee rated evidence quality highest for competencies where the program provided sufficient evidence for students to cite a broad range of assessments. When assessments were not provided by the program, students cited self-generated evidence.	We found that when student-constructed portfolios are part of an overall assessment system, students generally select evidence in proportion to the number and types of assessments available.

46	Dannefer, E. F. and Prayson, R. A.	2013	Supporting students in self-regulation: use of formative feedback and portfolios in a problem-based learning setting	-	11	The widely recognized need for students to self-regulate their behavior and learning extends to the multiple dimensions of professionalism. This study examines the extent to which students self-regulate professionalism behaviors related to work habits and interpersonal skills in a PBL setting.	Formative feedback on works habits and interpersonal skills provided by peers and tutors to a Year 1 cohort (n 1⁄4 32) over the course of a year-long PBL experience (5 blocks) was examined for comments on targeted areas for improvement (TAFIs) and observed improvements. We examined congruence between PBL feedback and students' self-reported TAFIs and behavioral improvements in their assessment portfolios.	Both PBL peer and faculty feedback and portfolio self-assessments targeted Interpersonal Skills TAFIs more frequently than Work Habit-related issues. TAFIs were more frequently identified midway in PBL blocks versus the end. Students reported TAFIs in their portfolio essays, citing feedback from both peers and tutors, and provided evidence of improved performance over time.	Students utilized external formative feedback to document their portfolio self-assessment in a system designed to support self-regulation of PBL professionalism-related behaviors. A decrease in TAFIs identified at the end of PBL blocks suggests students made use of mid-block feedback to self-regulate behaviors.
47	Davis, MH, Ben-David, M Friedman, Harden, RM, Howie, Peter, Ker, Jean, McGhee, C, Pippard, MJ and Snadden, David	2009	Portfolio assessment in medical students' final examinations	7	-	The introduction of an outcome-based approach to education at Dundee Medical School in Scotland instigated a search for assessment methods that would appropriately assess the students' achievements in terms of the learning outcomes. Portfolio assessment has been developed for this purpose and has been adopted for the summative assessment of students in their final examination in Dundee. The contents	-	The evaluation of the approach demonstrated strong staff support. Students were also positive although with some reservations.	It is concluded that portfolio assessment is a powerful approach to assessing a range of curriculum outcomes not easily assessed by other methods and is worthy of inclusion in the assessor's toolkit.

						of the portfolio and the assessment process have been defined and the first cohort of students to be assessed in this way has been studied.			
48	Davis, M. H. and Ponnamperuma, G. G.	2010	Examiner perceptions of a portfolio assessment process	8	-	The portfolio assessment process is important for assessing learner achievement. This study aims to study examiner perceptions of Dundee Medical School's portfolio assessment process, in years 4 and 5 of the 5-year curriculum, in relation to: outcomes as a framework for the portfolio assessment process; portfolio content; portfolio assessment process; end points of the portfolio assessment process; appropriateness of the two part final exam format and examiner training.	A questionnaire containing statements and open questions was used to obtain examiner feedback. Responses to each statement were compared over 3 years: 1999, 2000 and 2003.	Response rates were 100%, 88% and 61% in 1999, 2002 and 2003, respectively. Examiners were positive about the ability of institutionally set learning outcomes (Dundee 12 exit learning outcomes) to provide a framework for the portfolio assessment process. They found difficulties, however, with the volume of portfolio content and the time allocated to assess it. Agreeing a grade for each learning outcome for the candidate with their co-examiner did not present difficulties. The comprehensive, holistic picture of the candidate provided by the portfolio assessment process was perceived to be one of its strengths. Examiners were supportive of the final examination format, and were satisfied with their briefing about the process.	The 12 exit learning outcomes of Dundee curriculum provide an appropriate framework for the portfolio assessment process, but the content of the portfolio requires fine-tuning particularly with regard to quantity. Time allocated to examiners for the portfolio assessment process needs to be balanced against practicability. The holistic picture of the candidate provided by the process was one of its strengths.
49	Davis, M. H., Ponnamperuma, G. G. and Ker, J. S.	2009	Student perceptions of a portfolio assessment process	8	-	The objectives of this study were to identify and analyse students' attitudes to the portfolio assessment process over time.	A questionnaire containing statements and open questions was used to obtain feedback from students at the University of Dundee Medical School, Scotland. The responses to each	Response rates were 83% in 1999, 70% in 2000, 89% in 2002 and 88% in 2003. A major finding is that students perceived that portfolio building heightened their	Paperwork should be kept within manageable limits. A student induction process that highlights the

							statement were compared over 4 years (1999, 2000, 2002 and 2003).	understanding of the exit learning outcomes and enabled reflection on their work. Student reactions to the portfolio process were initially negative, although they appreciated that senior staff took time to become familiar with their work through reviewing their portfolios. Student attitudes became more positive over the 4 years as the process evolved. Although portfolio assessment was recognised as supporting student learning, portfolio building was perceived to interfere with clinical learning as a result of the excessive amounts of paper evidence required.	importance of providing evidence for achieving all learning outcomes, not just theoretical knowledge and skills, may be helpful in allaying student concern over portfolio building and assessment and support preparation for lifelong learning and reflective clinical practice.
50	Deketelaere, A., Kelchtermans, G., Druine, N., Vandermeersch, E., Struyf, E. and De Leyn, P.	2009	Making more of it! Medical students' motives for voluntarily keeping an extended portfolio	-	14	Although medical students' use of portfolios has been studied from many angles, little is known about their motivations. This article aims to explore medical students' motives for voluntarily compiling a learning portfolio that widely exceeded the assignments.	Content analysis was performed on 22 (8%; n 1⁄4 22/269) extensive portfolios, followed by a semi-structured interview with 11 medical students. Building on the theoretical work of Simons et al. (2004), interpretative analysis was used to reconstruct and understand the medical students' motives for the effort they put into the portfolios.	Compiling an elaborate portfolio is mainly instigated by a personal instrumentality (internally regulated instrumental motivation). These medical students reflected on what they considered important and useful. The portfolio was a tool to achieve self-set goals, yet the specific goals turned out to be very different among the students, reflecting their particular needs and experiences during clerkship.	Motivation theory shows that students who are internally regulated use more deep-level learning strategies and perform better. Internally regulated motivation mainly occurs when students use the portfolio to achieve their self-set goals. The formal portfolio assignments, enforced by the medical school,

									were more related with externally regulated motivation.
51	Dolan, B. M., O'Brien, C. L., Cameron, K. A. and Green, M. M.	2018	A Qualitative Analysis of Narrative Preclerkship Assessment Data to Evaluate Teamwork Skills	-	15	Students entering the health professions require competency in teamwork. Background: Although many teamwork curricula and assessments exist, studies have not demonstrated robust longitudinal assessment of preclerkship students' teamwork skills and attitudes. Assessment portfolios may serve to fill this gap, but it is unknown how narrative comments within portfolios describe student teamwork behaviors.	We performed a qualitative analysis of narrative data in 15 assessment portfolios. Student portfolios were randomly selected from 3 groups stratified by quantitative ratings of teamwork performance gathered from small-group and clinical preceptor assessment forms. Narrative data included peer and faculty feedback from these same forms. Data were coded for teamwork-related behaviors using a constant comparative approach combined with an identification of the valence of the coded statements as either "positive observation" or "suggestion for improvement."	Eight codes related to teamwork emerged: attitude and demeanor, information facilitation, leadership, preparation and dependability, professionalism, team orientation, values team member contributions, and nonspecific teamwork comments. The frequency of codes and valence varied across the 3 performance groups, with students in the low-performing group receiving more suggestions for improvement across all teamwork codes.	Narrative data from assessment portfolios included specific descriptions of teamwork behavior, with important contributions provided by both faculty and peers. A variety of teamwork domains were represented. Such feedback as collected in an assessment portfolio can be used for longitudinal assessment of preclerkship student teamwork skills and attitudes.
52	Elango, S., Jutti, R. C. and Lee, L. K.	2005	Portfolio as a learning tool: students' perspective	6.5	-	Portfolio writing is a method of encouraging reflective learning among professionals. Although portfolio-based learning is popular among educators, not many studies have been done to determine students' perceptions	A questionnaire survey was conducted among 143 medical students to find out their perceptions of the portfolio as a learning tool.	A majority of students felt that the portfolio is a good learning tool. However, they also perceived that it is stressful and time-consuming to develop a proper portfolio.	The study indicates that students need appropriate guidance form the academic staff for the system to succeed.

						of portfolio as a learning tool.			
53	Haffling, A. C., Beckman, A., Pahlmblad, A. and Edgren, G.	2010	Students' reflections in a portfolio pilot: highlighting professional issues	-	14	Portfolios are highlighted as potential assessment tools for professional competence. Although students' self-reflections are considered to be central in the portfolio, the content of reflections in practice-based portfolios is seldom analysed. The aim of this study is to investigate whether students' reflections include sufficient dimensions of professional competence, notwithstanding a standardized portfolio format, and to evaluate students' satisfaction with the portfolio.	Thirty-five voluntary final-year medical students piloted a standardized portfolio in a general practice (GP) attachment at Lund University, Sweden. Students' portfolio reflections were based upon documentary evidence from practice, and aimed to demonstrate students' learning. The reflections were qualitatively analysed, using a framework approach. Students' evaluations of the portfolio were subjected to quantitative and qualitative analysis.	Among professional issues, an integration of cognitive, affective and practical dimensions in clinical practice was provided by students' reflections. The findings suggested an emphasis on affective issues, particularly on self-awareness of feelings, attitudes and concerns. In addition, ethical problems, clinical reasoning strategies and future communication skills training were subjects of several reflective commentaries. Students' reflections on their consultation skills demonstrated their endeavour to achieve structure in the medical interview by negotiation of an agenda for the consultation, keeping the interview on track, and using internal summarizing. The importance of active listening and exploration of patient's perspective was also emphasized. In students' case summaries, illustrating characteristic attributes of GP, the dominating theme was 'patient-centred care', including the patient–doctor relationship, holistic modelling and longitudinal continuity. Students were	A standardized portfolio in a defined course with a limited timeframe provided ample opportunities for reflections on professional issues. Support by mentors and a final examiner interview contributed to the success of the portfolio with students. The interview also allowed students to deepen their reflections and to receive feedback.

								satisfied with the portfolio, but improved instructions were needed.	
54	Rees, C. and Sheard, C.	2004	Undergraduate medical students' views about a reflective portfolio assessment of their communication skills learning	10	-	To date, no studies have examined preclinical medical students' views about portfolios. Since portfolios are becoming increasingly valued in medical education, this study explores second-year medical students' views about a reflective portfolio assessment of their communication skills.	178 second-year medical students at the University of Nottingham completed the 18-item reflective portfolio questionnaire (RPQ) (a 1⁄4 0.716) and a personal details questionnaire three days before submitting their portfolio assessment for communication skills. Data were analysed using univariate and multivariate statistics on SPSS Version 10.0.	Total scores on the RPQ ranged from 40 to 75 (mean 58.28, SD 7.08). Significant relationships existed between RPQ total scores and students' ratings of their reflection skills (rs = 0.322, P < 0.001), RPQ total scores and students' confidence building another portfolio (T = 4.381, d.f. = 176, P < 0.001), and RPQ total scores and students' marks for their reflective portfolio assessment (rs = 0.167, P = 0.029). Students with more positive views about reflective portfolios were more likely to rate their reflection skills as good, receive better marks for their portfolio assessment, and be more confident building another portfolio.	This study begins to highlight preclinical medical students' views about reflective portfolios. However, further research is required using qualitative studies to explore students' views in depth. Medical educators should be encouraged to consider introducing portfolios as a method of formative and summative assessment earlier in the medical curriculum.
55	Rees, C. E. and Sheard, C. E.	2004	The reliability of assessment criteria for undergraduate medical students' communication skills portfolios: the Nottingham experience	8.5	-	Some educators have argued that portfolios should not be assessed summatively because there is little evidence supporting the reliability of their assessment. This study aims to determine the reliability of assessment criteria used for a portfolio at	Two independent analysts assessed a random sample of portfolios (n = 100, 49.5%) using criterion-referenced assessment. Students' performances were examined against subjective items in five areas: 1) portfolio structure, 2) level of critical reflection, 3) level of skills development, 4) use of documentary evidence, and 5) use of relevant literature. These subjective judgements were later converted into quantitative scales	The level of agreement between the two raters for the total percentage score was 0.771 (95% CI = 0.678, 0.840), as measured by an intraclass correlation coefficient. The levels of agreement between the two raters for the individual items of the assessment criteria ranged from j = 0.359 (item 3) to j = 0.693 (item 4).	This study provides some support for the summative assessment of portfolios. The findings suggest that discussion and negotiation between independent assessors can enhance the reliability of

						the University of Nottingham.	ranging from 0 to 3 so that interrater reliability could be established. The level of agreement between the two analysts for the total percentage score was established using an intraclass correlation coefficient and for the individual items using weighted kappa coefficients.		assessment criteria. Therefore, medical educators are encouraged to use such procedures in the summative assessment of portfolios.
56	Roberts, C., Shadbolt, N., Clark, T. and Simpson, P.	2014	The reliability and validity of a portfolio designed as a programmatic assessment of performance in an integrated clinical placement	11.5	-	Little is known about the technical adequacy of portfolios in reporting multiple complex academic and performance-based assessments. We explored, first, the influencing factors on the precision of scoring within a programmatic assessment of student learning outcomes within an integrated clinical placement. Second, the degree to which validity evidence supported interpretation of student scores.	Within generalisability theory, we estimated the contribution that each wanted factor (i.e. student capability) and unwanted factors (e.g. the impact of assessors) made to the variation in portfolio task scores. Relative and absolute standard errors of measurement provided a confidence interval around a pre-determined pass/fail standard for all six tasks. Validity evidence was sought through demonstrating the internal consistency of the portfolio and exploring the relationship of student scores with clinical experience.	The mean portfolio mark for 257 students, across 372 raters, based on six tasks, was 75.56 (SD, 6.68). For a single student on one assessment task, 11% of the variance in scores was due to true differences in student capability. The most significant interaction was context specificity (49%), the tendency for one student to engage with one task and not engage with another task. Rater subjectivity was 29%. An absolute standard error of measurement of 4.74%, gave a 95% CI of +/- 9.30%, and a 68% CI of +/- 4.74% around a pass/fail score of 57%. Construct validity was supported by demonstration of an assessment framework, the internal consistency of the portfolio tasks, and higher scores for students who did the clinical placement later in the academic year.	A portfolio designed as a programmatic assessment of an integrated clinical placement has sufficient evidence of validity to support a specific interpretation of student scores around passing a clinical placement. It has modest precision in assessing students' achievement of a competency standard. There were identifiable areas for reducing measurement error and providing more certainty around decision-making. Reducing the measurement error would require engaging with the student body on the value

									of the tasks, more focussed academic and clinical supervisor training, and revisiting the rubric of the assessment in the light of feedback.
57	Ross, S., Maclachlan, A. and Cleland, J.	2009	Students' attitudes towards the introduction of a Personal and Professional Development portfolio: potential barriers and facilitators	-	20	Portfolios, widely used in undergraduate and postgraduate medicine, have variable purposes, formats and success. A recent systematic review summarised factors necessary for successful portfolio introduction but there are no studies investigating the views of students inexperienced in portfolio use towards portfolio learning. This study's aim was to survey student views about a prospective Professional and Personal Development (PPD) portfolio.	This was a qualitative, focus group study. All focus groups were taped and transcribed verbatim, and anonymised. The transcripts were analysed inductively, using framework analysis.	Four focus groups were carried out with 32 undergraduate medical students naïve in portfolio use. Three themes relevant to portfolio introduction emerged. The first theme was the need for clear information and support for portfolio introduction, and anxieties about how this could be supported effectively. The second was that students had negative views about reflective learning and whether this could be taught and assessed, believing formal assessment could foster socially acceptable content. The third was that participants revealed little understanding of reflective learning and its potential benefits. Rather portfolios were seen as useful for concrete purposes (e.g., job applications) not intrinsic benefits.	Undergraduate medical students without experience of portfolios are anxious about portfolio introduction. They require support in developing reflective learning skills. Care must be taken to ensure students do not see portfolios as merely yet another assessment hurdle.
58	Sánchez Gómez, S., Ostos, E. M., Solano, J. M.	2013	An electronic portfolio for quantitative assessment of	5	7	We evaluated a newly designed electronic portfolio (e-Portfolio) that provided	Our new web-based e-Portfolio was designed to evaluate surgical practical knowledge and skills targets. Students recorded	Thirty-seven of 112 students (33%) used the e-Portfolio, of which 87% reported that they understood the	Medical students reported that use of an electronic portfolio that

	and Salado, T. F.		surgical skills in undergraduate medical education			quantitative evaluation of surgical skills. Medical students at the University of Seville used the e-Portfolio on a voluntary basis for evaluation of their performance in undergraduate surgical subjects.	each activity on a form, attached evidence, and added their reflections. Students self-assessed their practical knowledge using qualitative criteria (yes/no), and graded their skills according to complexity (basic/advanced) and participation (observer/assistant/independent). A numerical value was assigned to each activity, and the values of all activities were summated to obtain the total score. The application automatically displayed quantitative feedback. We performed qualitative evaluation of the perceived usefulness of the e-Portfolio and quantitative evaluation of the targets achieved.	methodology of the portfolio. All students reported an improved understanding of their learning objectives resulting from the numerical visualization of progress, all students reported that the quantitative feedback encouraged their learning, and 79% of students felt that their teachers were more available because they were using the e-Portfolio. Only 51.3% of students reported that the reflective aspects of learning were useful. Individual students achieved a maximum of 65% of the total targets and 87% of the skills targets. The mean total score was 345 ± 38 points. For basic skills, 92% of students achieved the maximum score for participation as an independent operator, and all achieved the maximum scores for participation as an observer and assistant. For complex skills, 62% of students achieved the maximum score for participation as an independent operator, and 98% achieved the maximum scores for participation as an observer or assistant.	provided quantitative feedback on their progress was useful when the number and complexity of targets were appropriate, but not when the portfolio offered only formative evaluations based on reflection. Students felt that use of the e-Portfolio guided their learning process by indicating knowledge gaps to themselves and teachers.
59	Santonja-Medina, F., Garcia-Sanz, M. P., Martinez-Martinez, F., Bo,	2016	Portfolio as a tool to evaluate clinical competences of	10.5	-	This article investigates whether a re exive portfolio is instrumental in determining the level	A total of 131 students used the portfolio during their clinical rotation of traumatology. The students' portfolios were blind evaluated by four professors who	The reliability of the portfolio was moderate, according to the kappa index (0.48), but the evaluation scores between evaluators were	Our study suggests that the portfolio may be an important tool to quantitatively

	D. and Garcia-Estan, J.		traumatology in medical students			of acquisition of clinical competences in traumatology, a subject in the 5th year of the degree of medicine.	annotated the existence (yes/no) of 23 learning outcomes.	very similar. Considering the mean percentage, 59.8% of the students obtained all the competences established and only 13 of the 23 learning outcomes (56.5%) were fulfilled by >50% of the students.	analyze the acquisition of traumatology competences of medical students, thus allowing the implementation of methods to improve its teaching.
60	Vance, G. H. S., Burford, B., Shapiro, E. and Price, R.	2017	Longitudinal evaluation of a pilot e-portfolio-based supervision programme for final year medical students: views of students, supervisors and new graduates	6	12	Little is known about how best to implement portfolio-based learning in medical school. We evaluated the introduction of a formative e-portfolio-based supervision pilot for final year medical students by seeking views of students, supervisors and graduates on use and educational effects.	Students and supervisors were surveyed by questionnaire, with free text comments invited. Interviews were held with new graduates in their first Foundation Programme placement.	Most students used the e-portfolio (54%) and met with their supervisor (62%) 'once or twice' only. Students had more negative views: 22% agreed that the pilot was beneficial, while most supervisors thought that e-portfolio (72%) and supervision (86%) were a 'good idea'. More students reported supervision meetings benefited learning (49%) and professional development (55%) than the e-portfolio did (16%; 28%). Only 47% of students felt 'prepared' for future educational processes, though graduates noted benefits for navigating and understanding e-portfolio building and supervision. Factors limiting engagement reflected 'burden', while supervision meetings and early experience of postgraduate processes offered educational value.	Final year students have negative attitudes to a formative e-portfolio, though benefits for easing the educational transition are recognised by graduates. Measures to minimize time, repetition and redundancy of processes may encourage use. Engagement is influenced by the supervisor relationship and educational value may be best achieved by supporting supervisors to develop strategies to facilitate, and motivate self-directed learning processes in undergraduates.

61	Yielder, J. and Moir, F.	2016	Assessing the Development of Medical Students' Personal and Professional Skills by Portfolio	-	6	The introduction of a new domain of learning for Personal and Professional Skills in the medical program at the University of Auckland in New Zealand has involved the compilation of a portfolio for assessment. is departure from the traditional assessment methods predominantly used in the past has been challenging to design, introduce, and maintain as a relevant and authentic assessment method.	We present the portfolio format along with the process for its introduction and appraise the challenges, strengths, and limitations of the approach within the context of the current literature. We then outline a cyclical model of evaluation used to monitor and fine-tune the portfolio tasks and implementation process, in response to student and assessor feedback.	-	The portfolios have illustrated the level of insight, maturity, and synthesis of personal and professional qualities that students are capable of achieving. The Auckland medical program strives to foster these qualities in its students, and the portfolio provides an opportunity for students to demonstrate their re ective abilities. Moreover, the creation of a Personal and Professional Skills domain with the portfolio as its key assessment emphasizes the importance of reflective practice and personal and professional development and gives a clear message that these are fundamental longitudinal elements of the program.

62	Adeleke, O. A.<br>Cawe, B.<br>Yogeswaran, P.	2020	Opportunity for change: Undergraduate training in family medicine	-	-	The coronavirus disease 2019 (COVID-19) pandemic has changed the world as we knew it, and medical education is not an exception	Walter Sisulu University (WSU) has a distributed model of clinical training for the Bachelor of Medicine and Bachelor of Surgery (MBChB) programme. To address the challenges occasioned by the pandemic, the Department of Family Medicine and Rural Health undertook a modification of its MBChB VI programme. The changes aim to ensure the protection of all stakeholders and maintain the integrity of the programme, including the assessment	Changes were made in the delivery of the programme and in the way people interact with one another. Continuous assessment was modified, and the oral portfolio examination was introduced as the summative assessment tool	Although COVID-19 threatened the traditional way of teaching and learning, it however provided us with the opportunity to refocus and reposition our undergraduate medical programme.
63	Babovic, M.<br>Fu, R. H.<br>Monrouxe, L. V.	2019	Understanding how to enhance efficacy and effectiveness of feedback via e-portfolio: A realist synthesis protocol	-	-	The validity of feedback as one of the defining components for electronic portfolios (e-portfolios) to be effective and efficacious has yet to be demonstrated. While the literature has shown individual beneficial features of e-portfolios and feedback per se, evidence of feedback as mediated through technology directly resulting in improved educational practice is scarce. The explanation of how feedback via e-portfolio improves educational practice is particularly vague.	The aim of this research is to unpack how and why feedback via e-portfolio is likely to flourish or wither in its path. Given the complexity of intervention, we will apply a theory-driven approach for evidence synthesis called realist synthesis. Informed by realist philosophy of science, it seems the most appropriate method because it explores observed outcomes (O) in terms of causal relationship between relevant contexts (C) and generating mechanisms (M). Initial programme theory will be developed through literature scoping. Later on it will be tested against purposively gathered evidence (through database and journal search), which simultaneously will be evaluated for rigour and relevance (whether method used are trustworthy and whether data contributes to theory building). We strive to (1) uncover 'context sensitive'		

							mechanisms that generate feedback via e–portfolio to be (in) effective and (2) define in what circumstances is this mostly likely to occur.		
64	Beck Dallaghan, G. L.<br>Coplit, L.<br>Cutrer, W. B.<br>Crow, S.	2020	Medical Student Portfolios: Their Value and What You Need for Successful Implementation	-	-	Student portfolios are useful for displaying assessment evidence, enabling longitudinal tracking, and documenting student achievement. With appropriate support and coaching, online portfolios can foster student skills in self-assessment and ongoing professional development. Electronic portfolios may be integrated into the curriculum as part of a program of assessment that involves both student assessment and assessment of the educational program.			Creating a quality electronic student portfolio requires significant resources including stakeholder buy-in, dedication of time and effort from those developing the portfolio program, and technology and staff support to develop and maintain the system. Of course, all of these individuals and the information technology (IT) itself require a commitment of funding
65	Brits, H.<br>Bezuidenhout, J.<br>Van der Merwe, L. J.	2020	Quality assessment in undergraduate medical training: How to bridge the gap between what we do and what we should do	-	13	the outcome of the undergraduate medical training programme in South Africa is to produce competent medical doctors who can integrate knowledge, skills and attitudes relevant to the South African context. Training facilities have	a focus group interview was used to gather this data. The teaching and learning coordinators for five of the six modules that are offered in the clinical phase of the undergraduate medical programme participated in the focus group interview. The focus group interview proceeded as planned and took 95 minutes to complete. The responses were	the lack of formal feedback to students was identified as an area of concern; feedback plays an important role to promote student learning and improve patient care. The role of teaching and learning coordinators as drivers of quality assessment were recognized and supported. All participants agreed on	the training of assessors and the implementation of workplace-based assessment and assessment portfolios were recommended and can also address feasibility challenges. Participants

						a responsibility to ensure that they perform this assessment of competence effectively and defend the results of high-stakes assessments. This study aimed to obtain qualitative data to suggest practical recommendations on best assessment practices to address the gaps between theoretical principles that inform assessment and current assessment practices	transcribed and recorded on a matrix.	the outcome of the programme and the central role of the outcome in all assessments	recommended decreasing summative assessments and only performing these for borderline students.
66	Carney, P. A.<br>Mejicano, G. C.<br>Bumsted, T.<br>Quirk, M.	2018	Assessing learning in the adaptive curriculum	-	-	Medical education is a dynamic process that will continuously evolve to respond to changes in the foundations of medicine, the clinical practice of medicine and in health systems science. In this paper, we review how assessing learning in such a dynamic environment requires comprehensive flexible and adaptable methodological approaches designed to assess knowledge attainment and transfer, clinical skills/competency	We describe how two medical schools are approaching adaptive assessment, including using portfolio systems that encompass teaching and learning experiences while offering real-time longitudinal tracking of digital data toward improving learning and provide curricula continuous improvement cycles. Using latest technologies, portfolios produce actionable data displays with precise guidance for learning and program development.	We definitely believe that providing a flexible educational program better addresses adult learning principles, such as the learners taking responsibility for their educational achievements and bringing their own experiences to the learning process. Similarly, with increased implementation of competency-based medical education, fixed time models of training are likely to fade.	In conclusion, although individual students (from AUC study) show evidence of different levels of self-directed learning, overall, students early in their training are neophytes in applying both information generated through self-assessment and principles of self-directed learning in their clinical education. Implementing comprehensive evaluation

						development, and ethical/professional behavior. Adaptive assessments should measure the learner's ability to observe where changes in health care delivery are needed and how to implement them. Balancing formative and summative assessments will promote reflective learning so that each student will reach her/his highest potential. From the programmatic perspective, measuring the design and delivery of instruction in relation to students' efforts to achieve competency will improve learning and foster continuous professional development of faculty and advance the science of learning.			systems to foster self-assessment is feasible. Future work could focus on regulatory requirements for time-variable curricular models.
67	Chae, S. J.<br>Lee, Y. W.	2021	Exploring the strategies for successfully building e-portfolios in medical schools	8	-	The purpose of this study is to examine the characteristics of resistance among medical students toward e-portfolios and find the strategies for them to successfully prepare e-portfolios.	Participants were a group of 258 medical students. The questionnaire comprised 13 items developed based on the innovation resistance theory. The data were analyzed using descriptive analysis and Spearman's correlation analysis using PASW SPSS version 18.0 (SPSS Inc., Chicago, USA).	Students perceived that e-portfolios have a high degree of relative advantage, trialability, and complexity as their innovation resistance characteristics. Regarding perceived risk, they did not want others to see their information, but they had a high degree of demand for	The successful use of portfolios can serve as a tool for student management and assessment that can reflect their introspection, personal development, and academic

								communication with their professors.	performance. This study proposed the strategies to promote the use of e-portfolios by strengthening education on the need for them, specific feedback of instructors, and students' autonomy.
68	Cherfi, Y.<br>Szántó, K.	2019	Student portfolios: not just a tick-box exercise	-	-	Reflection is a critical aspect in any clinician's practice, and is defined as the process of creating a greater understanding of self and situation to inform future actions. The opportunity to reflect on our practice allows us to improve upon the way we approach both new and familiar situations, and helps us to become more mindful of our role within the multidisciplinary team.		Although there are many positives in having our portfolios designed this way, there are some challenges that we encountered through having used them for an extended length of time. As a result of the reflective nature of these entries, they require good 'interpreters' in the form of senior clinicians who can use them as opportunities for dialogue rather than view them as mandatory tasks to complete. As tools of assessment, portfolios therefore need to be subjected to rigorous appraisal and quality assurance. We found that it benefitted our learning more when clinicians took time to address our concerns and counsel us through emotional situations, and this has been reflected in the literature. More often than not, however, the feedback that we received was	In conclusion, our experience with portfolios has been broadly positive as they ensure that students are able to further explore the psychosocial aspects of medical training. Although not without their limitations and potential for improvement, they are a tool that has aided us in recognising our own strengths and weaknesses, and allows us to focus on achieving more personalised, goal- oriented clinical placements.

								superficial, lacked tailoring to the individual and did not provide enough opportunity to critique ourselves as developing clinicians. This has been echoed in the conclusion of a large systematic review, emphasising the need for enthusiastic pastoral support	
69	Chu, A.<br>Biancarelli, D.<br>Drainoni, M. L.<br>Liu, J. H.<br>Schneider, J. I.<br>Sullivan, R.<br>Sheng, A. Y.	2019	Usability of Learning Moment: Features of an E-learning Tool That Maximize Adoption by Students	8	21	E-learning is widely used in medical education. To maximize the potential of E-learning tools, every effort should be made to encourage adoption by optimizing usability. We created Learning Moment (LM), a web-based application that integrates principles of asynchronous learning and learning portfolios into a platform on which students can document and share learning experiences that occur during clinical work. We sought to evaluate the usability of LM and identify features that optimize adoption by users	We implemented LM in August 2016 at a busy, urban, tertiary care emergency department that hosts an emergency medicine residency, robust third and fourth year medical student clerkships as well as a physician assistant student rotation. We conducted a single-center, mix-methods study using the System Usability Scale (SUS) questionnaire and qualitative interviews. We sent e-mail invitations with subsequent reminders to all students who rotated in our emergency medicine clerkship from August 2016 to April 2017 to complete the SUS questionnaire anonymously and to participate in qualitative interviews. We employed purposive sampling to recruit students who used LM during their rotation to participate in our qualitative interviews. We conducted semi-structured interviews with 13 participants (10 individual interviews and one 3-person group interview) between January and March 2017 using an ethnographic approach and utilized a general	Thirty of the seventy students invited to participate completed the SUS questionnaire (Response rate of 42.8%). The mean SUS score is 80.9 (SD 18.2, 80% CI 76.5 – 85.3). The internal consistency of the responses achieved the Cronbach's Alpha of 0.95. The participants stressed the importance of the following in the adoption of LM: maximal simplicity and usability, compatibility with learning preferences, and department-wide acceptance and integration	The overall perceived usability of LM was high. Our qualitative data revealed important implications for future designers to maximize adoption: include target users in every step of the design and development process to maximize simplicity and usability; build features that cater to a diversity of learning preferences; involve the entire department and find ways to incorporate the tool into the educational infrastructure and daily workflow

							inductive method to analyze and code for potential themes.		
70	Cunningham, H.<br>Taylor, D. S.<br>Desai, U. A.<br>Ender, K. L.<br>Glickstein, J.<br>Krishnan, U. S.<br>Richards, B. F.<br>Charon, R.<br>Balmer, D. F.	2020	Reading the Self: Medical Students' Experience of Reflecting on Their Writing Over Time	-	21	To investigate students' experience (over time) with meta-reflection writing exercises, called Signature Reflections. These exercises were used to strengthen reflective capacity, as part of a 4-year reflective writing portfolio curriculum that builds on a recognized strategy for reflection (narrative medicine) and employs longitudinal faculty–mentors.	In 2018, the authors conducted 5 focus groups with 18 third-year students from the Columbia University Vagelos College of Physicians and Surgeons class of 2019 to examine students' experience with Signature Reflections. Using an iterative, thematic approach, they developed codes to reflect common patterns in the transcripts, distilled conceptually similar codes, and assembled the code categories into themes.	Three core themes (safe space, narrative experience, mirror of self) and 1 overarching theme (moving through time) were identified. Students frequently experienced relief at having a safe reflective space that promoted grappling with their fears or vulnerabilities and highlighted contextual factors (e.g., trusted faculty–mentors, protected time) that fostered a safe space for reflection and exploration. They often emphasized the value of tangible documentation of their medical school journey (narrative experience) and reported using Signature Reflections to examine their emerging identity (mirror of self). Overlapping with the core themes was a deep appreciation for the temporal perspective facilitated by the Signature Reflections (moving through time).	A longitudinal narrative medicine based portfolio curriculum with pauses for meta-reflection allowed students, with faculty support, to observe their trajectory through medical school, explore fears and vulnerabilities, and narrate their own growth. Findings suggest that narrative medicine curricula should be required and sufficiently longitudinal to facilitate opportunities to practice the skill of writing for insight, foster relationships with faculty, and strengthen students' temporal perspectives of their development.
71	Datta, R.<br>Datta, K.<br>Routh, D.<br>Bhatia, J. K.<br>Yadav, A. K.<br>Singhal, A.<br>Dalal, S. S.	2021	Development of a portfolio framework for implementation of an outcomes-based healthcare professional education	6.5	10	The explicit declaration of Entrustable Professional Activities (EPA) and milestones are an essential component of a competency based	Amodified e-Delphi method was used after incorporation of a study group of medical education experts (MEDEX-G). Consensus was defined as 75% agreement. Both qualitative and quantitative data was collected and analysed to conduct three rounds of the	The MEDEX-G consensus template is an important development to encourage the adoption of portfolios in a competency based medical education curriculum. It can be freely adopted by various	

			curriculum using a modified e-Delphi method			medical education curricula. The present study attempts to develop a portfolio framework to document them for adaptation in any healthcare professional education curriculum development.	Delphi. Results: The draft template was prepared by the core faculty of medical education centre. The final template was approved by the experts after 03 iterations of anonymous online voting and presentation of summary results by the moderator. The final template lists out the milestones of each EPA separately with provision for expected expertise and level, suggestive teaching learning activities and assessments, reflections by the students and feedback by facilitator for each EPA	healthcare professional education bodies in various disciplines.	
72	Désilets, V. Graillon, A. Ouellet, K. Xhignesse, M. St-Onge, C.	2021	Reflecting on professional identity in undergraduate medical education: implementation of a novel longitudinal course	6.5	13	Today's healthcare professionals face numerous challenges. Improving reflection skills has the potential to contribute to the better management of complex patients and healthcare systems, as well as to improve professional practice. However, the question of how reflection skills can inform professional identity development at the undergraduate medical education level remains unanswered.	The authors developed and implemented a 4-year course that aims to engage students in a reflective process to increase their awareness of their professional identity development. The course is structured around three types of pedagogical activities: workshops, reflections deposited in an electronic portfolio, and individual discussions with mentors.	Sixty-four 1st year students (33%) and 17 mentors (50%) from the 2017–2018 cohort completed evaluation questionnaires. For the 2018–2019 academic year, 73 1st year students (34%) and 27 2nd year students (14%), as well as 20 1st year (59%) and 19 2nd year mentors (57%) replied. Students and mentors considered that the pedagogical activities contributed to the development of students' professional identity through the acquisition of reflection skills, but some elements were perceived as challenging, notably, completing the portfolio, finding a subject to reflect about and the timing of the proposed activities.	An important preoccupation when wanting to foster the development of professional identity through the acquisition of reflection skills is the authenticity of students' reflection. We tried to favor authentic reflection, by having a mentee-mentor pair throughout the entire 4-year course. A rigorous evaluation process helped us identify and promptly correct

									issues as they surfaced.
73	Franco, R.<br>Ament Giuliani Franco, C.<br>de Carvalho Filho, M. A.<br>Severo, M.<br>Amelia Ferreira, M.	2020	Use of portfolios in teaching communication skills and professionalism for Portuguese-speaking medical students	8.5	11	This study aimed to analyse the effect of a portfolio with three activities fostering students' reflection, selfefficacy and teaching of communication skills and professionalism.	A cross-sectional study was applied with a sample of third- and fourth-year medical students in one Portuguese and three Brazilian universities. A three-activity portfolio (course evaluation and learning, self-efficacy activity and free reflective writing) was used during a two-month course on communication skills and professionalism. The 69 students enrolled in the course were invited to complete the three-activity portfolio via Likert-type questionnaires, open-ended questions and narrative. Content and lexical analysis and the Reflection Evaluation for Learners' Enhanced Competencies Tool (REFLECT) were used for assessing the qualitative data. The questionnaires were evaluated using principal components analysis and Cronbach's α. Pearson's correlation was applied to portfolio activities.	Of the 69 participants, 85.5% completed at least one activity. Reflecting on what they learned in the communication module, the students did not mention professionalism themes. In the self-efficacy activity on communication, 25% of the fragments were related to professionalism themes. There was a negative correlation between students' self-efficacy and the REFLECT rubric score ($r(19)=-0.744$; $p< 0.0001$).	Teachers must consider the activity's influence on the reflections when assessing the portfolio. This model of a three-activity portfolio provided diverse ways of encouraging and assessing reflections, supporting teaching improvement and adaptation, evaluating students' self-efficacy and showing that students' higher reflective capacity may promote feelings of low effectiveness.
74	Imafuku, R.<br>Saiki, T.<br>Hayakawa, K.<br>Sakashita, K.<br>Suzuki, Y.	2021	Rewarding journeys: exploring medical students' learning experiences in international electives	-	15	International electives are recognized as a high-impact practice in clinical education. However, medical students' actual learning experiences during electives have not been explored fully	Specifically, drawing on language management theory, this exploratory case study investigates students' perceived learning outcomes and the managing processes by which they gained benefits from cross-cultural learning experiences in international electives. Written reflective reports in a series of e-portfolios were collected from 43 Japanese medical students who	During the programme, they experienced linguistic, sociolinguistic, and sociocultural difficulties, and attempted to overcome them by employing various adjustment strategies, such as meaning-focused coping, social relationship building, management of learning opportunities, communication	Since the combination of several strategies was needed depending on the situation, the management process is a context-dependent and complex phenomenon. The findings in this

							participated in a four-week international elective. Moreover, to further explore their emotions and the reasons behind adopting a particular adjustment behaviour, follow-up interviews with 12 students were undertaken soon after they returned home. Using reflexive thematic analysis, the qualitative data were analysed. Their perceived learning outcomes were categorized into seven themes: medical knowledge and skills, communication, career management and development, international healthcare, society and culture, medical education, and personal development	management, and developing approaches to learning. Managing problems in academic contact situations is not a linear process; it is iterative and cyclical.	study provide new insights into student participation in short-term international elective programmes in order to develop academic and social support strategies for educators at both home and host institutions.
75	Kennedy, G. Rea, J. N. M. Rea, I. M.	2019	Prompting medical students to self-assess their learning needs during the ageing and health module: a mixed methods study	6.5	18	Understanding our learning needs is fundamental for safe, effective and knowledge-based medical practice and facilitates life-long learning.	A mixed methods study investigated fourth- year medical students' self-perceived understanding of their learning needs using 1] a visual scale, before and after a four-week module in Ageing and Health (A&H) and 2] through focus group discussions. During 2013–14 academic year, all students (252) were invited to use a Visual Analogue Scale (VAS) tool to self-assess their learning needs that were linked to Ageing and Health curriculum learning outcomes. Assenting students (197 at pre-self-assessment, 201 at post-assessment) returned anonymous Visual Analogue Scales, self-assessing history-taking skills, examination skills, knowledge of medication use, co-morbidity, nutritional and	The VAS responses increased for each curriculum domain with significant differences between the pre-and post responses – for the student-year-group. Nutritional and swallowing knowledge showed the greatest improvement from a self-assessed low baseline at entry. Focus-group students generally viewed the VAS tool positively, and as an aid for prompting consideration of current and future clinical practice. Some students recognised that 'a need to be ready-for-work' focused engaged learning; others demonstrated self-regulated learning through self-motivation and an action	

							swallowing assessment responses, before and after the A&H module. Three student focus groups explored whether completion of the VAS self-assessment had prompted improved self-awareness of their learning needs.	plan. The Visual Analogue Scale quantitative responses showed increased student-self-perceived learning for each curriculum domain at fourth-year completion of the A&H module, suggesting that prompting self-assessment had increased students' knowledge and skills. Focus group students saw the VAS tool as useful for prompting awareness of their current and future learning needs.	
76	Mejicano, G. C. Bumsted, T. N.	2018	Describing the Journey and Lessons Learned Implementing a Competency-Based, Time-Variable Undergraduate Medical Education Curriculum	-	-	Oregon Health & Science University School of Medicine launched a completely new undergraduate medical education curriculum in 2014. This initiative dramatically transformed the MD degree program, changing the instructional content taught, the pedagogical methods used by the faculty, and the methods of assessment, and it added new elements such as academic coaching and programmatic entrustment to the program. One of the most exciting and impactful aspects to date of this curricular	This article describes the school's progress to date in its curricular transformation and articulates lessons learned thus far in driving substantive and dramatic institutional changes that profoundly impact students, faculty, and administrators in one academic health center.		

						transformation has been the deliberate implementation of a competency-based framework that incorporates frequent assessment, tracking of student progression using an electronic portfolio, and academic coaching to optimize learning and customize curricular elements for each student. The next major step in this process—the implementation of time-variable progression—is currently ongoing as a planning group at the school works through the conceptual, logistical, legal, and regulatory issues related to implementing such a system. When implementation is complete, MD students will graduate only once they have earned entrustment for all 13 Core Entrustable Professional Activities for Entering Residency.			
77	Oudkerk Pool, A.<br>Jaarsma, A. D.	2020	Student perspectives on competency-	-	18	Portfolio-based assessments require that learners'	Students uploaded performance data in a competency-based portfolio. During one clerkship	Portfolios provide an accurate but fragmented picture of student	This study confirms the importance of

	C.<br>Driessen, E. W.<br>Govaerts, M. J. B.		based portfolios: Does a portfolio reflect their competence development?			competence development is adequately reflected in portfolio documentation. This study explored how students select and document performance data in their portfolios and how they perceive these data to be representative for their competence development.	period, twelve students also recorded an audio diary in which they reflected on experiences and feedback that they perceived to be indicants of their compe- tence development. Afterwards, these students were interviewed to explore the extent to which the perfor- mance documentation in the portfolio corresponded with what they considered illustrative evidence of their development. The interviews were analyzed using thematic analysis.	development. Portfolio documentation was influenced by tensions between learning and assessment, student beliefs about the goal of portfolios, student performance evaluation strategies, the learning environment and portfolio structure.	taking student perceptions into account when implementing a competency-based portfolio. Students would benefit from coaching on how to select meaningful experiences and performance data for documentation in their portfolios. Flexibility in portfolio structure and requirements is essential to ensure optimal fit between students' experienced competence development and portfolio content.
78	Roskvist, R.<br>Eggleton, K.<br>Goodyear-Smith, F.	2020	Provision of e-learning programmes to replace undergraduate medical students' clinical general practice attachments during COVID-19 stand-down	-	-	Senior medical students at the University of Auckland, New Zealand spend most of their learning time in clinical attachments. Experiential apprentice-style training is traditionally recognised as an important aspect of obtaining competency. In March 2020 they were stood down from their general practice placements in the	This paper describes the steps taken and the underlying theoretical foundations for our expediently developed online course.	Our online learning programme has three online components, reflecting the domains of educational environment theory: asynchronous discussion forums; a symposium facilitating social interactions and teacher presence, and a portfolio facilitating personal goal aspects. The latter is underpinned by a multi-theories model of adult learning, built upon the scaffolding framework that supports our entire medical curriculum. Within this	It is hoped that the mechanisms described here might be useful to other educators facing similar challenges.

						context of a national response to the COVID-19 pandemic. Acute conversion of their general practice education from experiential clinical exposure to online and offsite learning was required.		theory, we propose a five-stage model of learning. Learning from this experience contributes to the body of knowledge around online education, particularly in meeting the needs of a clinical attachment traditionally grounded in experiential learning.	
79	Santonja-Medina, F.<br>García-Sanz, M. P.<br>Santonja-Renedo, S.<br>García-Estañ, J.	2018	Mismatch between student and tutor evaluation of training needs: a study of traumatology rotations	8	-	The adoption of a system based on the acquisition of clinical competences [1] and the adoption of assessment instruments such as OSCEs in medical schools [2] is slowly changing the way medical teaching is carried out. Clinical competences relative to clinical skills and procedures require different teaching and learning mechanisms as well as, more importantly, different methods of ascertaining whether or not they have been fully acquired. Learning these clinical skills requires time, and procedures need to be repeated more than once before teachers can decide whether or not students are capable	An e-portfolio was used to determine the optimal number of times students need to repeat a procedure before they are fully capable of performing it without supervision. The results were compared with the actual number of repetitions performed during the internship period. We also asked these students and their teachers about the optimal number of times each skill should be repeated before it could be considered fully acquired. The questionnaire was answered by 98.6% of the students and 70.3% of their teachers.	Both students and teachers agreed on a similar optimal value for 16 out of the 21 clinical procedures selected; in the remaining 5, teachers thought that students needed to repeat the procedure more times than the number stated by students. When these optimal values were compared with the actual values recorded in the portfolio during the internships, it was found that about half of all clinical procedures were carried out fewer times than expected, thus providing important feedback about the rotation-based training process.	Quantitative information collected in the portfolios revealed a moderate mismatch between students' and teachers' perceptions of training needs

						of performing them autonomously, or in other words, whether the required learning outcome has been achieved [3, 4]. However, determining the optimal number of times that students need to repeat a procedure in order to become competent enough to perform it independently remains a challenge in undergraduate medical education.			
80	Sohrmann, M. Berendonk, C. Nendaz, M. Bonvin, R. Swiss Working Group For Profiles, Implementation	2020	Nationwide introduction of a new competency framework for undergraduate medical curricula: a collaborative approach	-	-	Switzerland recently introduced PROFILES, a revised version of its national outcomes reference framework for the undergraduate medical curriculum. PROFILES is based on a set of competencies adapted from the CanMEDS framework and nine entrustable professional activities (EPAs) that students have to be able to perform autonomously in the context of a predefined list of clinical sit- uations. The nationwide implementation of such a competency- and EPA-based	The vice-deans for education mandated a Swiss Working Group for PROFILES Implementation (SWGPI) to elaborate a guide presenting the principles and best practices based on the current scientific literature, to ensure the co- herence between the future developments of the medical curricula and the evolution of the FLE, and to propose a coordinated research agenda to evaluate the implementation process.	On the basis of the literature and analysis of our national context, we determined the key elements important for a successful implementation. They can be grouped into several areas including curricular design and governance, the assessment system and entrustment process, faculty development and change management. We also identified two dimensions that will be of particular importance to cre- ate synergies and facilitate exchange between the medical schools: a systematic approach to curriculum mapping and the longitudinal integration of an e-portfolio to support the student learning process.	The nationwide collaborative approach to define strategies and conditions for the implementation of a new reference framework has allowed to develop a shared understanding of the implications of PROFILES, to promote the establishment of Swiss mapping and e-portfolio communities, and to establish the conditions necessary for ensuring the continuous alignment of the FLE with the

						approach to medical education is a complex process that represents an important change to the organisation of undergraduate training in the various medical schools. At the same time, the concepts underly- ing PROFILES also have to be reflected at the level of the Federal Licencing Examination (FLE) and the national accreditation process.			evolving medical curricula.
81	Souza, A. D. Vaswani, V.	2020	Diversity in approach to teaching and assessing ethics education for medical undergraduates: A scoping review	-	8	There are diverse methods to teach medical ethics, and there is no single accepted approach towards its learning and assessment. The authors aim to explore the various strategies practised to teach undergraduate medical students the fundamentals of medical ethics and their evaluation. The authors reviewed the articles published from January 2014 to September 2019.	The authors searched PubMed for the relevant publications and extracted the information using a data extraction sheet. Twenty-nine articles were included for the review, which fulfilled the inclusion criteria.	Case-based discussions were a widely accepted strategy to learn ethics. The studies highlighted a mixed teaching approach using multiple teaching tools. A qualitative approach was preferred for the assessment through reflections, simulated patient interactions, and development of portfolios. However, there are gaps in the existing literature on the assessment strategies for ethics education. Heterogeneity still exists in the planning of the curricula, teaching, and assessment methods. These curricula suit the cultural and religious set up of that particular country.	Case-based discussion is a popular teaching strategy, and there exist numerous innovative and cost-effective active teaching strategies. There is a need for studies that are more rigorous to address the evaluation of the ethics curricula. This review would help educators to choose their preferred approach based on their teaching environment.
82	Ten Cate, O. Graafmans, L.	2018	The EPA-based Utrecht	-	-	As reports of the application of	In a three-year process, the UMC Utrecht Curriculum Committee	In the resulting curriculum, operational from 2016,	The framework of EPAs went

	Posthumus, I. Welink, L. van Dijk, M.		undergraduate clinical curriculum: Development and implementation			entrustable professional activities (EPAs) increase, not only for postgraduate but also for undergraduate medical education, there is a need for descriptions of what a UME curriculum with EPAs could look like. We provide such a description based on the experiences at University Medical Center Utrecht, the Netherlands, which can be used as an example by other curriculum developers.	developed a clinical workplace curriculum with an EPA structure, taking into account examples, such as the US Core EPAs for Entering Residency, and recommendations to integrate and increase the length of clerkships.	students train to be trusted with indirect supervision before graduation in five broad EPAs: the clinical consultation; general medical procedures; informing, advising and guiding patients and families; communicating and collaborating with colleagues; and extraordinary patient care. Each of these integrates smaller (nested) EPAs that receive focused training attention in integrated clerkships at various moments and must be signed off for entrustment with indirect supervision to complete the clerkship.	through many iterations before it was consolidated. Among the issues that required special attention was the application of a supervision levels scale for sign-off, the necessity to cover all relevant clinical content while not labeling too many small tasks each as a separate EPA, methods of EPA-focused assessment in the workplace and the creation of an e-portfolio model to serve assessment and entrustment.
83	Yoo, D. M. Cho, A. R. Kim, S.	2019	Evaluation of a portfolio-based course on self-development for pre-medical students in Korea	7.5	-	We have developed and operated a portfolio-based course aimed at strengthening pre-medical students' capabilities for self-management and self-improvement. In order to determine the effectiveness of the course and to establish future operational strategies, we evaluated the course and the	The subjects of this study were 97 students of a pre-medical course "Self-development and portfolio I" in 2019. Their learning experience was evaluated through the professor's assessment of portfolios they had submitted, and the program was evaluated based on the responses of 68 students who completed a survey. The survey questionnaire included 32 items. Descriptive statistics were reported for quantitative data, including the mean and standard deviation. Opinions collected	The evaluation of students' portfolios showed that only 6.2% of the students' portfolios were well-organized, with specific goals, strategies, processes, and self-reflections, while most lacked the basic components of a portfolio (46.4%) or contained insufficient content (47.4%). Students' responses to the survey showed that regular portfolio personality assessments (72.1%), team (64.7%), and individual (60.3%) activities were felt to	The findings of this study suggest that standardized samples, guidelines, and sufficient time for autonomous portfolio creation should be provided. In addition, education on portfolio utilization should be conducted in small groups in the future.

						students' learning experience.	from the open-ended question were grouped into categories.	be more appropriate as educational methods for this course, rather than lectures. Turning to the portfolio creation experience, the forms and components of the portfolios (68.2%) and the materials provided (62.2%) were felt to be appropriate. However, students felt that individual autonomy needed to be reflected more (66.7%) and that this course interfered with other studies (42.5%).	
84	Yoo, D. M.<br>Cho, A. R.<br>Kim, S.	2020	Development and validation of a portfolio assessment system for medical schools in Korea	7.5	-	Consistent evaluation procedures based on objective and rational standards are essential for the sustainability of portfolio-based education, which has been widely introduced in medical education. We aimed to develop and implement a portfolio assess- ment system, and to assess its validity and reliability.	We developed a portfolio assessment system from March 2019 to August 2019 and confirmed its content validity through expert assessment by an expert group comprising 2 medical education specialists, 2 professors involved in education at medical school, and a professor of basic medical science. Six trained assessors conducted 2 rounds of evaluation of 7 randomly selected portfolios for the "Self-Development and Portfolio II" course from January 2020 to July 2020. These data are used inter-rater reliability was evaluated using intra-class correlation coefficients (ICCs) in September 2020.	The portfolio assessment system is based on the following process; assessor selection, training, analytical/comprehensive evaluation, and consensus. Appropriately trained assessors evaluated portfolios based on specific assessment criteria and a rubric for as- signing points. In the analysis of inter-rater reliability, the first round of evaluation grades was submitted, and all assessment areas except "goal-setting" showed a high ICC of 0.81 or higher. After the first round of assessment, we attempted to standardize objective assess- ment procedures. As a result, all components of the assessments showed close correlations, with ICCs of 0.81 or higher.	We confirmed that when assessors with an appropriate training conduct portfolio assessment based on specified standards through a systematic procedure, the results are reliable.

85	Kim, J. W.<br>Ryu, H.<br>Park, J. B.<br>Moon, S. H.<br>Myung, S. J.<br>Park, W. B.<br>Yim, J. J.<br>Yoon, H. B.	2020	Establishing a Patient-centered Longitudinal Integrated Clerkship: Early Results from a Single Institution	7	-	Longitudinal integrated clerkships (LICs) have been adopted by medical schools to overcome the limitations of traditional block clerkship rotations and to promote continuity of care. In 2018, Seoul National University College of Medicine introduced a patient-centered LIC program as part of a new curriculum in parallel with traditional block rotation clerkships. The purpose of this study was to present the patient-centered LIC program and to investigate its educational effects.	In 2018 and 2019, a total of 298 third-year medical students participated in the LIC program. We divided the students into groups of eight, which were organized into corresponding discussion classes. Throughout the academic year, students followed up patients by interviewing them at the hospital or reviewing their electric medical records. Discussion classes on set topics were held seven times per year with facilitators and clinical faculties. Students completed a course evaluation questionnaire at the end of the academic year. The questionnaire included 22 items measured on a 5-point scale and two open-ended questions asking about the bene ts and limitations of the program. The items covered three domains: student experience, satisfaction, and self-assessment. Final reflective essays were collected as both student assessments and data for qualitative analysis.	During the study period, the overall experience of the students improved. We increased the number of faculty members and patients and decreased the number of students in each discussion class. We also provided additional feedback through an e-portfolio. Students' satisfaction changed positively. Compared to the rotational clerkship, students answered that the LIC provided additional help in learning the two core competencies. During the first 2 years of the program, the percentage of students who answered that the program was more helpful than the rotational clerkship increased from 23.7% to 46.4% for continuity of care ($P < 0.001$), and from 20.5% to 50.7% for patient-centered care ($P < 0.001$).	Our patient-centered LIC, in parallel with traditional block rotation clerkships, had a positive effect on students' experience of continuity of care and patient-centered care.
86	Zhou, Y. C.<br>Tan, S. R.<br>Tan, C. G. H.<br>Ng, M. S. P.<br>Lim, K. H.<br>Tan, L. H. E.<br>Ong, Y. T.<br>Cheong, C. W. S.<br>Chin, A. M. C.<br>Chiam, M.<br>Chia, E. W. Y.	2021	A systematic scoping review of approaches to teaching and assessing empathy in medicine	-	8	Empathy is pivotal to effective clinical care. Yet, the art of nurturing and assessing empathy in medical schools is rarely consistent and poorly studied. To inform future design of programs aimed at nurturing empathy in medical students and	This systematic scoping review (SSR) employs a novel approach called the Systematic Evidence Based Approach (SEBA) to enhance the reproducibility and transparency of the process. This 6-stage SSR in SEBA involved three teams of independent researchers who reviewed eight bibliographic and grey literature databases and performed	In total, 24429 abstracts were identified, 1188 reviewed, and 136 included for analysis. Thematic and content analysis revealed five similar themes/categories. These comprised the 1) definition of empathy, 2) approaches to nurturing empathy, 3) methods to assessing empathy, 4) outcome	Nurturing empathy in medicine occurs in stages, thus underlining the need for it to be integrated into a formal program built around a spiralled curriculum. We forward a framework built

	Lim, C.<br>Wijaya, L.<br>Chowdhury, A. R.<br>Kwek, J. W.<br>Fong, W.<br>Somasundaram, N.<br>Ong, E. K.<br>Mason, S.<br>Krishna, L. K. R.					doctors, a review is proposed.	concurrent thematic and content analysis to evaluate the data.	measures, and 5) enablers/barriers to a successful curriculum.	upon these stages and focus attention on effective assessments at each stage of the program. Tellingly, there is also a clear need to consider the link between nurturing empathy and one's professional identity formation. This foregrounds the need for more effective tools to assess empathy and to better understand their role in longitudinal and portfolio based learning programs.
87	King, T. S.<br>Sharma, R.<br>Jackson, J.<br>Fiebelkorn, K. R.	2019	Clinical Case-Based Image Portfolios in Medical Histopathology	7	-	This descriptive article describes the use of clinical case-based portfolios in histopathology teaching laboratories in conjunction with virtual microscopy not only to integrate histology and pathology disciplines for first and second year medical students but also to stimulate student engagement, promote self-directed and group-based learning and enhance	Portfolios consisted of PowerPoint files encompassing four to five clinical case studies relevant to the topics cov- ered that week. Portfolios integrated study materials provided in the module-specific lec- tures, clinical skill lectures, and online interactive content. Two sets of portfolios, Individual and Group, were used. Individual Portfolios were completed by each student and uploaded prior to the laboratory session. Group Portfolios were completed by stu- dents working together in small groups during the laboratory session with minimal faculty	Both first- and second-year students agreed that the use of portfolios in conjunction with virtual microscopy promoted understanding and encouraged discussion of the topics covered during the week and that group members worked well together and contributed to the completion of the portfolios. Performances on the Histology and Cell Biology and Pathology sections on the United States Medical Licensing ExaminationVR (USMLEVR) remained	Overall, use of portfolios promoted peer teaching and contributed towards successful transition to the new system-based integrated curriculum with continued strong performance on the USMLE.

						student-to-student interaction in a structured manner.	assistance. The functional utility and acceptance of Individual and Group Portfolios among first- and second-year medical students was evaluated using electronic surveys and examination performances.	consistent and in line with national averages.	
88	Montrezor, L. H.	2021	Lectures and collaborative working improves the performance of medical students	9.5	-	The teaching-learning process is complex and requires continuous research and dedication. Research has been important to prove that student achievement can improve when active methodologies are properly implemented, either in isolation or integrated with a short lecture.	The purpose of this work was to evaluate the performance and perception of first-year medical stu- dents subjected to a protocol involving the integration of a short lecture and collaborative compilation of portfolios on the topic of digestive physiology. After the lectures and elaboration of the portfolios, the students completed two tests: cognitive monitor- ing test (CMT) and integrative cognitive test (ICT), and then, they gave their opinions of the strategy. For CMT, the percentage of grade exceeding 7.0 was higher for the group who performed the portfolio activity, compared with the group who did not under- take the activity, and there was a lower percentage of incorrect answers among the portfolio group students, compared with the group that did not perform the activity.	For ICT, the percentage of grades exceeding 7.0 was higher for the students who used the portfolio, compared with those who did not perform the activity, and there was also a reduction in incorrect answers among students who performed the portfolio activity, compared with those who did not perform the activity.	The combination of short lectures and collaborative group work using a portfolio improved the grades obtained for digestive physiology. Most of the students believed that the collaborative work contributed to their learning about digestive physiology. In addition, most of them were comfortable working in their groups and did not feel dominated by other group members.
89	Pinto-Powell, R. Lahey, T.	2019	Just a Game: the Dangers of Quantifying Medical Student Professionalism	-	-	A medical student on her internal medicine clerkship says her numerical medical professionalism grade was "just a game".	Building on this anecdote, we suggest there is good reason to believe that numerical summative assessments of medical student professionalism can, paradoxically, undermine medical student professionalism by sapping internal motivation and converting conversations about		We suggest better ways of supporting medical student professional development, including a portfolio comprised of

							core professional values into just another hurdle to residency.		written personal reflection and periodic 360° formative assessment in the context of longitudinal faculty coaching.
90	Royce, C. S.<br>Everett, E. N.<br>Craig, L. B.<br>Fleming, A.<br>Forstein, D. A.<br>Graziano, S. C.<br>Hampton, B. S.<br>Hopkins, L.<br>McKenzie, M. L.<br>Morgan, H. K.<br>Sims, S. M.<br>Morosky, C.	2021	To the Point: advising students applying to Obstetrics and Gynecology residency in 2020 and beyond	-	-	This article, from the “To the Point” series by the Undergraduate Medical Edu- cation Committee of the Association of Professors of Gynecology and Obstetrics, is a guide for advising medical students applying to Obstetrics and Gynecology residency programs. The residency application process is changing rapidly in response to an increasingly complex and competitive atmosphere, with a wider recognition of the stress, expense, and difficulty of matching into graduate training programs. The coronavirus disease 2019 pandemic and societal upheaval make this application cycle more challenging than ever before. Medical	The authors outline a model for faculty career advisors, distinct from mentors or general academic advisors. The faculty career advisor has detailed knowledge about the field, an in-depth understanding of the application process, and what constitutes a strong application. The faculty career advisor provides accurate information regarding residency programs within the specialty, helping students to strategically apply to programs where the student is likely to match, decreasing anxiety, expense, and overapplication. Faculty career advisor teams advise students throughout the application process with periodic review of student portfolios and are available for support and advice throughout the process. The authors provide a guide for the faculty career advisor in Obstetrics and Gynecology, including faculty development and quality improvement.	-	-

						students need reliable, accurate, and honest advising from the faculty in their field of choice to apply successfully to residency.			
91	Shiozawa, T.<br>Glauben, M.<br>Banzhaf, M.<br>Griewatz, J.<br>Hirt, B.<br>Zipfel, S.<br>Lammerding-Koeppel, M.<br>Herrmann-Werner, A.	2020	An Insight into Professional Identity Formation: Qualitative Analyses of Two Reflection Interventions During the Dissection Course	8	21	The professional behavior of future doctors is increasingly important in medical education. One of the first subjects in the curriculum to address this issue is gross anatomy. The Tuebingen Medical Faculty implemented a learning portfolio and a seminar on medical professionalism during the dissection course. The aims of this research project are to get an overview of how students form a professional identity in the dissection course and to compare the content of both their oral and written reflections on the course.	A qualitative analysis was conducted of the oral and written reflections on the dissection laboratory experience. This study was conducted during winter term 2013/2014 with a cohort of 163 participants in the regular dissection course. Written reflection texts (from n = 96 students) and audio recordings from four oral reflection seminar discussions (with n = 11 students) were transcribed and deductively categorized with Mayring's qualitative content analysis method.	Both qualitative analyses show that students reflected on many topics relevant to professional development, including empathy, respect, altruism, compassion, teamwork, and self-regulation. Quantitative analysis reveals that students who attended the oral reflection wrote significantly more in their written reflection than students who did not. There is, however, no difference in the reflection categories. Reflection content from students corresponds with categories derived from existing competency frameworks.	Both the seminar (oral reflections) and the learning portfolio (written reflections) present excellent opportunities to foster professional development during anatomy education; the key is using them in conjunction with the dissection course.
92	Forenc, K. M.<br>Eriksson, F. M.<br>Malhotra, B.	2020	Medical Students' Perspectives on an Assessment of Reflective Portfolios [Letter]	-	-				
93	Heeneman, S.<br>Driessen, E.<br>Durning, S. J.<br>Torre, D.	2019	Use of an e-portfolio mapping tool: connecting	-	-	Reflective practice is an important characteristic of a knowledgeable health	The purpose of this article is to demonstrate the design and rationale of e-portfolio mapping and how this tool could be used	Following the stepwise approach typical for the reflective cycle, two types of maps were designed, a	The e-portfolio mapping tool can be a useful and supportive tool to

			experiences, analysis and action by learners			professional. Reflection is needed to maintain professional competence, practice-based learning, and an improvement focused attitude. Yet, learners struggle with reflective practice activities as they are perceived as challenging and time consuming. We used the idea of concept mapping to design an e-portfolio tool that supports learners with their reflective practice activities.	to support reflective practice activities.	trigger map and a competency map. In a trigger map, the learner reflects on a concrete learning or feedback experience. In a competency map the learner interprets and synthesizes several previous trigger maps which ultimately leads to the formulation of new learning objectives.	foster learners' reflective skills and provide mentors with in-depth insight into the students' learning and reflection processes.
94	Kanfi, A.<br>Faykus, M. W.<br>Tobler, J.<br>Dallaghan, G. L. B.<br>England, E.<br>Jordan, S. G.	2021	The Early Bird Gets the Work: Maintaining a Longitudinal Learner Portfolio From Medical School to Physician Practice	-	-				
95	Kassab, S. E.<br>Bidmos, M.<br>Nomikos, M.<br>Daher-Nashif, S.<br>Kane, T.<br>Sarangi, S.<br>Abu-Hijleh, M.	2020	Construct Validity of an Instrument for Assessment of Reflective Writing-Based Portfolios of Medical Students	11	-	Assessment of reflective writing for medical students is challenging, and there is lack of an available instrument with good psychometric properties. The authors developed a new instrument for assessment of reflective writing-based portfolios and	After an extensive literature review and pilot testing of the instrument, two raters assessed the reflective writing-based portfolios from years 2 and 3 medical students (n=135) on three occasions. The instrument consists of three criteria: organization, description of an experience and reflection on the experience. We calculated the reliability of scores using generalizability theory with a fully	The dependability (Φ) coefficient of the portfolio scores was 0.75 using two raters on three occasions. Students' portfolio scores represented 46.6% of the total variance across all score comparisons. The variance due to occasions was negligible, while the student– occasion interaction was small. The variance due to student–	We demonstrated the presence of different sources of evidence that support construct validity of the study instrument. Further studies are warranted before utilizing this instrument for summative assessment of

						examined the construct validity of this instrument.	crossed design and two facets (raters and occasions). In addition, we measured criterion validity by testing correlations with students' scores using other assessment methods.	rater interaction represented 17.7%, and the remaining 27.7% of the variance was due to unexplained sources of error. The decision (D) study suggested that an acceptable dependability (Φ = 0.70 and 0.72) can be achieved by using two raters for one and two occasions, respectively. Finally, we found moderate to large effect-size correlations between students' scores in reflective writing-based portfolios and communication skills (r = 0.47) and PBL tutorials (r = 0.50).	students' reflective writing-based portfolios in other medical schools.
96	Kassab, S. E. Bidmos, M. Nomikos, M. Daher-Nashif, S. Kane, T. Sarangi, S. Abu-Hijleh, M.	2020	Medical Students' Perspectives on an Assessment of Reflective Portfolios [Response to Letter]	-	-				

Supplementary File 2. Tabulated Summaries of PubMed's 50 relevant articles

S/N	Authors/Year	Article Title	Type of Study	Methodology	Themes	Key Findings	Proposed solutions/ conclusions
1	Amsellem-Ouazana, D. Van Pee, D. Godin, V.	Use of portfolios as a learning and assessment tool in a surgical practical session of urology during undergraduate medical training	Qualitative	For four consecutive terms of hospital training, four series of six students (5th and 6th year of medical studies) were briefed during their first hospital session that the evaluation of this particular session would be a portfolio. The setting of the portfolio in the ward took place as follows. First, after 15 days of training, each student had to choose a urological topic for his or her portfolio which was a problem to the student or merely one that he wanted to develop. The choice was made after observing an in or out patient. The students then had to write down what their objectives were in terms of knowledge to be acquired or to be developed. Each month, the student met a referring tutor (DAO); to follow the progress of the portfolio and redefine learning needs. The written report of this 'reorientation' meeting of research had to appear in the portfolio along with an assessment of the student's learning processes, particularly focused on the difficulties met and on the potential solutions. The students had been informed first of the kind of elements which could be placed in the folio: paper documents (books, course materials), documents from the	**Portfolio as a tool to Supplement learning through reflection**<br>Improve theoretical knowledge<br><br>**Portfolio as an assessment tool Used for summative assessment**<br>issues with summative assessment: The inter-examiner reliability is weak even if some authors believe that a more rigid structuring of the portfolio and of its evaluation grid would increase it (Davis et al., 2001). Moreover, students can choose not to mention the difficulties they may have encountered compiling the portfolio which cuts out important data on their apprenticeship.<br><br>**three crucial components for the effectiveness of portfolios:**<br>structure, mentor coaching and assessment<br><br>**Used with mentoring**<br><br>**student's positive perceptions of portfolio**	portfolio is a useful tool for self and reflective learning, mimicking authentic professional situations and allows a global reflection on medical reasoning. students look back on their actions systematically, analyse them with the aid of theoretical knowledge, resource persons, bibliographic researches, etc. and designate alternative methods of action.<br><br>We also used the portfolio as a summative assessment tool with a certification value allowing the student to move on to the next training session.<br><br>*Coaching:* The mentor helps the student identify his weaknesses and suggests a calendar in order to plan researches all through the term. This enables multiple interactions between the student and his tutor<br><br>students who were at first reluctant, greatly appreciated the making of a portfolio and widely acknowledged its pedagogical values.	We were surprised by the difficulties met by the students in writing out reports of the interviews (resource persons, tutor) or simply laying out on paper the questions they asked themselves and which motivated their researches. Nevertheless, we plan to widen the portfolio, as requested by the students, to encompass the technical aspects of surgical procedures; this will require identification of learning needs in this field at this stage of medical studies. Finally, in line with previous reports in the literature (Rees & Sheard, 2004), the students found the portfolio experience very satisfying and judged it to be have been an extremely useful experience. Their eagerness to repeat the portfolio experience has motivated us to continue with further developments in this field.

				internet (medical articles, information from patient or medical websites), and reports of interviews with resource persons. Summative assessment of the training session, for each student, was performed after the oral presentation of the portfolio (in front of the tutor for the first six students and in front of the department's medical staff for the remaining 18).			
2	Austin, C. Braidman, I. (2008)	Support for portfolio in the initial years of the undergraduate medical school curriculum: what do the tutors think?	Mixed methods	A mixed method approach was used with data collected from both questionnaires and a focus group meeting.	**Used with mentoring**<br><br>**Positive tutor's perceptions of portfolio use**<br>Felt equipped to foster reflective thinking with portfolios and support students<br><br>**Encouraged reflective learning**	Tutor facilitators were generally positive about their roles in the support of portfolio development in group sessions<br><br>identified several advantages: value of group discussion and discussion between peers to encourage reflection and the practical ability to integrate portfolio sessions more closely with clinical experiences - which would not be possible in one to one tutor/student meetings<br><br>With the training and guidance notes provided tutors were confident that they understood the support they should give students and felt that they could encourage reflection.	tutors perceived that the support of portfolio in groups is an effective way of developing portfolios for large numbers of students.
3	Avila, J. Sostmann, K. Breckwoldt, J. Peters, H. (2016)	Evaluation of the free, open source software WordPress as electronic portfolio system in undergraduate	Qualitative	We created an online ePortfolio environment using the blogging software WordPress based on reported capability features of such software by a qualitative weight and sum rfggyhujhhyh]. Technical implementation and usability were evaluated by 25 medical students during their	**e-portfolio use**<br><br>**mobile capabilities**<br>accessibility<br>wide spectrum of functions to document activities<br><br>**student's positive perceptions of e-portfolio**	**e-portfolio use**<br>Allowed students a broad spectrum of activities<br><br>**mobile capabilities**<br>– often documented via mobile devices – like collection of multimedia evidences, posting reflections, messaging, web	It is possible to build an advanced ePortfolio system with mobile capabilities with the free and open source software WordPress. This allows institutions without proprietary software to build a sophisticated ePortfolio system adapted to their needs with relatively few resources. The

		medical education		clinical training by quantitative and qualitative means using online questionnaires and focus groups.	content management system encouraged reflections information source for assessment<br>**exchanges with mentor**<br><br>**technical issues with e-portfolios**<br><br>**not as intuitive**	publishing, ePortfolio searches, collaborative learning, knowledge management in a content management system including a wiki and RSS feeds, and the use of aid tools for studying.<br><br>**technical issues with e-portfolios**<br>few technical problems<br><br>**student's positive perceptions of e-portfolio**<br>WordPress ePortfolio was rated positively by the students as a content management system (67 % of the students), for exchange with other students (74 %), as a note pad for reflections (53 %) and for its potential as an information source for assessment (48 %) and exchange with a mentor (68 %).<br><br>**not as intuitive**<br>74 % of the students in this pilot study did not find it easy to get started with the system, and 63 % rated the ePortfolio as not being user-friendly	implementation of WordPress should be accompanied by introductory courses in the use of the software and its apps in order to facilitate its usability.
4	Bashook, P. G.<br>Gelula, M. H.<br>Joshi, M.<br>Sandlow, L. J.<br>(2008)	Impact of student reflective e-portfolio on medical student advisors	Qualitative	Data were collected on Blackboard survey module for 8 volunteer advisors at two medical school campuses. Responses were hand coded, verified by two authors, tallied, with example comments recorded.	**Students' thought process when learning/ using portfolio**<br><br>**e-portfolio use**<br><br>**Enhanced interactions with students**<br><br>**Technical difficulties**	**Students' thought process when learning/ using portfolio**<br>the students' responses in the portfolio gave advisors greater insight into students' thinking, maturity, and reflective ability, and they helped advisors identify early warnings about problems.	Advisors reported students' reflective responses to focused questions in an e-portfolio contribute valuable understanding about students' thinking and attitudes. Advisors are enthusiastic about the value of the e-portfolio for this purpose. We anticipate benefits will generalize when fully implemented.

						**Enhanced interactions with students**<br>The e-portfolio enhanced meaningful interactions and more focused discussions with students. Advisors reported no improvements in efficiency of communications<br><br>**Technical difficulties**<br>technical difficulties with Blackboard (version 6.0).	
5	Belcher, R.<br>Jones, A.<br>Smith, L. J.<br>Vincent, T.<br>Naidu, S. B.<br>Montgomery, J.<br>Haq, I.<br>Gill, D. (2014)	Qualitative study of the impact of an authentic electronic portfolio in undergraduate medical education	Qualitative	a questionnaire survey with extensive free text comments was used at School 1, and three focus groups were held at School 2. This paper reports thematic analysis of students' opinions expressed in the free text comments and focus groups.	**Purpose, use and acceptability**<br><br>**Advantages of using the UMeP**<br><br>**Barriers to the use of the UMeP**<br>• lack of faculty engagement<br>• software issues (e-portfolio)<br>• security of their data<br>• accessibility<br>• tick box culture<br><br>**Impact on learning**<br>Depends on quality of feedback<br>• based on timing - delay between any observation and subsequent completion of the UMeP form,<br>• quality of feedback<br>• lack of engagement of their supervisors with the feedback process.<br><br>**Professional identity**<br><br>**Fostered positive effects on self-perception**	students' views varied on the role and purpose of the UMeP, and this was also coloured by how the School was using the UMeP and the School's stance with regards to the requirement to engage.<br><br>**Advantages of using the UMeP**<br>Students identified a number of advantages to the use of portfolios in general and specifically electronic portfolios. They noted that assessment drives learning and in School 2 where work-based assessments were integral, the tool particularly encouraged workplace-based learning.<br><br>**Barriers to the use of the UMeP**<br>• lack of faculty engagement<br>• software issues (e-portfolio)<br>• security of their data<br>• accessibility - in hospital settings, it was difficult for students to find a computer to complete the assessment immediately, requiring them to	whilst there are barriers to its use, students also recognise the potential advantages, and the positive impact on professional enculturation. Students constructed their opinions of the ePortfolio from multiple sources, and were influenced significantly by the supervisors and junior doctors they encountered, as well as their own experiences.<br><br>Providing our students with **early exposure** to the portfolio tool that they are likely to use as Foundation doctors will, we anticipate, support them in their transition from medical student to doctor. Our first cohort of students, who have used the ePortfolio throughout their clinical training have now graduated, and we plan further collaborative research to follow them in to the Foundation programme, and investigate the effects of using an authentic portfolio as a student.

					**Not understanding the need of maintaining portfolio**	send an e-ticket to the assessor to be completed later. disconnect between performance of the assessment and completion of written feedback contributed to poor feedback, as some supervisors waited a considerable time before completing the online feedback, when they then struggled to remember individual students.<br>• tick box culture *We will just do it to get it done and meet deadlines, rather than for its actual purpose*<br><br>**Impact on learning**<br>Students felt quality of feedback they received meant they did not see an impact on learning. k of usefulness of the feedback was due to the delay between any observation and subsequent completion of the UMeP form, the rigidity of the forms used and a lack of engagement of their supervisors with the feedback process. Since feedback was poor-quality or non-existent, students confessed that they did not always read their feedback, and that this was a further contributing factor to the perception of the UMeP as a "tick box exercise"<br><br>**Professional Identity**<br>Students at both schools indicated that they thought it likely that using the ePortfolio	

						as students would assist them as Foundation doctors.<br>Students at School 2 were aware of, and commented extensively on the use of portfolios by doctors, and felt that participating in portfolio keeping contributed to their own enculturation into the profession.<br>Their comments suggested their shared frustrations with the ePortfolio helped forge relationships with senior colleagues.<br><br>**positive effects on self-perception**<br>authenticity of the portfolio also had positive effects on students' perception of themselves as becoming part of the profession<br><br>**Not understanding the need of maintaining portfolio**<br>In common with junior doctors [13],[14], students in our study sometimes struggled to understand the purpose of maintaining a portfolio.	
6	Buckley, S.<br>Coleman, J.<br>Davison, I.<br>Khan, K. S.<br>Zamora, J.<br>Malick, S.<br>Morley, D.<br>Pollard, D.<br>Ashcroft, T.<br>Popovic, C.<br>Sayers, J.<br>(2009)	The educational effects of portfolios on undergraduate student learning: a Best Evidence Medical Education (BEME) systematic	Mixed methods	We developed a protocol based on the recommendations of the Best Evidence Medical Education (BEME) collaboration. Citations retrieved by electronic searches of 10 databases were assessed against pre-defined inclusion/exclusion criteria by two independent reviewers and full texts of potentially relevant articles were obtained. Studies were identified for inclusion in the review by examination of full text	**e-portfolio use**<br><br>**data collection methods**<br>• combination of data collection methods<br>• questionnaires<br>• focus group interviews<br>• direct assessment of portfolios<br><br>**Aim of study**<br>Mos studies assessed student or tutor perceptions of the	Nineteen of the 69 included studies (27%) met seven or more quality indicators. Across all professions, such 'higher quality' studies were more likely to have been published recently. The median 'quality score' (number of indicators met) rose from two for studies published in 2000 or earlier to seven for studies published in 2005 or later.	whilst portfolios encourage students to engage in reflection, the quality of those reflections cannot be assumed and that the time commitment required for portfolio completion may detract from other learning or deter students from engaging with the process unless required to do so by the demands of assessment. Further work is needed to strengthen the evidence base for portfolio use, particularly comparative studies which observe

		review. BEME Guide No. 11		articles by two independent reviewers. At all stages, discrepancies were resolved by consensus. Data relating to characteristics of the student population, intervention, outcome measures, study design and outcomes were collected using a piloted data extraction form. Each study was assessed against 11 quality indicators designed to provide information about how well it was designed and conducted; and against the Kirkpatrick hierarchy as modified for educational settings. Comparisons between different groups were carried out using the Kruskal–Wallis test (non-parametric ANOVA) or the Mann–Whitney U test as appropriate.	effect of the use of portfolios on their learning.<br><br>**Design**<br>comparative design<br>randomized controlled trial<br><br>**main effects of portfolio use**<br>• **improvement in student knowledge and understanding,** particularly the ability **to integrate theory with practice**<br>• greater **self-awareness** and encouragement of r**eflection**<br>• ability to **learn independently**<br>• **Improves feedback** to students and **gives tutors a greater awareness of students' needs,** may help students to cope with **uncertain or emotionally demanding situations**<br>• **prepares students for postgraduate settings** in which reflective practice is required<br><br>**Used with mentoring**<br><br>**Quality of reflection/feedback**<br>some studies questioned the *quality* of the reflection undertaken.	The main effects of portfolio use identified by the included studies were: Improvement in student knowledge and understanding (28 studies, six at Kirkpatrick level 2 or above), greater self-awareness and encouragement of reflection (44 studies, seven at Kirkpatrick level 2 or above) and the ability to learn independently (10 studies, one at Kirkpatrick level 2). The findings of higher quality studies also identified benefits in these areas.	changes in student knowledge and abilities directly, rather than reporting on their perceptions once a portfolio has been completed.
7	Buckley, S.<br>Coleman, J.<br>Khan, K. (2010)	Best evidence on the educational effects of undergraduate portfolios	Qualitative	Using a methodology based on BEME recommendations, we searched the literature relating to a range of health professions, identifying evidence for the effects of portfolios on undergraduate student learning,	**Improve student knowledge and understanding**<br><br>**greater self-awareness and encouragement of reflection**	**Improve student knowledge and understanding**<br>can improve students' ability to integrate theory with practice<br><br>**greater self-awareness and encouragement of reflection**	An analysis of methodological quality against year of publication suggests that, across a range of health professions, the quality of the literature relating to the educational effects of portfolios is improving. However, further work is still

				and assessing the methodological quality of each study.	**help students to cope with uncertain or emotionally demanding situations**<br><br>**used with mentoring & improve relationships**<br><br>**prep students for postgraduate training**<br><br>**barriers to portfolio use**<br>time consuming	encourage their self-awareness and reflection,<br><br>**help students to cope with uncertain or emotionally demanding situations**<br>offer support for students facing difficult emotional situations.<br><br>**prep students for postgraduate training**<br>enhance student–tutor relationships and prepare students for the rigours of postgraduate training<br><br>**barriers to portfolio use**<br>time required to complete a portfolio may detract from students' clinical learning.	required to build the evidence base for the educational effects of portfolios, particularly comparative studies that assess effects on learning directly.
8	Byszewski, A.<br>Fraser, A.<br>Lochnan, H.<br>(2018)	East meets West: Shadow coaching to support online reflective practice	Qualitative	A structured, reflection-based electronic portfolio program (ePortfolio), with novel faculty development initiative, involving 'shadow coaches', was shared with the newly formed Ottawa-Shanghai Joint School of Medicine (OSJSM). OSJSM is a partnership between Shanghai Jiao Tong University and the University of Ottawa. As the world's first Sino-Canadian Joint Medical School, OSJSM introduced North American undergraduate medical curriculum to China. 'Shadow coaching' involved trans-Pacific pairing of coaches, supplemented by local faculty development.<br><br>(a) Pre-implementation: The well-established online ePortfolio	**Used with mentoring/ coaching**<br><br>**Effective in developing coaches skills**<br><br>**Variability in Quality of reflection**	Initial results indicate that there is some variability in the quality of student reflection and coach support. Overall, the shadow coaches reported that the posts of the OSJSM students were meeting the ePortfolio requirements and that most of the reflections were of good quality. Shadow coaches agreed that posting and reflecting on clinical experiences may be extra challenging for students during the preclinical phase. The students, like their Canadian counterparts, experienced pressure and competition in the first year of their study that made it difficult to prioritize ePortfolio. The feedback provided by the OSJSM coaches was for the most part	Shadow coaching should be considered an important faculty development tool that is effective across large geographic distances. A strong and situationally appropriate faculty development program is vital to the success of portfolio programs and is strongly enhanced by real-time interactions online. Shadow coaching is an example of a novel peer-teaching initiative to provide distance faculty development in a cross-cultural collaboration. As more academic institutions move toward internationalization, shadow coaching can promote and support best practices.

				platform at the University of Ottawa was mirrored at OSJSM. University of Ottawa ePortfolio coaches were recruited to serve as shadow coaches to their OSJSM counterparts. Shadow coaches provided mentoring and resources while maintaining awareness of cross-cultural issues. Faculty development consisted of face-to-face faculty development in Shanghai, several online synchronous sessions, and familiarization of University of Ottawa coaches with the Chinese medical education system.<br>(b) Description/Components: This intervention, introduced in 2016–2017, involved five University of Ottawa shadow coaches paired with five OSJSM ePortfolio coaches. Student reflection encourages open frank discussion which is a new paradigm for Chinese students and faculty. Shadow coaches were encouraged to challenge new OSJSM coaches to widely explore physician roles and competencies.		found to be supportive and affirmative; coaches frequently reassured students by sharing similar experiences of their own. Conversely, OSJSM coaches rarely probed or challenged the students to explore concepts more deeply.	
9	Byszewski, A.<br>Gill, J. S.<br>Lochnan, H.<br>(2015)	Socialization to professionalism in medical schools: a Canadian experience	Qualitative	A literature review was performed and with the input of the AFMC(Association of Faculties of Medicine of Canada) Professionalism group, questionnaires were generated. An electronic survey was circulated to key leaders across the country at all the medical schools. In-depth telephone interviews were used to further explore themes, and a	**Portfolio as an assessment tool for professionalism competency**<br><br>**Supports students in later years of med sch: struggles with complex issues in clinical context**<br><br>**Portfolios as a teaching tool**<br><br>**use**	**Portfolio as an assessment tool for professionalism competency**<br>Portfolios, consisting of reflective exercises documenting development of professionalism competency, are now used by 42.9 and 30.8 % respectively<br><br>The CanMEDS 2005 framework in operation at the time was not	Although with this project we have seen some further development of innovative methods over the 5 years as compared to a previous study (eportfolios, reflective writing exercises, multisource feedback, etc.), there remains room to improve on the delivery of the curriculum, particularly in clerkship and around reporting of lapses and remediation. If we are to support the professional identity formation of the physicians

				subsequent focus group was held to discuss challenges, particularly related to reporting and remediation.		entirely explicit in recommending specific strategies for program delivery but it provides some assessment tool suggestions for the professional role, including direct observation, multisource feedback, and portfolios. Our results indicate that most schools use direct observation, but just over 40 % use portfolios and 23 % use multisource feedback<br><br>**Supports students in later years of med sch: struggles with complex issues in clinical context**<br>Professional identity formation occurs throughout the course of medical school, with a major shift in third year or early clerkship. Part of this process in later years of medical school involves struggling with complex issues encountered in a clinical context and ensuring there is a structure to support the learners in that process. Participants commented that portfolios or debriefing sessions can assist in this measure<br><br>**Portfolios as a teaching tool**<br>teaching methods<br><br>**e-portfolio use**	of tomorrow, we owe it to our learners to meet this challenge.
10	Chertoff, J.<br>Wright, A.<br>Novak, M.<br>Fantone, J.<br>Fleming, A.<br>Ahmed, T.	Status of portfolios in undergraduate medical education in the LCME	Quantitative	A 21-question survey regarding portfolios was sent to the 141 LCME-accredited, US medical schools. The response rate was 50% (71/141); 47% of respondents (33/71) reported	**e-Portfolio use**<br>**paper portfolio use**<br><br>**used for Assessment**<br>• formative evaluation<br>• summative evaluation	Seventy-two percent developed a longitudinal, competency-based portfolio. The most common feature of portfolios was reflective writing (79%). Seventy-three percent allow	While there is significant variation in the purpose and structure of portfolios in the medical schools surveyed, most schools using portfolios reported a high level of

	Green, M. M.<br>Kalet, A.<br>Linsenmeyer, M.<br>Jacobs, J.<br>Dokter, C.<br>Zaidi, Z. (2016)	accredited US medical school		that their medical school used portfolios in some form. Of those, 7% reported the use of paper-based portfolios and 76% use electronic portfolios. Forty-five percent reported portfolio use for formative evaluation only; 48% for both formative and summative evaluation, and 3% for summative evaluation alone.	• both formative and summative evaluation<br><br>**Features of Portfolios**<br>• reflective writing<br>• allowed access to portfolio off campus<br>• involved social media within portfolio<br><br>**+ve Effects of portfolio**<br>• Engaged students and faculty<br><br>**Portfolios need to be improved**	access to the portfolio off-campus, 58% allow usage of tablets and mobile devices, and 9% involve social media within the portfolio. Eighty percent and 69% agreed that the portfolio engaged students and faculty, respectively. Ninety-seven percent reported that the portfolios used at their institution have room for improvement.	engagement with students and faculty.
11	Davis, M. H.<br>Ponnamperuma, G. G. (2010)	Examiner perceptions of a portfolio assessment process	Quantitative	A questionnaire containing statements and open questions was used to obtain examiner feedback. Responses to each statement were compared over 3 years: 1999, 2000 and 2003.	**Assessment Tool**<br>**• More time for assessment**<br><br>**Portfolio content**<br>**• too many content**<br><br>**Holistic snapshot of candidate as Strengths of assessment process**<br><br>**Examiner Training for portfolio assessment**	**Portfolio content**<br>Too much portfolio material is a well-documented problem that seems hard to overcome (Athenases 1997). To address this issue, material that provides no new information about the student and insufficiently reliable material should be omitted from the portfolio.<br><br>**Holistic snapshot of candidate as Strengths of assessment prcoess**<br>comprehensive, holistic picture of the candidate provided by the portfolio assessment process was perceived to be one of its strengths<br><br>**More time for assessment**<br>The examiners feel rushed in their contribution to the portfolio assessment process. Although there was a significant change in this opinion with substantial effect sizes in the last 2 years,	The 12 exit learning outcomes of Dundee curriculum provide an appropriate framework for the portfolio assessment process, but the content of the portfolio requires fine-tuning particularly with regard to quantity. Time allocated to examiners for the portfolio assessment process needs to be balanced against practicability. The holistic picture of the candidate provided by the process was one of its strengths.

						as opposed to the first year, the examiners were still uncertain as to the adequacy of the time allocated to them for their part of the portfolio assessment process.	
12	Davis, M. H.<br>Ponnamperuma, G. G.<br>Ker, J. S. (2009)	Student perceptions of a portfolio assessment process	Quantitative	A questionnaire containing statements and open questions was used to obtain feedback from students at the University of Dundee Medical School, Scotland. The responses to each statement were compared over 4 years (1999, 2000, 2002 and 2003).	**Perceptions of portfolio use by students**<br><br>**Enhanced learning**<br>**• understanding of topics**<br>**• reflection**<br><br>**-ve attitude towards portfolio initially**<br><br>**Shifted to +ve perception**<br><br>**Assessment tool**<br><br>**High commitment**	**Enhanced learning**<br>A major finding is that students perceived that portfolio building heightened their understanding of the exit learning outcomes and enabled reflection on their work<br><br>**-ve attitude towards portfolio initially**<br>Student reactions to the portfolio process were initially negative, although they appreciated that senior staff took time to become familiar with their work through reviewing their portfolios.	Paperwork should be kept within manageable limits. A student induction process that highlights the importance of providing evidence for achieving all learning outcomes, not just theoretical knowledge and skills, may be helpful in allaying student concern over portfolio building and assessment and support preparation for lifelong learning and reflective clinical practice.

						**Shifted to +ve perception**<br>Student attitudes became more positive over the 4 years as the process evolved.<br><br>**High commitment**<br>Although portfolio assessment was recognised as supporting student learning, portfolio building was perceived to interfere with clinical learning as a result of the excessive amounts of paper evidence required	
13	Dornan, T.<br>Lee, C.<br>Stopford, A.<br>Hosie, L.<br>Maredia, N.<br>Rector, A.<br>(2005)	Rapid application design of an electronic clinical skills portfolio for undergraduate medical students	Descriptive	A clinician-educator, two medical students, two computing science masters students, two other project workers, and a hospital education informatics lead, formed a design team. A sample of stakeholders took part in requirements planning workshops and continued to advise the team throughout the project. A university hospital had many features that favoured fast, inexpensive, and successful system development: a clearly defined and readily accessible user group; location of the development process close to end-users; fast, informal communication; leadership by highly motivated and senior end-users; devolved authority and lack of any rigidly imposed management structure; cooperation of clinicians because the project drew on their clinical expertise to achieve scholastic goals; a culture of learning and involvement of highly motivated students. A detailed specification	**e-portfolio use**<br><br>**reflective portfolio to supplement learning**<br><br>**used as logbook**	**reflective portfolio to supplement learning**<br>help students reflect on their objectives and progress towards achieving them. Each time the user practices a skill, they can enter a self, peer or tutor assessment. To do that, they rate their competence at the skill against a 'competence ladder', worded specifically for that skill; they can also enter a free text reflection on their acquisition of the skill.<br><br>**used as logbook**<br>document their progress in attaining skills, as judged by self, peer and tutor assessment, and examinations	We believe the success of RAD in our medical school was more than coincidental. It is best suited to small, user-led projects like this one, which do not need major infrastructure development. Collaboration between staff and students is a necessary condition for learner-centred education, and the positive climate in our own medical school that has resulted from curriculum change has attracted favourable comment in external peer review. Staff and students are accustomed not just to collaborative working, but doing so in groups. The analytical behaviour of an RAD group has much in common with a PBL tutorial group. We believe the success of RAD in our school may be generalisable to other schools, and increasingly important as medical education worldwide moves both towards more collaborative modes of education and towards e-learning.

				was developed through storyboarding, use case diagramming, and evolutionary prototyping. A very usable working product was developed within weeks. "SkillsBase" is a database web application using Microsoft Active Server Pages, served from a Microsoft Windows 2000 Server operating system running Internet Information Server 5.0. Graphing functionality is provided by the KavaChart applet. It presents the skills curriculum, provides a password-protected portfolio function, and offers training materials.			
14	Driessen, E.<br>van Tartwijk, J.<br>Vermunt, J. D.<br>van der Vleuten, C. P. (2003)	Use of portfolios in early undergraduate medical training	Descriptive	This article describes the use of portfolios in early undergraduate medical training.<br><br>Two hundred and forty-two first-year students compiled a portfolio during the academic year 2001–02. Semi-structured interviews were held with a select group of students to explore the effect of the portfolio on reflection. Students from four random mentor groups, 39 in total, were approached and asked if they would be interviewed on the portfolio	**Effectiveness of portfolios rely on**<br>**• structure**<br>**• coaching/mentoring**<br>**• assessment**<br><br>**Variations in Portfolio Structure**<br><br>**Considerations in structuring portfolios**<br><br>**Used with mentoring**<br><br>**Reliability of assessing portfolios**<br><br>**Stimulating reflection in portfolios is a Protective factor to clerkship years**	**Structuring**<br>An overly structured portfolio will readily deteriorate into a tick-off list through which students can demonstrate that they have met the expectations. This results in students showing forced study behaviour and being mainly occupied with getting the learning results needed ticked off<br>however, little or no structure is provided for the portfolio, students often have no idea how to go about it<br>The structure of the portfolio is as follows: ● curriculum vitae; ● role as medical expert; ● role as researcher; ● role as healthcare worker; ● role as person; ● general: – summary of strengths and weaknesses analysis; – report of progress interview and/or of exit interview/ advice ● annexes.	main reason for the introduction of portfolios in early undergraduate medical training was to develop the reflective ability of early undergraduate students. Reflective ability is seen as an important skill to learn from practice and for lifelong learning.The ability to reflect can protect students from the shock of practice when entering their clerkships.<br><br>In conclusion, we feel that a portfolio constructed according to the considerations described in this paper is a worthwhile addition to existing assessment and learning tools

						**mentoring**<br>A crucial factor for the effective use of portfolios aimed at stimulating reflection is regular discussion of the portfolio with others. The mentor's responsibility goes beyond supervising the portfolio: he/she also has a general pastoral function. If a student has a problem or a question that has consequences for his/her studies, he/she may direct him/herself to his/her mentor. If he/she has problems of a structural nature, the mentor can refer the student to a student counsellor or other, specialized professionals.<br><br>**Assessment**<br>The assessment literature has shown that assessment drives learning and that congruence between training and assessment is imperative (Driessen & van der Vleuten, 2000). Assessment can also be used to steer student learning in a desirable direction.<br><br>**Reliability of assessing portfolios**<br>Reliability, perceived of as inter-rater reliability, may be improved by three strategies: portfolio standardization; assessment objectification by using analytical criteria; and increasing the number of assessors	
15	Driessen, E. W. van Tartwijk, J.	Conditions for successful	Qualitative	We designed a portfolio that was **aimed at stimulating reflection**	**Stimulating reflection**	**conditions for successful reflective use of portfolios**	This study shows that portfolios are a potentially valuable method of

	Overeem, K. Vermunt, J. D. van der Vleuten, C. P. (2005)	reflective use of portfolios in undergraduate medical education		**in early undergraduate medical education**, using experiences described in the medical education literature and elsewhere. Conditions for reflective portfolio use were identified through interviews with 13 teachers (mentors), who were experienced in mentoring students in the process of developing their portfolios. The interviews were analysed according to the principles of grounded theory.	**conditions for successful reflective use of portfolios**<br>• coaching<br>• portfolio structure<br>• experiences and materials<br>• summative assessment<br><br>**Assessment tool in assessing reflection skills**<br>Enhances assessment<br><br>**Teaching tool in developing reflective skills**<br>Enhances learning	coaching; portfolio structure and guidelines; relevant experiences and materials, and summative assessment. According to the mentors, working with a portfolio designed to meet these conditions will stimulate students' reflective abilities.	assessing *and* developing students' reflective skills in undergraduate medical training, provided certain conditions for effective portfolios are recognised and met. Portfolios have a strong potential for enhancing learning and assessment but they are very vulnerable and may easily lead to disappointment. Before implementing portfolios in education, one should first consider whether the necessary conditions can be fulfilled, including an appropriate portfolio structure, an appropriate assessment procedure, the provision of enough new experiences and materials, and sufficient teacher capacity for adequate coaching and assessment.
16	Driessen, E. W. Muijtjens, A. M. van Tartwijk, J. van der Vleuten, C. P. (2007)	Web- or paper-based portfolios: is there a difference?	Qualitative	An experimental design was used to compare Year 1 medical students' reflective portfolios. The portfolios differed in presentation medium only (i.e. web-based versus paper-based). Content analysis, a student questionnaire and mentor interviews were used to evaluate portfolio quality, user-friendliness and student motivation. A total of 92 portfolios were scored independently by 2 raters using a portfolio quality-rating instrument.	**Paper VS e-portfolios**<br><br>**Perceptions of portfolio use**<br><br>**Portfolio structure**<br><br>**Reflective component**<br><br>**Used with mentoring**<br><br>**Students more motivated when using e-portfolios**<br><br>**No difference in Quality**<br><br>**e-portfolios are more time-consuming**<br><br>**No difference in satisfaction**<br><br>**Mentors perception of e-portfolios**	**Portfolio structure**<br>Portfolio structure, quality of reflection and quality of evidence showed no significant effects of presentation medium.<br><br>**Students more motivated when using e-portfolios**<br>Multi-level analysis showed a significant effect for student motivation: web-based portfolios scored 0.39 more than paper-based portfolios ($P < 0.05$; effect size 0.76).<br><br>**No difference in Quality**<br>the mentors reported no differences in portfolio quality, except that there were more visuals in web-based portfolios<br><br>**e-portfolios are more time-consuming**	The web-based portfolios were found to enhance students' motivation, were more user-friendly for mentors, and delivered the same content quality compared with paper-based portfolios. This suggests that web-based presentation may promote acceptance of portfolios by students and teachers alike.

						Students spent significantly more time preparing the web-based than the paper-based portfolios (15.4 hours versus 12.2 hours; t = 2.1, P < 0.05; effect size 0.46).<br><br>**No difference in satisfaction**<br>The 2 student groups did not differ significantly in terms of their satisfaction with the portfolio.<br><br>**Mentors perception of e-portfolios**<br>The mentors perceived the web-based portfolios as more user-friendly.	
17	Driessen, E. W.<br>Overeem, K.<br>van Tartwijk, J.<br>van der Vleuten, C. P.<br>Muijtjens, A. M.<br>(2006)	Validity of portfolio assessment: which qualities determine ratings?	Qualitative	We examined the possible effects of such qualities in a portfolio programme aimed at stimulating Year 1 medical students to reflect on their professional and personal development. In later curricular years, this portfolio is also used to judge clinical competence.	**Assessment tool**<br><br>**Teaching tool**<br><br>**Forms of portfolio**<br><br>**Content of portfolio**<br><br>**Reflective component**<br>**• quality of reflection**<br><br>**Validity of reflection**	**Reflective component**<br>criteria for reflective competence used in the regular portfolio assessment procedure<br><br>**Validity of reflection**<br>the strongest predictor of the variance in the regular ratings was 'quality of reflection' (R 0.80; R2 66%). No further items accounted for a significant proportion of variance. Irrelevant items, such as writing style and lay-out, had negligible effects. The absence of an impact of irrelevant criteria appears to support the validity of the portfolio assessment procedure.	Further studies should examine the portfolio's validity for the assessment of clinical competence.
18	Elango, S.<br>Jutti, R. C.<br>Lee, L. K.<br>(2005)	Portfolio as a learning tool: students' perspective	Quantitative	A questionnaire survey was conducted among 143 medical students to find out their perceptions of the portfolio as a learning tool	**Perceptions of portfolio use**<br><br>**Learning, teaching and assessment tool**<br><br>**Good learning tool**	**Good learning tool**<br>A majority of the students felt that the portfolio is a good learning tool.	The study indicates that students need appropriate guidance from the academic staff for the system to succeed.

					**Stressful and time-consuming**	**Stressful and time-consuming**<br>stressful and time-consuming to develop a proper portfolio.	
19	Fida, N. M.<br>Hassanien, M.<br>Shamim, M. S.<br>Alafari, R.<br>Zaini, R.<br>Mufti, S.<br>Al-Hayani, A.<br>Farouq, M.<br>Al-Zahrani, H.<br>(2018)	Students' perception of portfolio as a learning tool at King Abdulaziz University Medical School	Mixed method	Portfolios were introduced in the 2nd through 5th years at King Abdulaziz University over a two-year period (2013–2015). At the end of each academic year, students completed a mixed questionnaire that included a self-assessment of skills learned through the use of portfolio.	**Different focus in portfolios: preclinical VS clinical years**<br><br>**ve perceptions of portfolios**<br><br>**used with mentoring**<br>• interactions with mentor, getting feedback<br>• portfolio use could be dependent on mentor<br>• **i**ncreases in the frequency and quality of feedback involving even a single procedure contributed to improving medical students’ “global procedural performance”<br><br>**Learning tool**<br>• reflective components<br><br>**assessment tool**<br>• formative assessment	**Different focus in portfolios: preclinical VS clinical years**<br>The results showed a difference in focus between basic and clinical years: in basic years students’ focus was on acquiring practical skills, but in clinical years they focused more on acquiring complex skills, including identifying and managing problems.<br><br>**+ve perceptions of portfolios**<br>revealed a positive trend in acceptance (belief in the educational value) of portfolios among students and their mentors, across the years of the program.	Those who intend to implement portfolios, in our view, should consider carefully whether they will be able to create the favorable learning environment that is needed for successful portfolio use. As also noted, this includes consideration not only of factors associated with the educational culture of the institution, but also of factors pertaining to the broader socio-cultural context. Only when such considerations have been taken into account, we believe, can medical schools move forward to effectively implement – or broaden their implementation of – portfolio-based learning.
20	Figueroa, C.<br>Calvo, I.<br>González, C.<br>Sandoval, D.<br>Padilla, O.<br>Le Roy, C.<br>Delfino, A.<br>Arab, J. P.<br>Pizarro, M.<br>Solís, N.<br>Riquelme, A.<br>(2015)	[Introduction of virtual patients to clinical case portfolios for undergraduate medical students]	Qualitative	Virtual patients were implemented based on prototype clinical cases with specific syndromes. Students□ perceptions about CCP before and after the introduction of virtual patients were evaluated using a validated questionnaire that was answered voluntarily and anonymously.	**Teaching Tool**<br>**• learning**<br><br>**Assessment Tool**<br><br>**Improvement in Perception of portfolio use**	**Assessment Tool**<br>a Clinical-Case-Portfolio (CCP) was introduced as a new assessment instrument for fourth grade undergraduate medical students.<br><br>**Improvement in Perception of portfolio use**<br>perception of CCP significantly improved after the incorporation of virtual patients (97.1 ± 24.9 and 111.3 ± 25.7 points; 57.8 and 66.2% respectively)	The incorporation of virtual patients improved undergraduate students□ perception of CCP.

						**portfolio domains**<br>Student Learning□, □Organization and Evaluation□, □Teaching Methodology□ and □Integration	
21	Fishleder, A. J.<br>Henson, L. C.<br>Hull, A. L.<br>(2007)	Cleveland Clinic Lerner College of Medicine: an innovative approach to medical education and the training of physician investigators	Descriptive		**Assessment tool**<br>• competency assessment<br>• formative feedback<br>• self-assessment<br>• formative and summative assessment<br><br>**Teaching and reflective tool**<br>• enhancing learning - goal of fostering self-directed learning and reflective practice<br><br>**Used with coaching**	s. A unique portfolio-based assessment system is used to assess student achievements in nine competency areas, seven of which reflect the Accreditation Council for Graduate Medical Education competencies<br><br>**Teaching tool**<br>each student maintains an educational portfolio to document evidence of his or her individual progress in each of the nine areas of core competency.<br><br>**Used with coaching**<br>Each student has a physician adviser with whom he or she works in partnership for five years. Physician advisers meet regularly as a group to ensure a uniform approach to assessing student performance and advising student progress.	
22	Gaffan, J.<br>Dacre, J.<br>Jones, A. (2006)	Educating undergraduate medical students about oncology: a literature review	Qualitative	MEDLINE, Psychinfo, ERIC, TIMELIT, EMBASE, CINAHL and the Cochrane CENTRAL Register of Controlled Trials (CENTRAL) were searched, using the search terms cancer, oncology, education, undergraduate, and teaching.	**Assessment tool**<br><br>**Teaching tool**<br><br>**+ve perceptions of portfolio**<br><br>**Improved test performance**	**+ve perceptions of portfolio; Improved test performance**<br>The portfolio learning was popular—90% of students felt that it was a worthwhile and valuable experience. In the assessment, there was a trend for the intervention group to perform better in oncology questions	We have found 48 articles on undergraduate teaching in oncology. Oncology teachers should consider adopting the evidence based approaches outlined in this review, and there should be more emphasis on educational research within the field of oncology.

						**Teaching tool**<br>Teaching knowledge: portfolio learning is at least as successful as a standard oncology curriculum;	
23	Haffling, A. C.<br>Beckman, A.<br>Pahlmblad, A.<br>Edgren, G.<br>(2010)	Students' reflections in a portfolio pilot: highlighting professional issues	Mixed methods	Thirty-five voluntary final-year medical students piloted a standardized portfolio in a general practice (GP) attachment at Lund University, Sweden. Students' portfolio reflections were based upon documentary evidence from practice, and aimed to demonstrate students' learning. The reflections were qualitatively analysed, using a framework approach. Students' evaluations of the portfolio were subjected to quantitative and qualitative analysis.	**Reflective portfolios for learning and develop professionalism**<br>Self-reflected on their 'Process skills', 'Perceptual skills', 'Content skills' and 'Context' after examiner interview<br><br>**Assessment tool**<br>• formative assessment<br>• self-assessment<br><br>**+ve perceptions of portfolio**<br><br>**Areas of improvement for portfolios**<br><br>**Used with coaching/mentor**	**Reflective portfolios**<br>provided ample opportunities for reflections on professional issues<br><br>Students were satisfied with the portfolio, but improved instructions were needed.<br><br>**Used with coaching/mentor**<br>Support by mentors and a final examiner interview contributed to the success of the portfolio with students.	this study demonstrated that a structured portfolio in a short timeframe provided abundant opportunities for students to reflect on personal and professional issues, notwithstanding the standardized format. We strongly recommend a final examiner interview to deepen students' reflections, to supply feedback and to raise the importance of reflective ability as a skill in future learning from practice.
24	Hall, P.<br>Byszewski, A.<br>Sutherland, S.<br>Stodel, E. J.<br>(2012)	Developing a sustainable electronic portfolio (ePortfolio) program that fosters reflective practice and incorporates CanMEDS competencies into the undergraduate medical curriculum	Descriptive	the authors describe the development of an electronic Portfolio (ePortfolio) program that enables uOttawa medical students to document their activities and to demonstrate their development of competence in each of the eight roles.	**Supports reflective practice**<br>• Safe environment for reflective practice<br>• allow learners to actively engage in self-assessment through reflection and feedback<br><br>**e-portfolios**<br><br>**used with group discussions**<br><br>**improves learning outcomes**<br><br>**better interactions with tutor/mentor**	**Supports reflective practice**<br>supports reflective practice, an important component of professional competence, and provides a means for addressing the "hidden curriculum."<br><br>**Logbook**<br>purposeful collection of information and digital artifacts that demonstrates development or evidences learning outcomes, skills, or competencies<br><br>**Safe environment for reflective practice**	Portfolios have traditionally been used as a vehicle for charting professionalism. Our ePortfolio expands their application to a wider range of physician roles.

					**logbook**	Portfolio learning encourages students to debrief challenging events that occur during the course of their learning. It provides a safe environment in which they can reflect, receive feedback, and engage in discourse around context-specific experiences<br>**better interactions with tutor/mentor**<br>enable the student to have a discourse with himself or herself, these conversations with the self can grow into dialogues with others,22 particularly the teacher or mentor.	
25	Kalet, A. L.<br>Sanger, J.<br>Chase, J.<br>Keller, A.<br>Schwartz, M. D.<br>Fishman, M. L.<br>Garfall, A. L.<br>Kitay, A. (2007)	Promoting professionalism through an online professional development portfolio: successes, joys, and frustrations	Descriptive	The authors developed and implemented a program built around a Web-based Professional Development Portfolio (PDP) to assess and document professional development in medical students at New York University School of Medicine. This program requires students to regularly document their professional development through written reflections on curricular activities spanning preclinical and clinical years.	**e-portfolio use**<br><br>**Reflective component**<br><br>**Professional development**<br><br>**perceptions of students**<br><br>**used with mentoring**<br>**Learning & teaching tool**<br>Self-directed learning<br><br>**Assessment tool**	A Web-based PDP promoted self-regulation on an individual level because it facilitated narrative reflection, self-assessment, and goal setting, and it structured mentorship.	Therefore, the PDP may prepare students for the self-regulation of the medical profession--a privilege and obligation under the physician's social contract with society.
26	Lonka, K.<br>Slotte, V.<br>Halttunen, M.<br>Kurki, T.<br>Tiitinen, A.<br>Vaara, L.<br>Paavonen, J.<br>(2001)	Portfolios as a learning tool in obstetrics and gynaecology undergraduate training	Qualitative	The study population consisted of 91 medical students who completed the portfolio during their training course. The portfolio consisted of a 28-page A5-size booklet. The students entered all the clinical procedures they had performed and all the deliveries they had attended. After each group session, they answered	**Improvements in final exam**<br><br>**Logbook**<br><br>**Learning tool**<br><br>**+ve perceptions of portfolio**<br><br>**Support personal & professional development of students**	**Improvements in final exam**<br>The amount of text written in the portfolio correlated ($P < 0.001$, F-value 4.2) with success in the final exam.<br><br>**Logbook, learning tool**<br>In addition to acting as a logbook, use of the portfolio enhanced the learning process during the course.	Portfolios support the personal and professional development of medical students. A portfolio clarifies the learning goals and helps students to monitor how these goals are achieved. A portfolio encourages constant self-reflection.

				questions about what they had learned and evaluated the performance of the teacher. They also indicated their general evaluation of the course and the portfolio itself. The teachers listed the 13 most important skills to be learned during the course. The students were asked to evaluate their own development on a scale of 0-5 before and after the course. A content analysis was performed on all the texts the students produced, and all quantitative variables were coded.		**+ve perceptions of portfolio**<br>Students' attitudes towards the portfolio were mainly positive. students appreciated the departmental interest in their learning process.	
27	Maughan, T. S.<br>Finlay, I. G.<br>Webster, D. J.<br>(2001)	Portfolio learning with cancer patients: an integrated module in undergraduate medical education	Descriptive		**Assessment tool**<br>• formative and summative evaluations<br><br>**Reflective tool**<br><br>**Improve interactions with patients (attitudes, skills and knowledge)**<br>• communication skills<br>• dealing with sad and difficult situations<br>• knowledge of cancer	**three key learning areas in medicine: attitudes, skills and knowledge.**<br>*Attitudes* Seeing the medical process from the patient's perspective<br>*Skills* Breaking bad news Coping with their own emotional reactions to sad and difficult situations *Knowledge* The natural history of cancer Cancer screening and diagnosis Staging and metastasis Treatment options and decision making Symptom control<br><br>**formative and summative evaluations**<br>two formative criteria: their interactions with the patient and their contribution to the tutorials; and summatively through the portfolio.	Our observation has been that the students find the project a very rewarding experience that opens their eyes to many aspects of medicine and life; it has demonstrated positive learning outcomes [20]. Students gain an unrivalled insight into a patient's perspective and often then reorientate their own educational priorities as they continue in their medical training.
28	McBride, J. M.<br>Prayson, R. A.<br>(2008)	Development of a synergistic case-based	Descriptive		**Teaching Tool**<br><br>**Assessment Tool**<br>**• formative evalaution**	**Assessment Tool**<br>**• formative evaluation**<br>**• summative evaluation**	

		microanatomy curriculum			**• summative evaluation**<br>**• self evaluation**<br><br>**Benefits of such an assessment tool**	utilization of a portfolio system where the assessment pieces are continuously integrated as part of written formative and summative assessments.<br><br>**• self evaluation**<br>portfolio system as methods for students to<br>self evaluate their performance with regard to core competen-cies.<br><br>**Benefits of such an assessment tool**<br>Portfolios and other competency-based assessment tools are used in several areas of medical education (Snadden and Thomas, 1998; Driessen et al., 2003) which provide both the learner and advising faculty with evi-<br>dence of the student's strengths and weakness rather than anamorphous numerical rating	
29	Mejicano, G. C. Bumsted, T. N. (2018)	Describing the Journey and Lessons Learned Implementing a Competency-Based, Time-Variable Undergraduate Medical Education Curriculum		Oregon Health & Science University School of Medicine launched a completely new undergraduate medical education curriculum in 2014. This initiative dramatically transformed the MD degree program, changing the instructional content taught, the pedagogical methods used by the faculty, and the methods of assessment, and it added new elements such as academic coaching and programmatic entrustment to the program.	**e-portfolio use**<br>**Assessment tool**<br><br>**Tracking students progession**<br><br>**Used with coaching/mentoring**<br><br>**Supplement on learning**	One of the most exciting and impactful aspects to date of this curricular transformation has been the deliberate implementation of a competency-based framework that incorporates frequent assessment, tracking of student progression using an electronic portfolio, and academic coaching to optimize learning and customize curricular elements for each student	This article describes the school's progress to date in its curricular transformation and articulates lessons learned thus far in driving substantive and dramatic institutional changes that profoundly impact students, faculty, and administrators in one academic health center.
30	Michels, N. R. Avonts, M.	Content validity of workplace-	Mixed methods	We reviewed 120 workplace portfolios at three medical	**Assessment tool**	**Content Validity of Portfolios**	This study supports the growing body of evidence that a portfolio can

	Peeraer, G.<br>Ulenaers, K.<br>Van Gaal, L. F.<br>Bossaert, L. L.<br>Driessen, E. W.<br>Muijtjens, A. M.<br>De Winter, B. Y.(2016)	based portfolios: A multi-centre study		universities (Belgium and the Netherlands). To validate their content, we developed a Validity Inventory for Portfolio Assessment (VIPA) based on the CanMEDS roles. Two raters evaluated each portfolio and indicated for each VIPA item whether the portfolio provided sufficient information to enable satisfactory assessment of the item. We ran a descriptive analysis on the validation data and computed Cohen's Kappa to investigate interrater agreement.	**Used with coaching**<br><br>**Content Validity of Portfolios**	portfolios adequately covered the items pertaining to the communicator (90%) and professional (87%) roles. portfolios involved are competence-based and align with the curricular context and the workplace setting.	be a useful tool for assessing the CanMEDS roles and competencies. The health advocate role, however, was less well represented in portfolio content. We established that careful advance determination of the portfolio blueprint and aims, while selecting the appropriate assessment instruments that fit the intended competencies, can greatly enhance content validity.
31	Möller, R.<br>Ringsted, C.<br>Danielsen, N.<br>(2021)	[Portfolio - a tool for making learning and competence development visible]			**Logbook**<br><br>**Teaching tool**<br><br>**Assessment tool**<br><br>**e-portfolio use**<br><br>**Portfolio structure**<br><br>**Guiding& supporting users aid in success of portfolios**	Portfolio used in education can be defined as a collection of documentation of performed learning activities, feedback, and progress.<br><br>**e-portfolio use**<br>Currently, the documentation is electronic, hence the term e-portfolio is used.<br><br>**Portfolio structure**<br>The portfolio must have a clear purpose and be aligned with the learning outcomes of the program.<br><br>**Guiding& supporting users**<br>To be successfully implemented a portfolio must be an integral part of the education with defined tasks for both the students and the teachers. Students and teacher support in how to use the portfolio is essential especially in the beginning of the program	

32	Müller, S. Dahmen, U. Settmacher, U. (2018)	[Application of the Objective Structured Clinical Examination (OSCE) in German Medical Schools: An Inventory]	Mixed methods	From June to September 2015, the implementation of the OSCE in all 36 German medical schools was investigated using semi-structured telephone interviews and email correspondence. The areas of focus comprised implementation of the OSCE, and number and type of performance records according to the medical licensing regulations or involved disciplines. Following collection, data were analysed quantitatively and qualitatively.	**Assessment Tool**	**Assessment Tool**<br>Nearly every school (94%) has introduced the OSCE into its assessment portfolio, however, to varying extents.	The results reported in this paper confirm the widespread introduction of the OSCE assessment in German medical schools. However, the implementation remains heterogeneous with respect to the scope, schools and individual disciplines involved in the process. In order to ensure extensive clinical competence of prospective physicians the application of the OSCE should be broadened. For this purpose, further information to convince medical school staff is still required.
33	Murray, C. Sandars, J.(2009)	E-learning in medical education: Guide supplement 32.2--practical application	Qualitative	Our new web-based e-Portfolio was designed to evaluate surgical practical knowledge and skills targets.	**e-portfolio use**<br><br>**Assessment tool**<br>• self assessment<br><br>**+ve perception of portfolio**<br><br>**Improved understanding of material through feedback**<br><br>**Portfolio guided students learning**<br><br>**Highlighted knowledge gaps to tutor**<br><br>**Increased accessibility to tutor**	used the e-Portfolio, of which 87% reported that they understood the methodology of the portfolio.<br><br>**Improved understanding through feedback**<br>All students reported an improved understanding of their learning objectives resulting from the numerical visualization of progress, all students reported that the quantitative feedback encouraged their learning,<br><br>**Increased accessibility to tutor**<br>79% of students felt that their teachers were more available	Medical students reported that use of an electronic portfolio that provided quantitative feedback on their progress was useful when the number and complexity of targets were appropriate, but not when the portfolio offered only formative evaluations based on reflection. Students felt that use of the e-Portfolio guided their learning process by indicating knowledge gaps to themselves and teachers.
34	O'Brien, C. L. Sanguino, S. M. Thomas, J. X. Green, M. M. (2016)	Feasibility and Outcomes of Implementing a Portfolio Assessment	Qualitative	Five competencies were selected for a preclerkship summative portfolio review. Students submitted reflections on their performance. In 2014, four	**Assessment tool for competency**<br><br>**Identifying knowledge gaps**	**Assessment tool for competency**<br>The majority of students achieved the "progressing toward competence"	Identification of these students allows for intervention and early remediation.

		System Alongside a Traditional Grading System		clinical faculty members participated in standard-setting activities and used expert judgment and holistic review to rate students' competency achievement as "progressing toward competence," "progressing toward competence with some concern," or "progressing toward competence pending remediation." Follow-up surveys measured students' and faculty members' perceptions of the process.	**Early remediation**	benchmark in all competency areas.<br><br>**Identifying knowledge gaps** allowed faculty to identify students with a concerning rating in a behavioral competency who would not have been identified in a traditional grading system.	
35	O'Sullivan, A. J.<br>Howe, A. C.<br>Miles, S.<br>Harris, P.<br>Hughes, C. S.<br>Jones, P.<br>Scicluna, H.<br>Leinster, S. J.<br>(2012)	Does a summative portfolio foster the development of capabilities such as reflective practice and understanding ethics? An evaluation from two medical schools	Quantitative	A questionnaire was designed to evaluate undergraduate medical students’ experiences of completing a portfolio at two medical schools.	**Reflective practice**<br>• developed reflective skills<br><br>**Developing Skills**<br>• communication skills<br><br>**+ve perception of portfolio use**<br><br>**Improved understanding of ethical and legal principles – self-directed learning**<br><br>**Assessment tool to ensure educational goals and competencies are met**<br><br>**logbook**	**Reflective practice** highest ranking for the portfolio as a trigger for reflective practice. 63% of students agreed their portfolio helped them develop reflective practice skills<br><br>**communication skills** 34% of students thought the portfolio helped them to develop effective communication.	Students perceive portfolio preparation as an effective learning tool for the development of capabilities such as understanding ethical and legal principles and reflective practice, whereas other capabilities such as effective communication require complementary techniques and other modes of assessment.

36	Oudkerk Pool, A.<br>Jaarsma, A. D. C.<br>Driessen, E. W.<br>Govaerts, M. J. B. (2020)	Student perspectives on competency-based portfolios: Does a portfolio reflect their competence development?	Qualitative	Students uploaded performance data in a competency-based portfolio. During one clerkship period, twelve students also recorded an audio diary in which they reflected on experiences and feedback that they perceived to be indicants of their competence development. Afterwards, these students were interviewed to explore the extent to which the performance documentation in the portfolio corresponded with what they considered illustrative evidence of their development. The interviews were analyzed using thematic analysis.	**Assessment tool**<br><br>**Perception of portfolio use has influence on effectiveness of portfolios**<br><br>**Influenced learning and assessment**<br><br>**Snapshots of competence development**	**Perception of portfolio use**<br>Portfolios provide an accurate but fragmented picture of student development. Portfolio documentation was influenced by tensions between learning and assessment, student beliefs about the goal of portfolios, student performance evaluation strategies, the learning environment and portfolio structure.<br><br>**Snapshots of competence development**<br>although students felt that performance evaluations documented in their portfolio were fairly representative, they also perceived these to form a rather fragmented picture of their actual development. The portfolios provided snapshots rather than a complete picture of the student's developmental trajectory.	This study confirms the importance of taking student perceptions into account when implementing a competency-based portfolio. Students would benefit from coaching on how to select meaningful experiences and performance data for documentation in their portfolios. Flexibility in portfolio structure and requirements is essential to ensure optimal fit between students' experienced competence development and portfolio content.
37	Rees, C.<br>Sheard, C.(2004)	Undergraduate medical students' views about a reflective portfolio assessment of their communication skills learning	Quantitative	178 second-year medical students at the University of Nottingham completed the 18-item reflective portfolio questionnaire (RPQ) ($\alpha = 0.716$) and a personal details questionnaire three days before submitting their portfolio assessment for communication skills. Data were analysed using univariate and multivariate statistics on SPSS Version 10.0.	**Reflective tool**<br><br>**Developed reflective skills**<br><br>**perceptions of portfolios influence effectiveness**	**Developed reflective skills**<br>Significant relationships existed between RPQ total scores and students' ratings of their reflection skills ($r_s = 0.322$, $P < 0.001$),<br><br>**perceptions of portfolios influence effectiveness**<br>Students with more positive views about reflective portfolios were more likely to rate their reflection skills as good, receive better marks for their portfolio assessment, and be more	This study begins to highlight preclinical medical students' views about reflective portfolios. However, further research is required using qualitative studies to explore students' views in depth. Medical educators should be encouraged to consider introducing portfolios as a method of formative and summative assessment earlier in the medical curriculum.

						confident building another portfolio.	
38	Reyes, D.<br>Isbej, L.<br>Uribe, J.<br>Ruz, C.<br>Pizarro, M.<br>Walker, R.<br>Pérez-Cruz, P.<br>Maldonado, A.<br>Robles, C.<br>Latorre, G.<br>Ivanovic-Zuvic, D.<br>Figueroa, C.<br>González, A.<br>Cotoras, P.<br>Núñez, C.<br>Labarca, J.<br>Riquelme, A.(2019)	[Educational impact after 10 years of implementation of a portfolio for undergraduate medical students]	Qualitative	The development and implementation of a portfolio for 4th-year undergraduate medical student was analyzed. Its design, teaching and learning methodologies, results and perceptions of students and teachers were assessed. The educational impact was measured using Kirkpatrick's levels.	**Logbook**<br><br>**+ve percpetions of portfolios from students and tutors**<br><br>**Improved learning and school performance**<br><br>**Tracked clinical competencies and professionalism**<br><br>**Assessment tool**<br><br>**Reflective tool**<br>**• feedback**	**Tracked clinical competencies and professionalism**<br>The overall student's perception was positive, highlighting the development of critical analysis, clinical reasoning and professionalism.	This portfolio is a project with a high educational impact, with a favorable perception by students and tutors, excellent results related to grades, stimulating both scientific writing and reflective practice.
39	Ross, S.<br>Maclachlan, A.<br>Cleland, J.<br>(2009)	Students' attitudes towards the introduction of a Personal and Professional Development portfolio: potential barriers and facilitators	Qualitative	This was a qualitative, focus group study. All focus groups were taped and transcribed verbatim, and anonymised. The transcripts were analysed inductively, using framework analysis.	**Perception of portfolio by students about a prospective Professional and Personal Development (PPD) portfolio**<br><br>**Teaching tool**<br><br>**Assessment Tool**<br>• could result in biased entries<br><br>**Reflective learning**<br><br>**Advantages to use of portfolios**<br><br>**Barriers to use of Portfolios**<br>• Better portfolio structure<br>• More instructional support needed for Portfolio use	**Barriers to use of Portfolios Better structure, More support & guidance needed for Portfolio use**<br>most students wanted a clear explanation of the purpose of a portfolio, in a format which incorporated opportunity for questions, such as a lecture or tutorial.<br><br>**Concerns over Commitment to Portfolio**<br>many students about the potential workload involved in a portfolio and how this would be integrated with their other commitments<br><br>**Assessment Tool: results in biased entries**	the results of this study provide useful information to inform the optimal introduction of PPD portfolios aimed at supporting medical students in reflective learning.

					• Concerns over Commitment to Portfolio<br><br>**Gaps**	s discussed how assessment of portfolio content may encourage students to write socially acceptable or perceived "correct" answers<br><br>**Reflective Learning**<br>negative views about reflective learning and whether this could be taught and assessed, believing formal assessment could foster socially acceptable content. Participants revealed little understanding of reflective learning and its potential benefits.<br><br>**Advantages**<br>The potential advantages in having skills and material to aid in completing foundation programme application were widely perceived as important. portfolio would prepare the students for when they came to use portfolios in future (when working as a doctor) and that they could use the portfolio as a useful record of nonacademic achievements such as extra-curricular activities.<br><br>benefits were perceived in terms of concrete outcomes such as job applications or making post graduate life easier.<br><br>**Gaps**<br>Understanding of portfolio based learning as a learning tool and for personal development was lacking.	

40	Sánchez Gómez, S. Ostos, E. M. Solano, J. M. Salado, T. F. (2013)	An electronic portfolio for quantitative assessment of surgical skills in undergraduate medical education	Quantitative	Our new web-based e-Portfolio was designed to evaluate surgical practical knowledge and skills targets. Students recorded each activity on a form, attached evidence, and added their reflections. Students self-assessed their practical knowledge using qualitative criteria (yes/no), and graded their skills according to complexity (basic/advanced) and participation (observer/assistant/independent). A numerical value was assigned to each activity, and the values of all activities were summated to obtain the total score. The application automatically displayed quantitative feedback. We performed qualitative evaluation of the perceived usefulness of the e-Portfolio and quantitative evaluation of the targets achieved.	**e-portfolio use**<br>• Increased interaction with tutors<br><br>**perceptions of portfolio**<br>•understood methodology of portfolio<br><br>**Reflective Learning**<br><br>**Assessment tool**<br>**• formative evaluation**<br><br>**Benefits as a learning Tool**<br>• improve learning objectives<br>• encouraged learning	**Benefits as a learning Tool**<br>improved understanding of their learning objectives resulting from the numerical visualization of progress all students reported that the quantitative feedback encouraged their learning<br><br>**Increased interaction with tutors**<br>79% of students felt that their teachers were more available because they were using the e-Portfolio.<br><br>**Reflective learning**<br>Only 51.3% of students reported that the reflective aspects of learning were useful.	Medical students reported that use of an electronic portfolio that provided quantitative feedback on their progress was useful when the number and complexity of targets were appropriate, but not when the portfolio offered only formative evaluations based on reflection. Students felt that use of the e-Portfolio guided their learning process by indicating knowledge gaps to themselves and teachers.
41	Shamim, M. S. Zubairi, N. A. Sayed, M. H. Gazzaz, Z. J. (2016)	Innovation in ethics and professionalism course: Early experience with portfolio-workbook	Qualitative	This mixed method study was conducted at the King Abdulaziz University, Jeddah, Saudi Arabia, in 2014, and comprised fourth-year medical students. The "portfolio-workbook", developed on principles of cognitive load and guided learning theories, contained essential reading material. Learning sessions were also facilitated by teaching tools like role-plays, movie/video clips, vignettes, etc., followed by reflective writing exercises. Feedback questionnaire with open- and closed-ended	**Perception of portfolio use**<br>**• initially hard-> easy**<br><br>**Teaching and learning tool**<br><br>**Assessment tool**	**Perception of portfolio use**<br>considered using portfolio-workbook as difficult initially, However, on completion of module 16(80%) found it easy<br><br>**Teaching and learning tool**<br>Moreover, 17(85%) appreciated it as a learning tool. Besides, 19(95%) students found teaching videos and 13(65%) found open discussions as effective learning tools.<br><br>**Assessment tool**	The use of portfolio-workbook in teaching ethics to undergraduates was found to be encouraging as it generated interest and interaction.

				questions was used to collect data. Quantitative data was analysed for frequencies and percentages. Content analysis was conducted for the open-ended responses.		Portfolio-workbook as an assessment tool was preferred by 19(95%) students.	
42	Shiozawa, T.<br>Glauben, M.<br>Banzhaf, M.<br>Griewatz, J.<br>Hirt, B.<br>Zipfel, S.<br>Lammerding-Koeppel, M.<br>Herrmann-Werner, A.<br>(2020)	An Insight into Professional Identity Formation: Qualitative Analyses of Two Reflection Interventions During the Dissection Course	Qualitative	The Tuebingen Medical Faculty implemented a learning portfolio and a seminar on medical professionalism during the dissection course. The aims of this research project are to get an overview of how students form a professional identity in the dissection course and to compare the content of both their oral and written reflections on the course. A qualitative analysis was conducted of the oral and written reflections on the dissection laboratory experience.	**Portfolio for Reflective Learning**<br>• professional development, • empathy<br>• respect<br>• altruism<br>• compassion<br>• teamwork<br>• self-regulation<br><br>**Reflective Modalities**	**Reflective Learning**<br>students reflected on many topics relevant to professional development, including empathy, respect, altruism, compassion, teamwork, and self-regulation.<br><br>**Reflective Modalities**<br>students who attended the oral reflection wrote significantly more in their written reflection than students who did not. There is, however, no difference in the reflection categories.	
43	Sohrmann, M.<br>Berendonk, C.<br>Nendaz, M.<br>Bonvin, R.<br>Swiss Working Group For Profiles, Implementation<br>(2020)	Nationwide introduction of a new competency framework for undergraduate medical curricula: a collaborative approach	Qualitative	The nationwide implementation of such a competency- and EPA-based approach to medical education is a complex process that represents an important change to the organisation of undergraduate training in the various medical schools.	**e-portfolio use within curriculum for learning**	**e-portfolio use within curriculum**<br>longitudinal integration of an e-portfolio to support the student learning process	The nationwide collaborative approach to define strategies and conditions for the implementation of a new reference framework has allowed to develop a shared understanding of the implications of PROFILES, to promote the establishment of Swiss mapping and e-portfolio communities, and to establish the conditions necessary for ensuring the continuous alignment of the FLE with the evolving medical curricula
44	Supiano, M. A.<br>Fantone, J. C.<br>Grum, C.(2002)	A Web-based geriatrics portfolio to document medical students'	Descriptive	The University of Michigan Medical School is integrating into its curriculum the attitudes, knowledge, and skills that pertain to the care of older individuals using a defined set of core learning outcomes	**e-Portfolio use**<br><br>**Learning Outcomes**<br><br>**Logbook**<br><br>**Self directed learning**	**e-Portfolio use**<br>We have developed an individualized, interactive, Web-based geriatrics portfolio to track the acquisition and mastery of these outcomes for students.	The geriatrics portfolio serves to identify and highlight geriatrics-related content across the four years. Its interactive features make it much more dynamic than a written transcript. Requiring proficiency in learning outcomes related to

		learning outcomes		encompassing all four years. Students will demonstrate proficiency in these outcomes as a graduation requirement. We have developed an individualized, interactive, Web-based geriatrics portfolio to track the acquisition and mastery of these outcomes for students.	**Assessment tool**<br><br>**Tracking Students Progress**	**Learning Outcomes**<br>The portfolio cross-references learning outcomes to specific activities in the curriculum. The activities include content given in lectures, multidisciplinary case discussions, standardized patient instructor (SPI) experiences involving older patients, and specific types of patient encounters during the clinical years.<br><br>**Logbook**<br>The portfolio allows documentation of completion dates of specific activities and the evaluations the student received.<br><br>**Self directed learning**<br>The portfolio is designed to encourage students to take responsibility for their geriatrics education<br><br>**Assessment tool**<br>Several types of evaluation data are provided, some that are specific to an activity (e.g., SPI feedback) and others that provide global assessments of learning outcomes (e.g., attitude surveys)<br><br>**Tracking Students Progress**<br>the Web page can be displayed by the list of outcomes (categorized by attitudes, knowledge, and skills), by medical school year, and by date of completion. In this way,	geriatrics for graduation will clearly convey to students that this information is critically important in their training to become physicians. The individualized evaluation summaries will prove useful to the student because self-directed learning opportunities can be targeted to address weak areas. Evaluation of performances will also aid program directors to appropriately modify the curriculum to address any deficiencies. This innovative Web-based approach to capture learning outcomes that are dispersed throughout a four-year curriculum may also find application in similar curricula (e.g., women's health and end-of-life care).

						students can see at a glance how they are performing and whether they are up-to-date with completing the required outcomes.	
45	Tochel, C.<br>Haig, A.<br>Hesketh, A.<br>Cadzow, A.<br>Beggs, K.<br>Colthart, I.<br>Peacock, H.<br>(2009)	The effectiveness of portfolios for post-graduate assessment and education: BEME Guide No 12	Qualitative	A literature search was conducted for articles describing the use of a portfolio for learning in a work or professional study environment. It was designed for high sensitivity and conducted across a wide range of published and unpublished sources relevant to professional education. No limits for study design or outcomes, country of origin or language were set. Blinded, paired quality rating was carried out, and detailed appraisal of and data extraction from included articles was managed using an online tool developed specifically for the review. Findings were discussed in-depth by the team, to identify and group pertinent themes when answering the research questions.	**Portfolios encouraged reflection**<br><br>**Facilitated engagement with learning**<br>**Used with coaching, mentoring**<br><br>**Perceptions of portfolios by students**<br><br>**Portfolio training**<br><br>**Assessment tool**<br><br>**e-portfolio use might be more effective than paper based portfolios**	**Portfolios encouraged reflection**<br>**Facilitated engagement with learning**<br>Portfolios encouraged reflection in some groups, and facilitated engagement with learning<br><br>there was limited evidence of the influence of a number of factors on portfolio use, including ongoing support from mentors or peers, implementation method, user attitude and level of initial training.<br><br>**Assessment tool**<br>A number of authors explored the reliability and validity of portfolios for summative assessment but reports of accuracy across the disparate evidence base varied.<br><br>**e-portfolio use**<br>There was good evidence that the flexibility of the electronic format brought additional benefits to users, assessors and organisations, and encouraged more enthusiastic use. Security of data remained a high priority issue at all levels, and there was emerging evidence of successful transfer between electronic portfolio systems.	if well implemented, portfolios are effective and practical in a number of ways including increasing personal responsibility for learning and supporting professional development. Electronic versions are better at encouraging reflection and users voluntarily spend longer on them. Regular feedback from a mentor enhances this success, despite competing demands on users' time and occasional scepticism about the purpose of a portfolio. Reports of inter-rater reliability for summative assessments of portfolio data are varied and there is benefit to be gained from triangulating with other assessment methods. There was insufficient evidence to draw conclusions on how portfolios work in interdisciplinary settings.

46	van Schaik, S.<br>Plant, J.<br>O'Sullivan, P.(2013)	Promoting self-directed learning through portfolios in undergraduate medical education: the mentors' perspective	Qualitative	Interviews with faculty members who mentored medical students in portfolio were audio-recorded, transcribed and analysed for themes.	**Self-directed learning**<br><br>**Used with mentoring**<br><br>**Perception of portfolio engagement**	**Self-directed learning**<br>Portfolios allow learners to reflect on their progress, diagnose learning needs and create learning plans, all elements of SDL.<br><br>**Used with mentoring**<br>mentors' definitions of SDL. While mentorship is deemed to be essential for successful portfolio use, it is not known what constitutes effective mentorship in this process.<br><br>**Perception of portfolio engagement**<br>their perceptions of students' engagement with the portfolio<br><br>**Impact of portfolio on mentoring relationship**<br>impact of the portfolio process on the mentoring relationship.	This study revealed tensions between mentors' beliefs regarding the importance of SDL, their own approach to SDL and their perceptions of students' SDL skills. Based on our analysis of these tensions, we recommend both explicit faculty development and institutional culture change for successful integration of SDL in medical education.
47	Watson, E. G.<br>Moloney, P. J.<br>Toohey, S. M.<br>Hughes, C. S.<br>Mobbs, S. L.<br>Leeper, J. B.<br>McNeil, H. P.<br>(2007)	Development of eMed: a comprehensive, modular curriculum-management system	Descriptive	In 2001 the University of New South Wales Faculty of Medicine embarked on designing a curriculum-management system to support the development and delivery of its new, fully integrated, outcome-based, six-year undergraduate medicine program. The Web-enabled curriculum-management system it developed is known as eMed, and it comprises a suite of integrated tools used for managing graduate outcomes, content, activities, and assessment in the new program. The six main tools are a curriculum map, timetable,	**Teaching & Assessment tool in curriculum**<br><br>**e-portfolio use**<br><br>**perception of e-portfolio use**	**perception of e-portfolio use**<br>Evaluation results indicated a high level of user acceptance and approval.	The integration of learning and assessment activities data in the one system gives a depth of curriculum information that is unusual and that allows for data-based decision making. Technologically, eMed helps to keep the medicine program up to date. Organizationally, it strengthens the school's data-driven decision-making process and knowledge network culture.

				student portfolio, peer feedback tool, assessment tracking, and results tools.			
48	Yoo, D. M.<br>Cho, A. R.<br>Kim, S. (2019)	Evaluation of a portfolio-based course on self-development for pre-medical students in Korea	Mixed Methods	The subjects of this study were 97 students of a pre-medical course "Self-development and portfolio I" in 2019. Their learning experience was evaluated through the professor's assessment of portfolios they had submitted, and the program was evaluated based on the responses of 68 students who completed a survey. The survey questionnaire included 32 items. Descriptive statistics were reported for quantitative data, including the mean and standard deviation. Opinions collected from the open-ended question were grouped into categories.	**Need for more Components/ Structure of portfolio**<br><br>**Lack of insufficient content**<br><br>**Perceptions of portfolio use**<br><br>**Focus group activities more effective than lectures**<br><br>**Commitment to portfolio (interfered with other studies)**	**Components/ Structure of portfolio**<br>**Lack of insufficient content**<br>6.2% of the students' portfolios were well-organized, with specific goals, strategies, processes, and self-reflections, while most lacked the basic components of a portfolio (46.4%) or contained insufficient content (47.4%)<br><br>**Focus group activities more effective than lectures**<br>regular portfolio personality assessments (72.1%), team (64.7%), and individual (60.3%) activities were felt to be more appropriate as educational methods for this course, rather than lectures.<br><br>**Commitment to portfolio (interfered with other studies)**<br>individual autonomy needed to be reflected more (66.7%) and that this course interfered with other studies (42.5%).	The findings of this study suggest that standardized samples, guidelines, and sufficient time for autonomous portfolio creation should be provided. In addition, education on portfolio utilization should be conducted in small groups in the future.
49	Zier, K.<br>Wyatt, C.<br>Muller, D.(2012)	An innovative portfolio of research training programs for medical students	Descriptive		**Use of mentoring**	he student–faculty relationship can be extremely rewarding, working with a student helps faculty to improve their mentoring skills and enhance their mentoring portfolio, and mentorship offers an opportunity to identify and	

						nurture talent. Mentors also serve as critically important career role models	
50	Zundel, S. Blumenstock, G. Zipfel, S. Herrmann-Werner, A. Holderried, F. (2015)	Portfolios Enhance Clinical Activity in Surgical Clerks		The study was conducted with a modified pre-post design at the University Hospital of Tuebingen, Germany. Before and after the implementation of the portfolio on April 1, 2013, final-year students (n = 557) who had just finished their surgical clerkship were interviewed with an online questionnaire. A total of 21 basic surgical skills were evaluated.	**Effects of portfolio** **• benefit – enhance clinical activity for surgical clerks in the study cohort.**	there was more clinical activity in the postintervention (portfolio) group.	The implementation of the portfolio did enhance clinical activity for surgical clerks in the study cohort. Nevertheless, overall exposure is still unsatisfactory low for some activities. Additional changes and studies are necessary to further improve surgical education.
